# Supplementary material for: NOXA Accentuates Apoptosis Induction by a Novel Histone Deacetylase Inhibitor
Source: Cancers (Basel). 2023 Jul 17;15(14):3650. doi: 10.3390/cancers15143650 (PMC10377841; doi:10.3390/cancers15143650)
Supplement: Supplementary file 1 [file cancers-15-03650-s001.zip › cancers-2344362-supplementary figures.pdf]

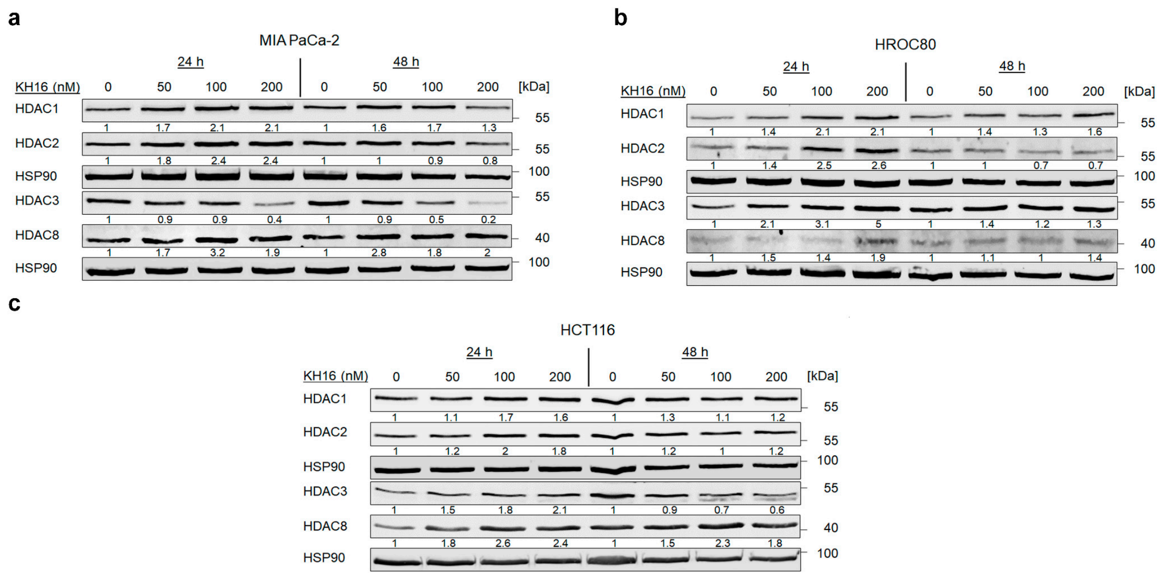

Figure S1. HDAC expression in the presence of KH16. (**a**) Immunoblots of lysates from MIA PaCa-2 cells that were treated with KH16 (50, 100, and 200 nM) for 24 h and 48 h show HDAC1, HDAC2, HDAC3 and HDAC8. (**b**) Immunoblots of lysates from HROC80 cells that were treated with KH16 (50, 100, and 200 nM) for 24 h and 48 h show HDAC1, HDAC2, HDAC3 and HDAC8. (**c**) Immunoblots of lysates from HCT116 cells that were treated with KH16 (50, 100, and 200 nM) for 24 h and 48 h show HDAC1, HDAC2, HDAC3, and HDAC8. HSP90 serves as independent loading control for each membrane. Numbers below the indicated proteins are densitometric analyses of the protein expression normalized to the loading control; protein levels of untreated cells were defined as 1.0 ($n = 2 \pm SD$).

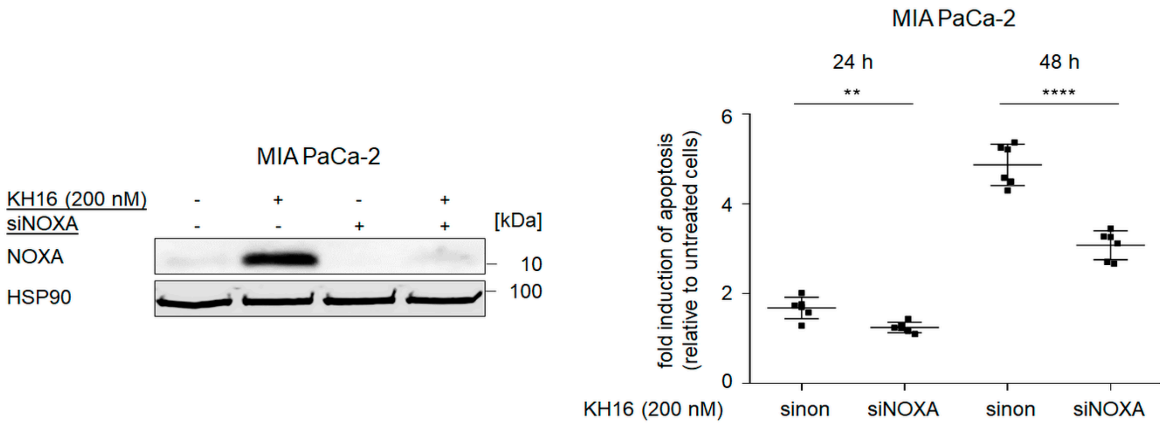

(**a**) (**b**)

Figure S2. Impact of NOXA knock down on the anti-tumor cell potential of KH16. (**a**) Immunoblots of lysates from MIA PaCa-2 cells (with and without NOXA knock down) that were treated with 200 nM KH16 show NOXA. HSP90 serves as loading control for the membrane ($n = 2 \pm SD$). (**b**) Representative flow cytometry dose–response chart of MIA PaCa-2 cells (with and without NOXA knock down) that were treated with 200 nM KH16 for 24 h and 48 h. Cells were stained with annexin-V/PI and measured via flow cytometry. The fold induction of apoptosis normalized to untreated cells was calculated ($n = 3 \pm SD$; Student's t-test: ** $p \leq 0.01$; **** $p \leq 0.0001$).
