# Supplementary material for: NOXA Accentuates Apoptosis Induction by a Novel Histone Deacetylase Inhibitor
Source: Cancers (Basel). 2023 Jul 17;15(14):3650. doi: 10.3390/cancers15143650 (PMC10377841; doi:10.3390/cancers15143650)

Original western blots of Figure 1

MIA PaCa-2 → 3*HDACi [ac-H3..24h]

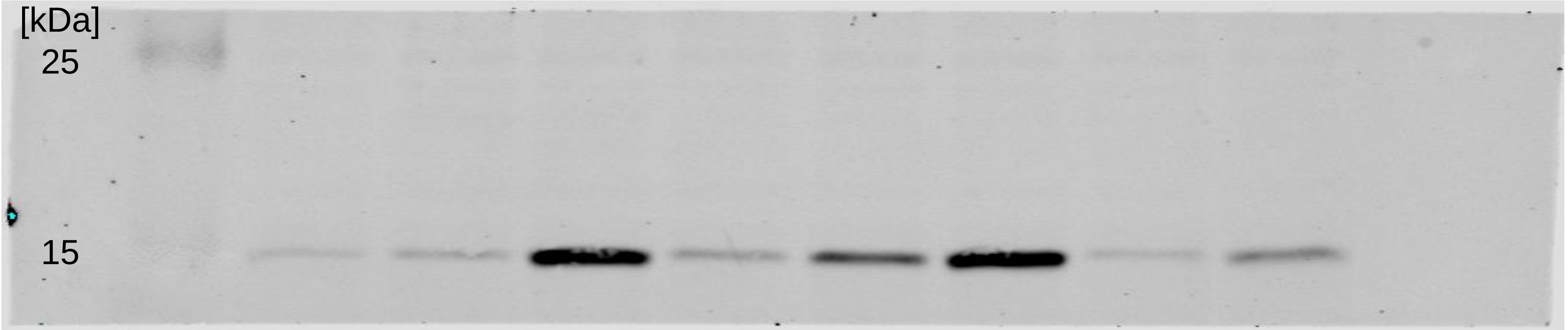

MIA PaCa-2 → 3*HDACi [ac-tubulin..24h]

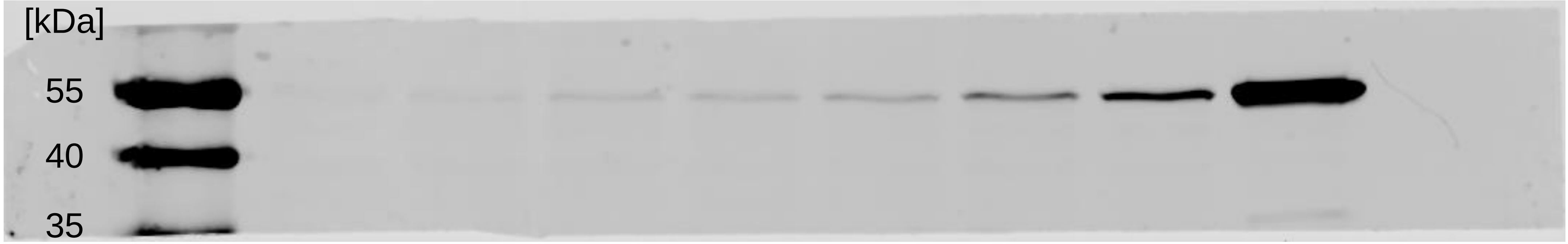

MIA PaCa-2 → 3*HDACi [HSP90 for ac-H3 & ac-tubulin..24h]

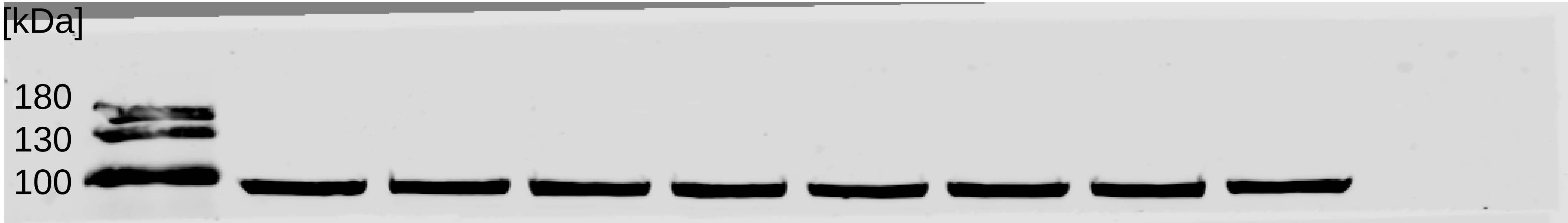

MIA PaCa-2 → 3*HDACi [ac-H3(K9)..24h]

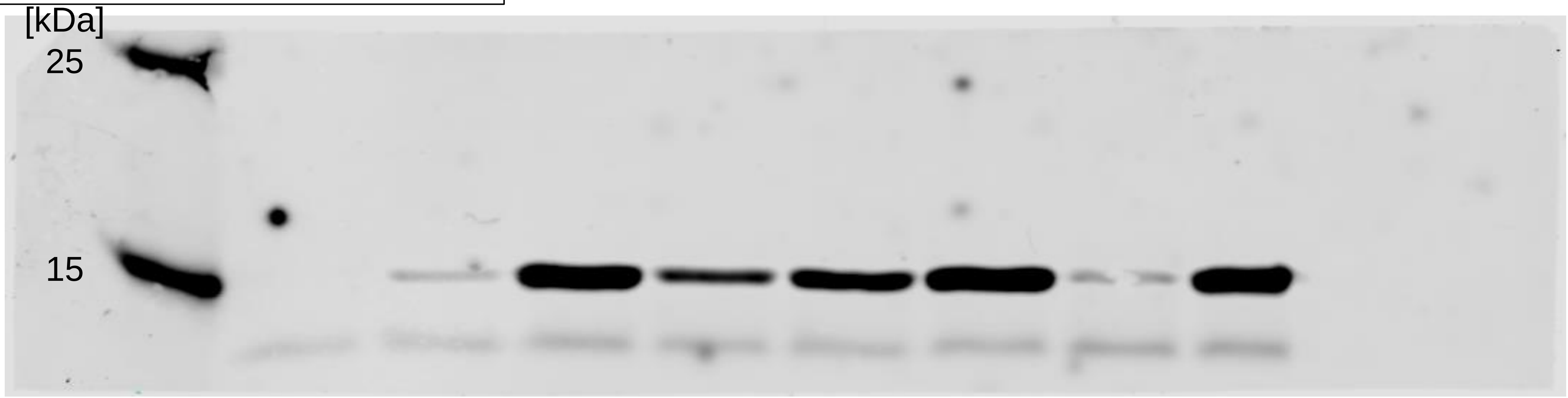

MIA PaCa-2 → 3*HDACi [H3..24h]

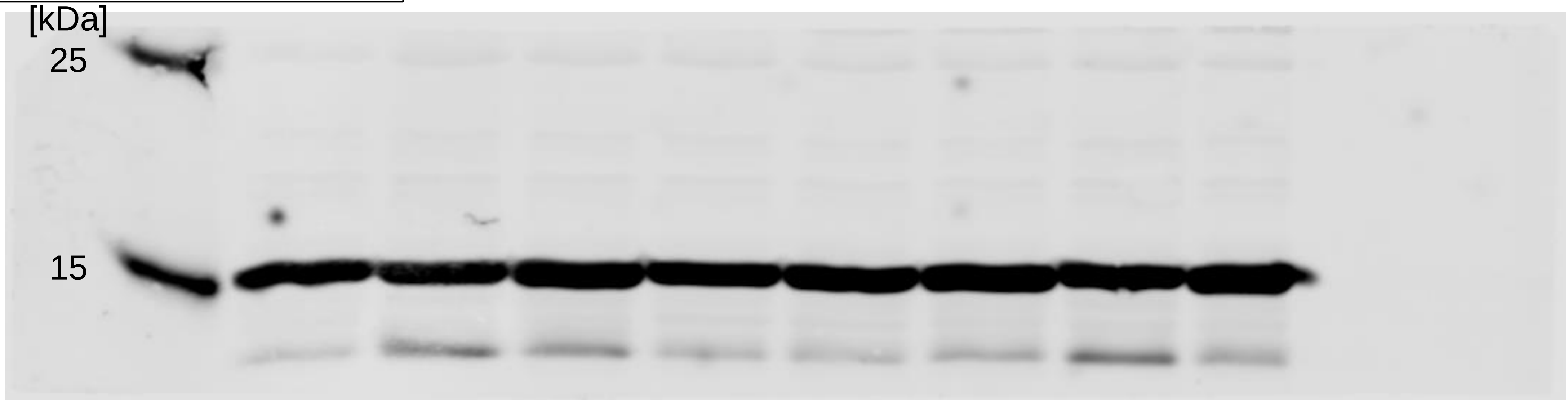

MIA PaCa-2 → 3*HDACi [HSP90 for ac-H3(K9)..24h]

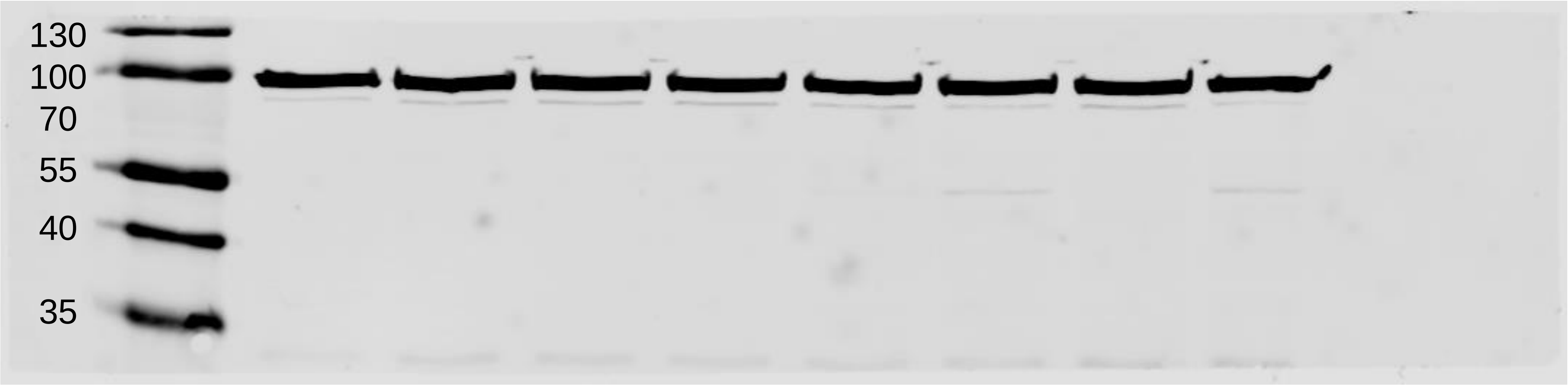

MIA PaCa-2 → 3*HDACi [ac-H3(K18)..24h]

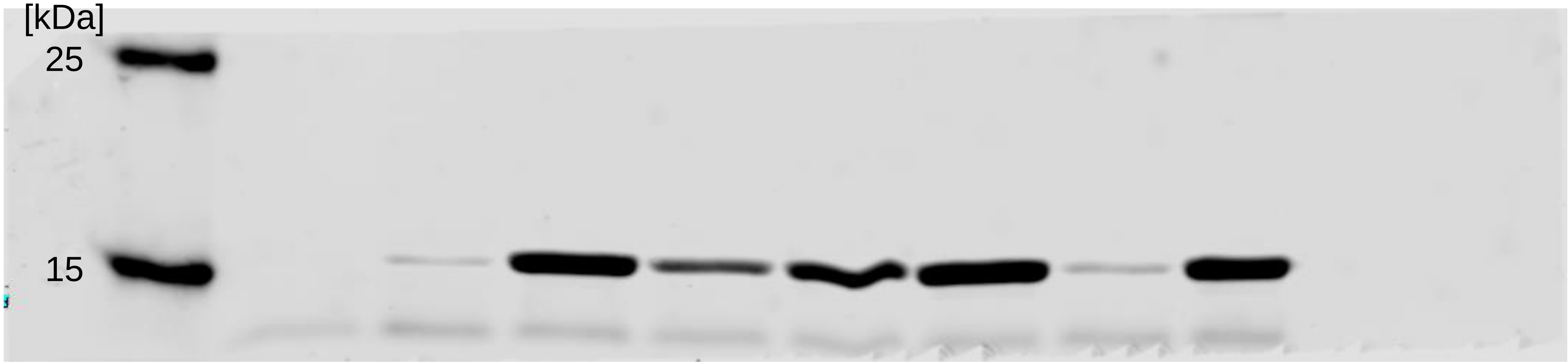

MIA PaCa-2 → 3*HDACi [GAPDH for ac-H3(K18)..24h]

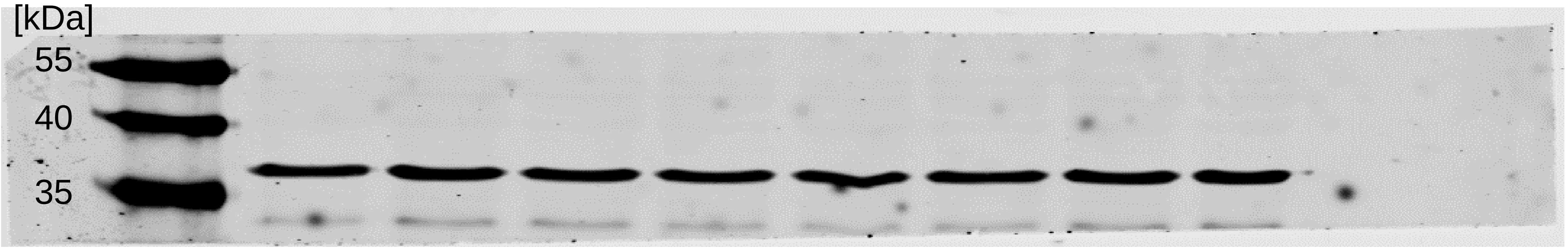

MIA PaCa-2 → 3*HDACi [ac-H3(K27)..24h]

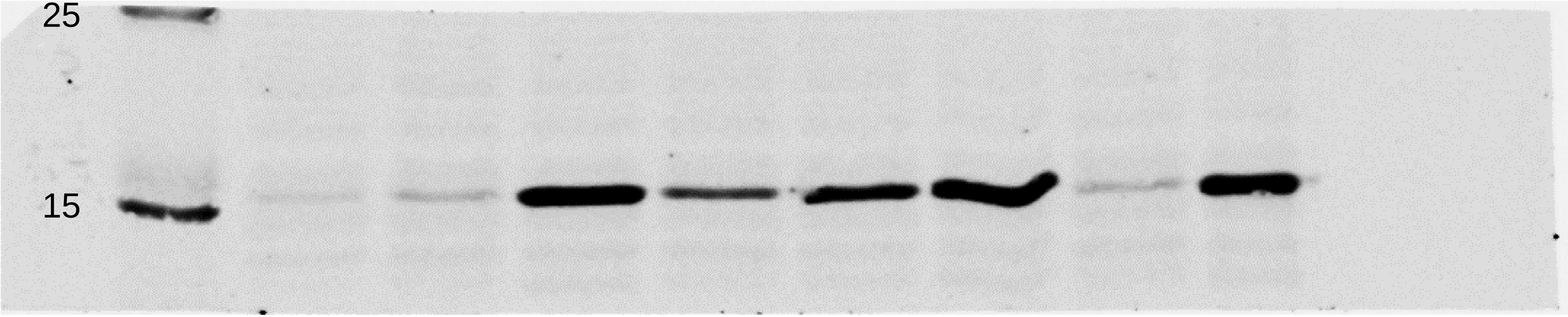

MIA PaCa-2 → 3*HDACi [GAPDH for ac-H3(K27)..24h]

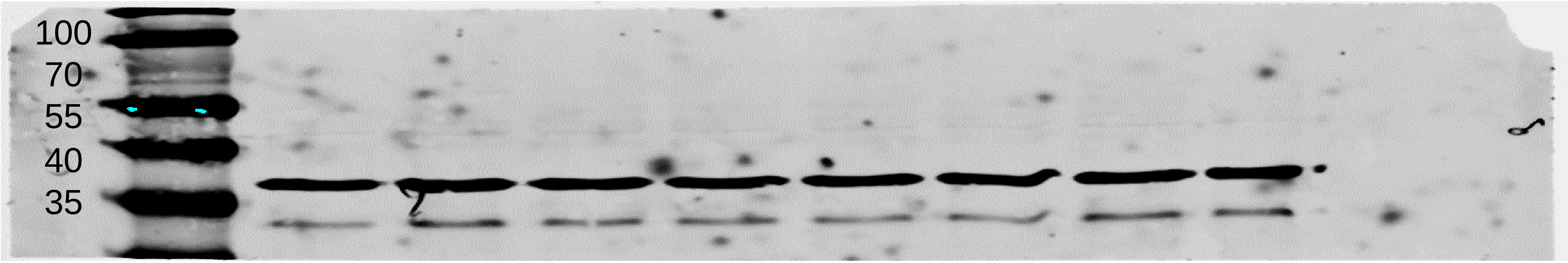

MIA PaCa-2 → 3*HDACi [p21..24h]

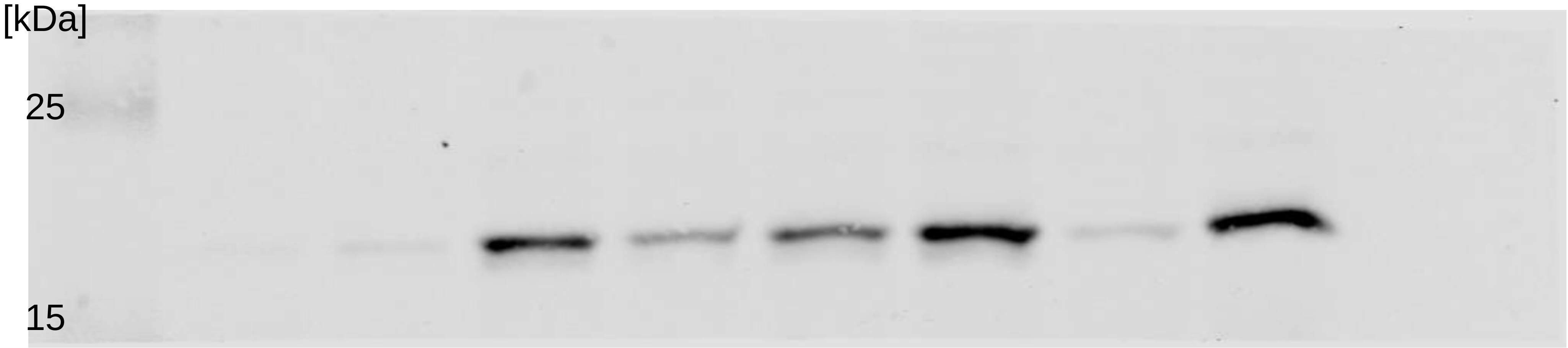

MIA PaCa-2 → 3*HDACi [HSP90 for p21..24h]

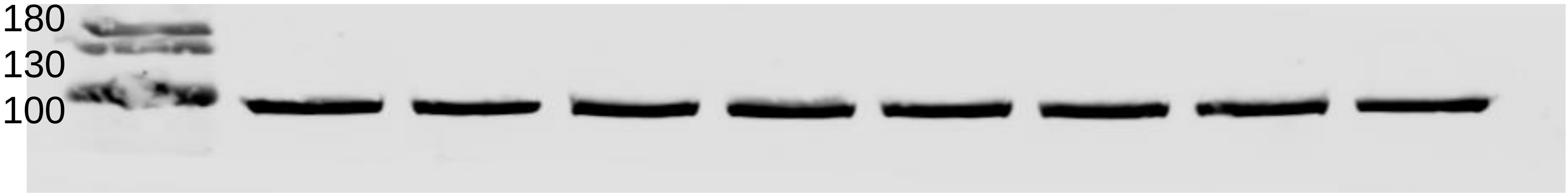

MIA PaCa-2 → 3*HDACi [ac-H3..48h]

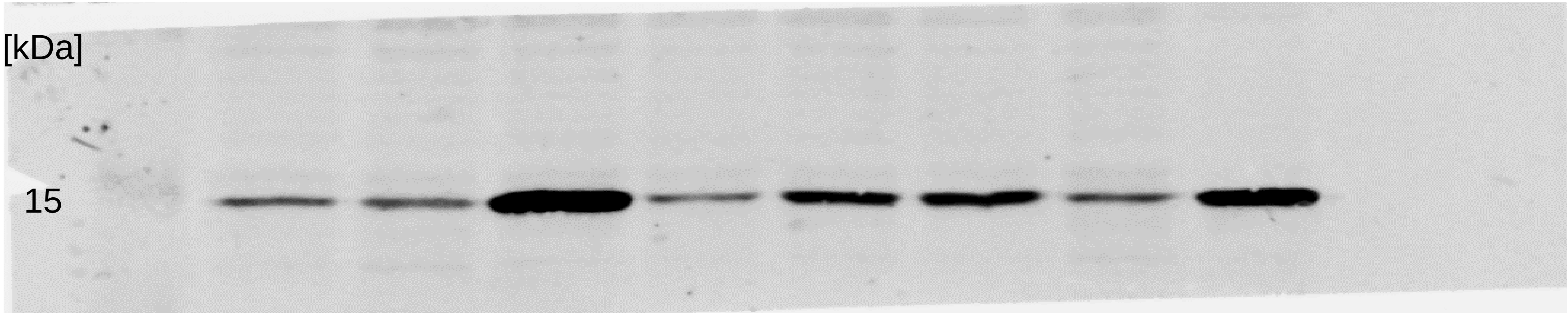

MIA PaCa-2 → 3*HDACi [ac-tubulin..48h]

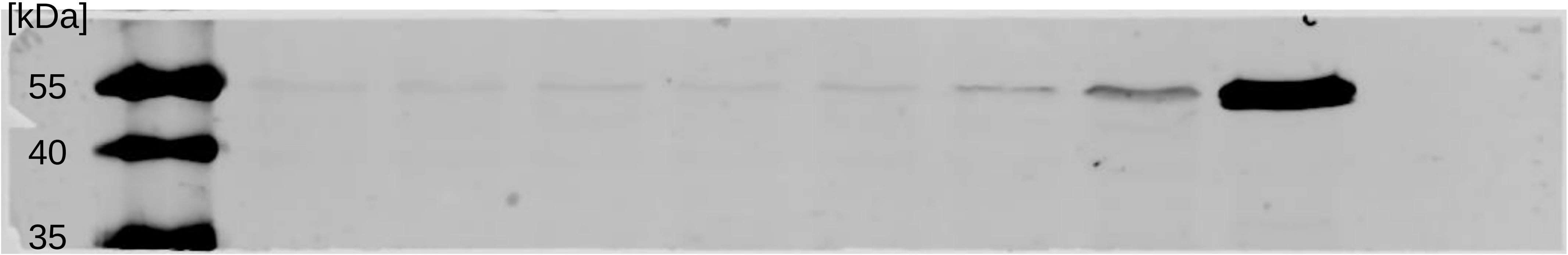

MIA PaCa-2 → 3*HDACi [HSP90 for ac-H3 & ac-tubulin..48h]

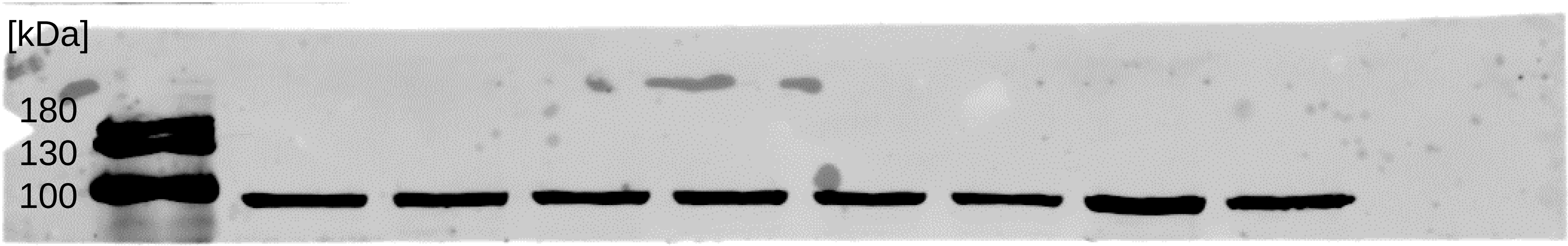

MIA PaCa-2 → 3*HDACi [ac-H3(K9)..48h]

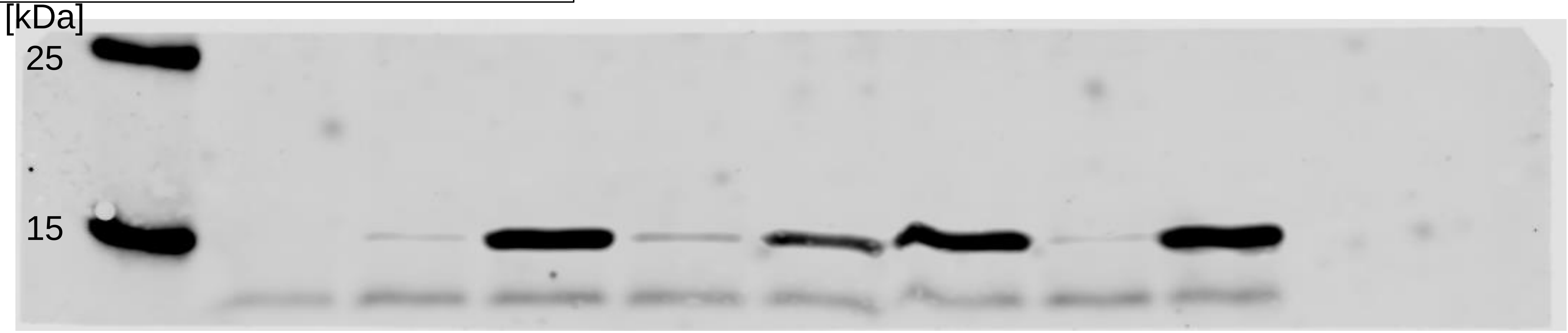

MIA PaCa-2 → 3*HDACi [H3..48h]

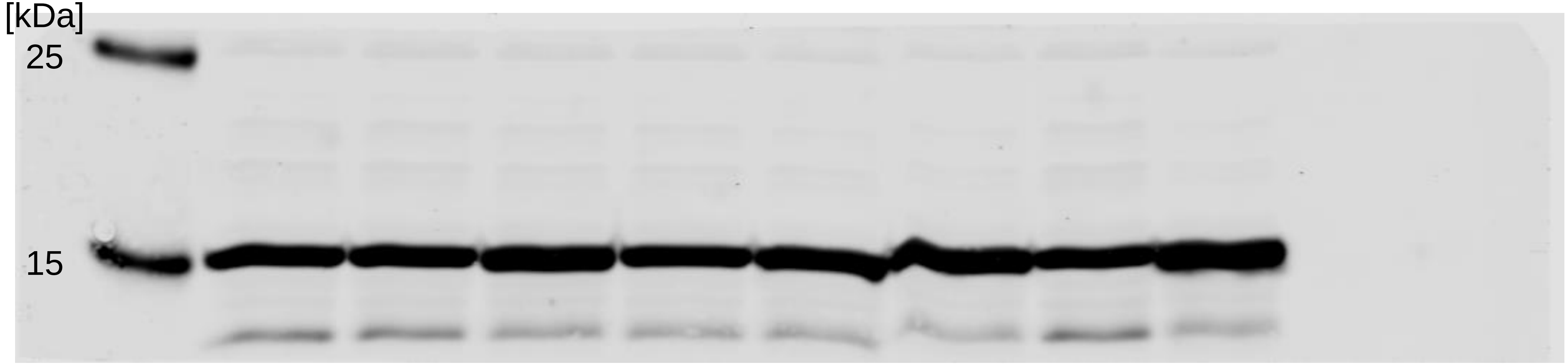

MIA PaCa-2 → 3*HDACi [HSP90 for ac-H3(K9)..48h]

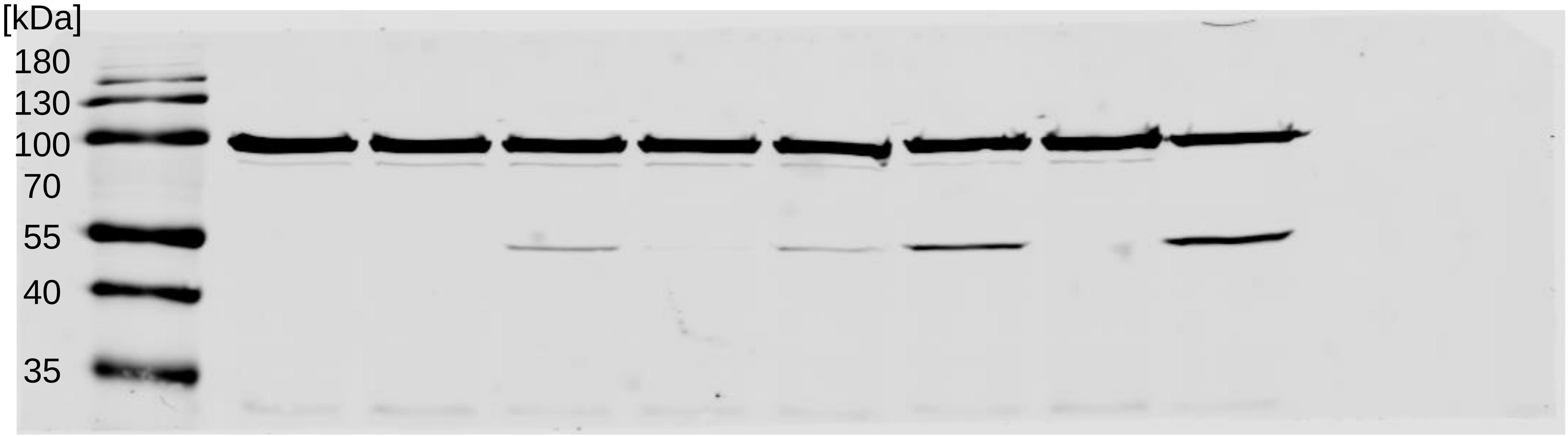

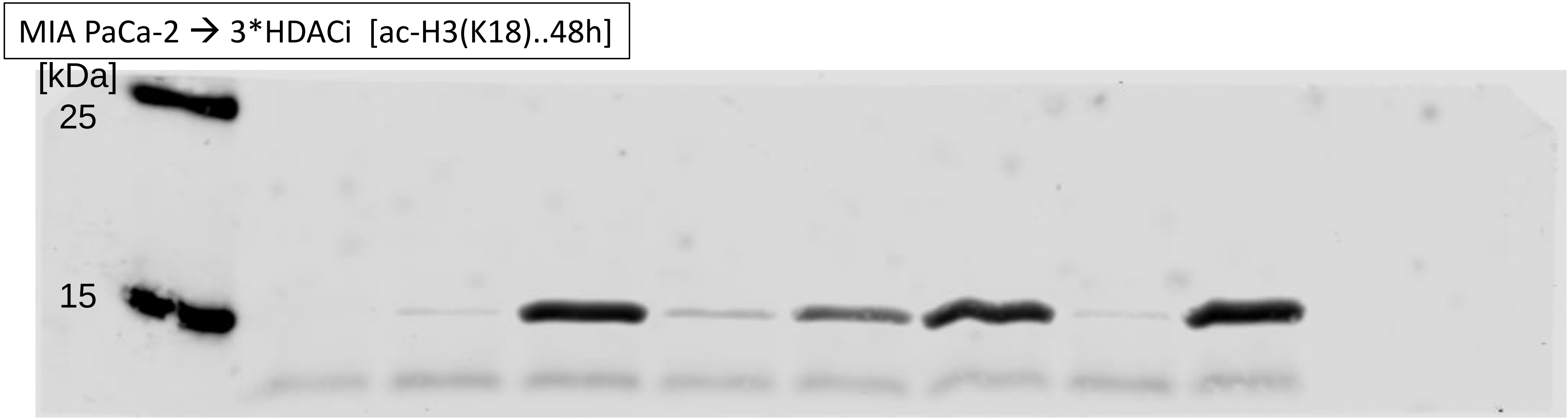
MIA PaCa-2 → 3*HDACi [ac-H3(K18)..48h]
[kDa]
25
15

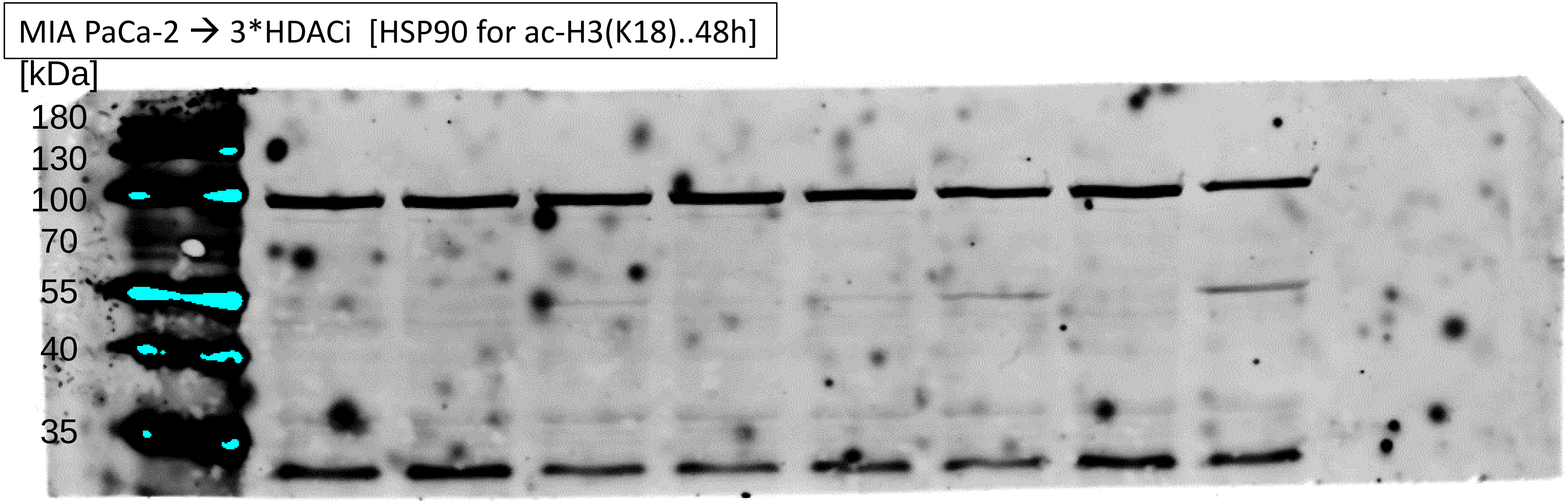
MIA PaCa-2 → 3*HDACi [HSP90 for ac-H3(K18)..48h]
[kDa]
180
130
100
70
55
40
35

MIA PaCa-2 → 3*HDACi [ac-H3(K27)..48h]

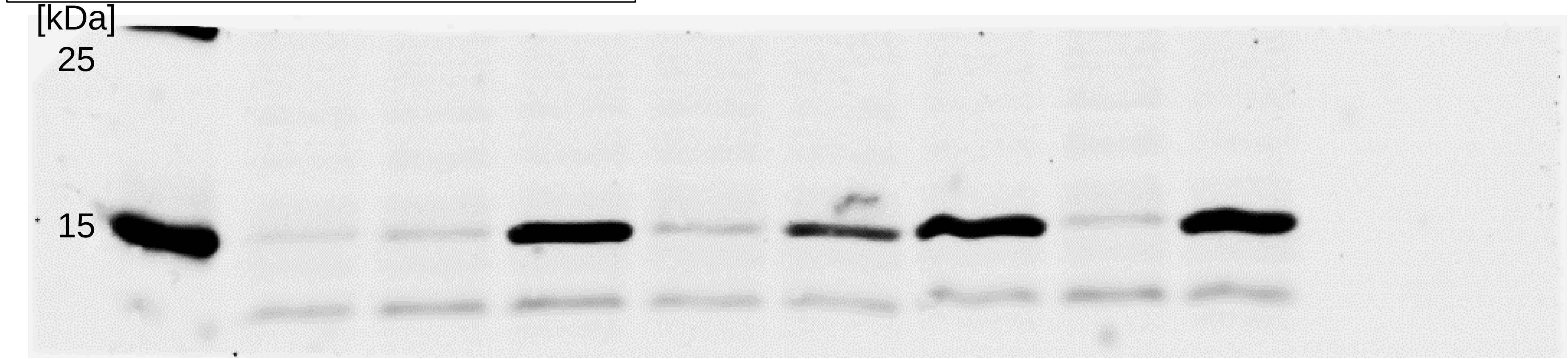

MIA PaCa-2 → 3*HDACi [GAPDH for ac-H3(K27)..48h]

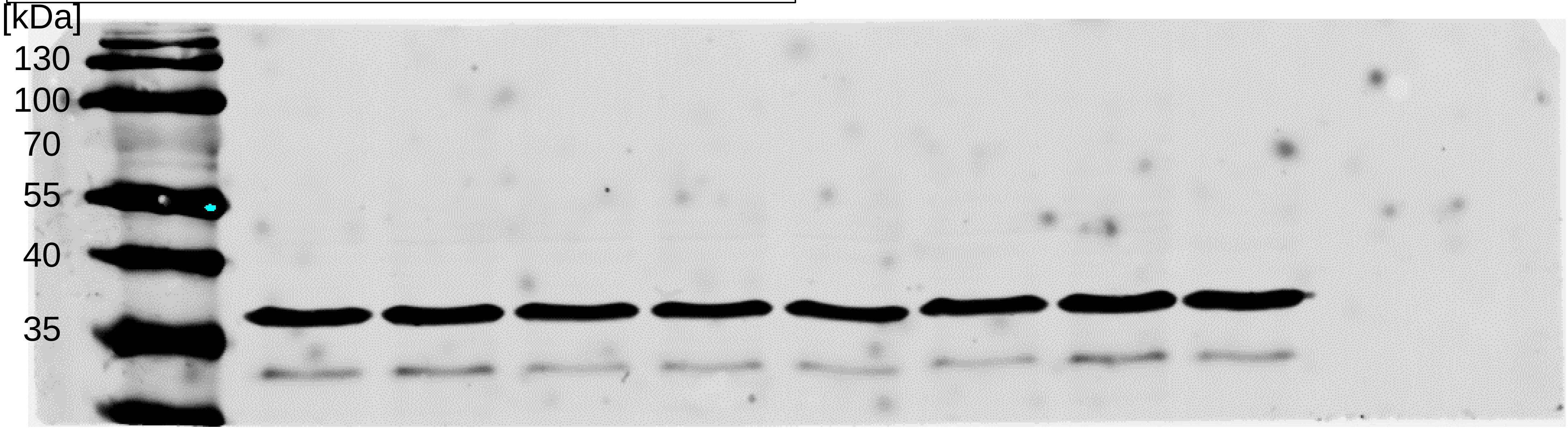

MIA PaCa-2 → 3*HDACi [p21..48h]

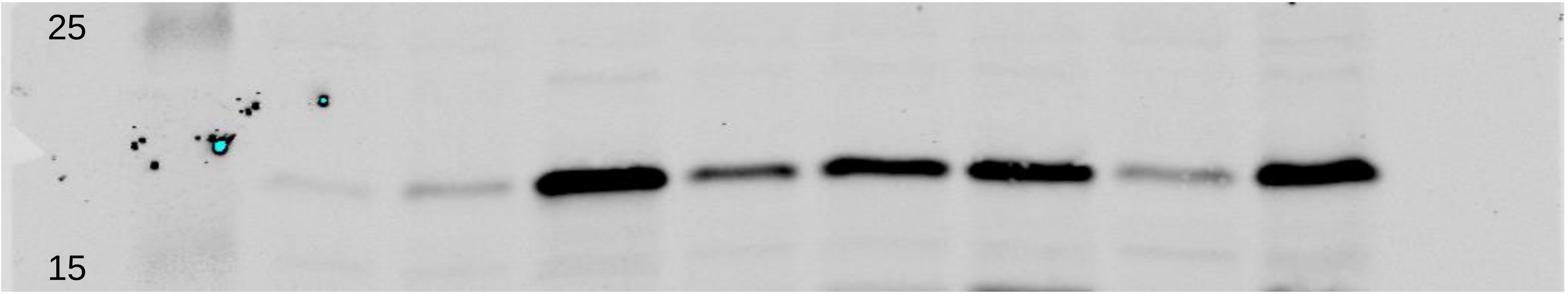

MIA PaCa-2 → 3*HDACi [HSP90 for p21..48h]

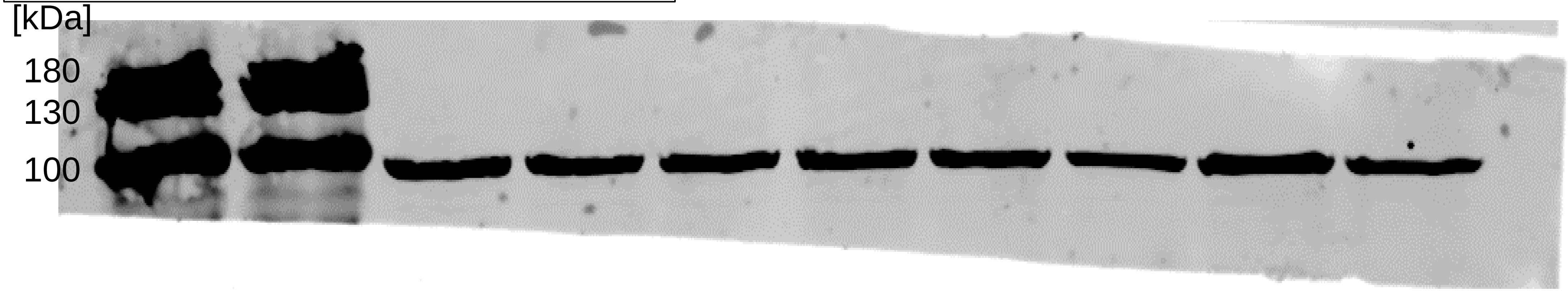

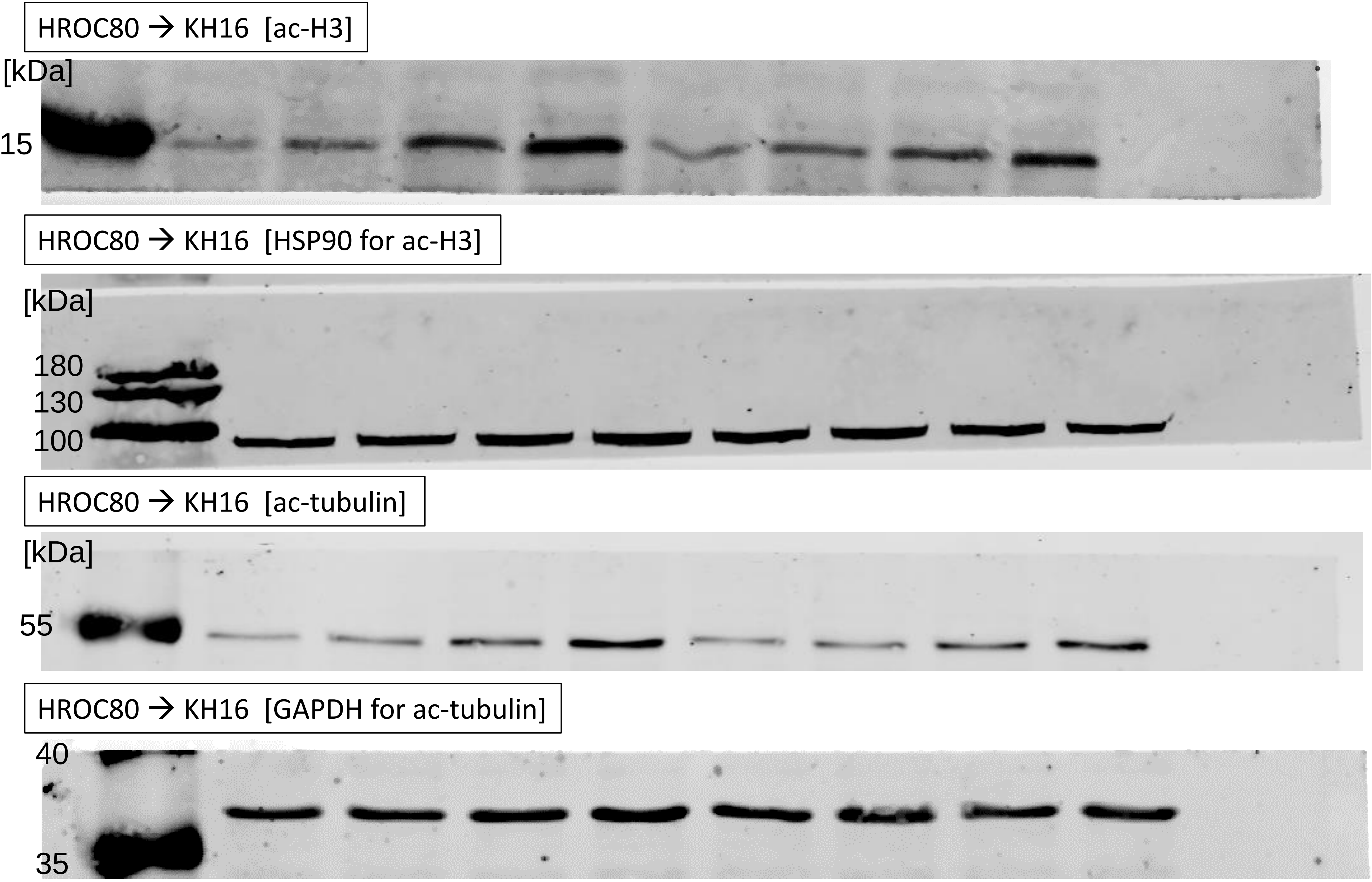
HROC80 → KH16 [ac-H3]
[kDa]
15
HROC80 → KH16 [HSP90 for ac-H3]
[kDa]
180
130
100
HROC80 → KH16 [ac-tubulin]
[kDa]
55
HROC80 → KH16 [GAPDH for ac-tubulin]
40
35

HROC80 → KH16 [ac-H3(K9)]

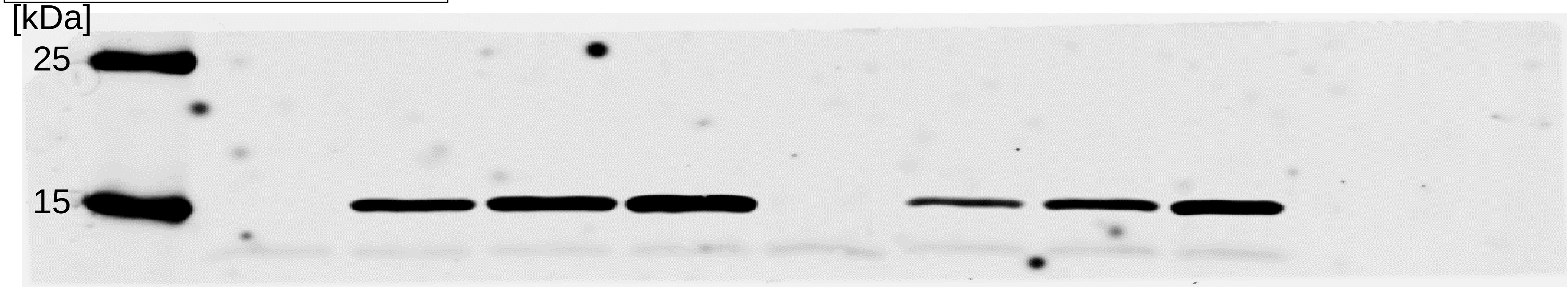

HROC80 → KH16 [H3]

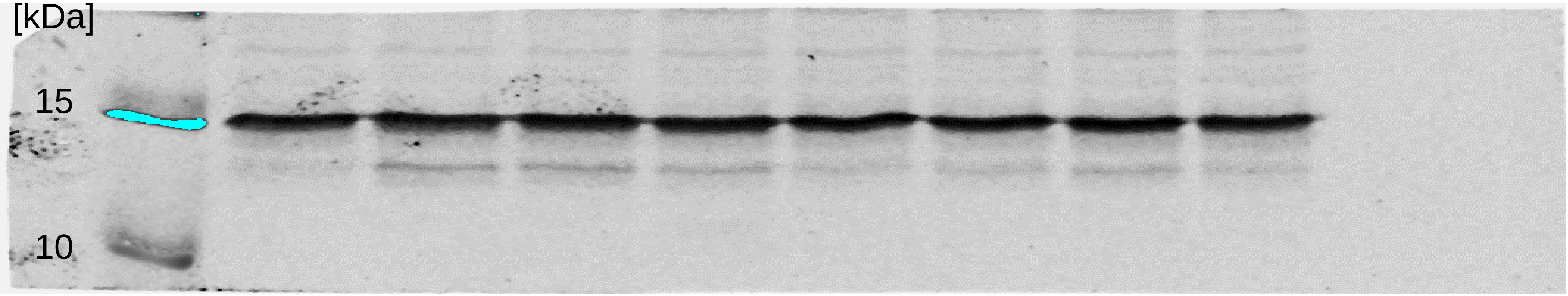

HROC80 → KH16 [HSP90 for ac-H3(K9)]

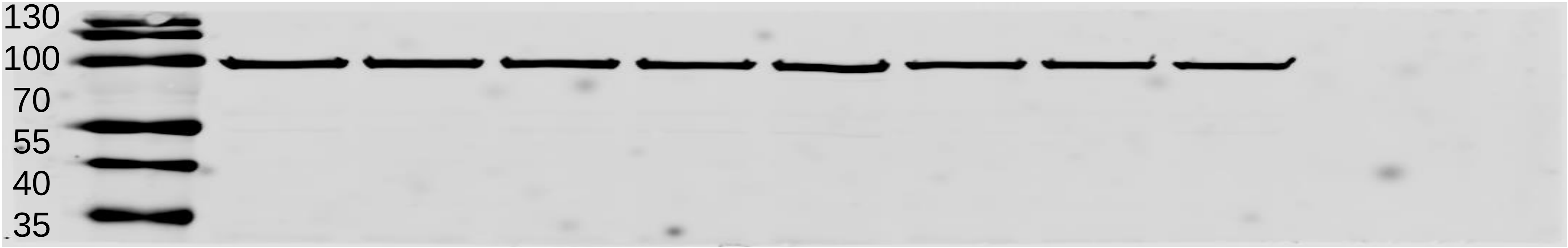

HROC80 → KH16 [ac-H3(K18)]

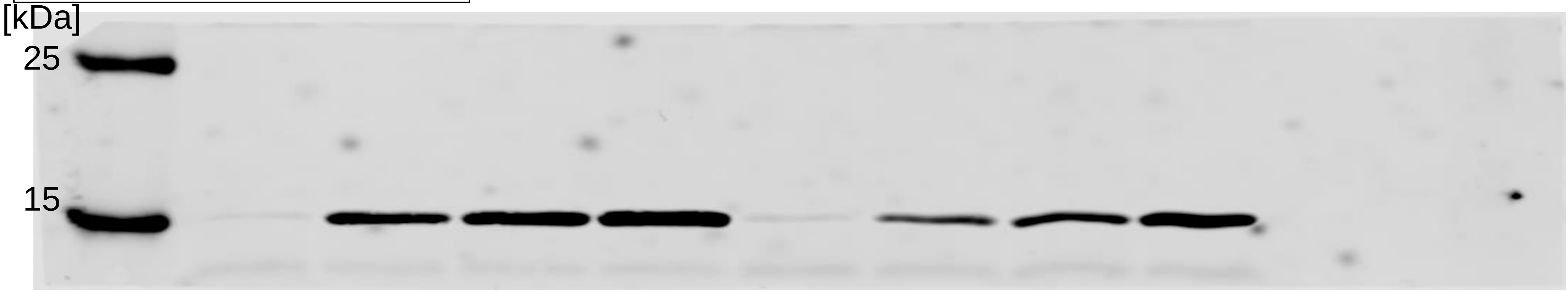

HROC80 → KH16 [HSP90 for ac-H3(K18)]

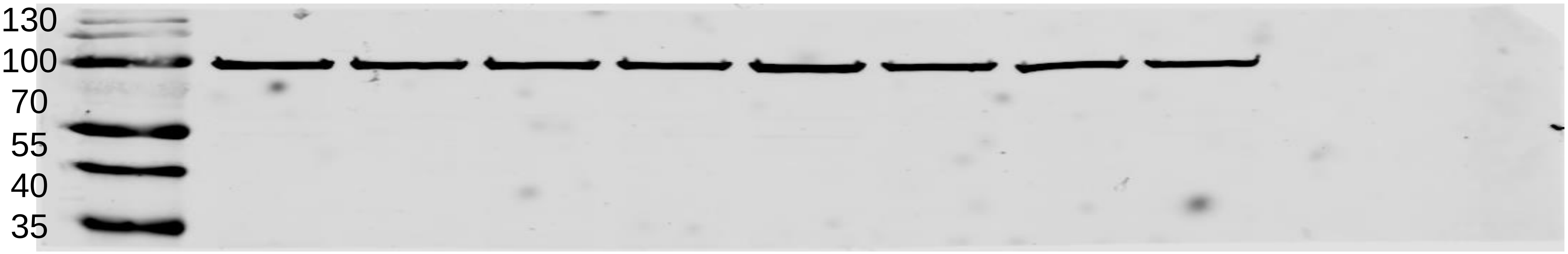

HROC80 → KH16 [ac-H3(K27)]

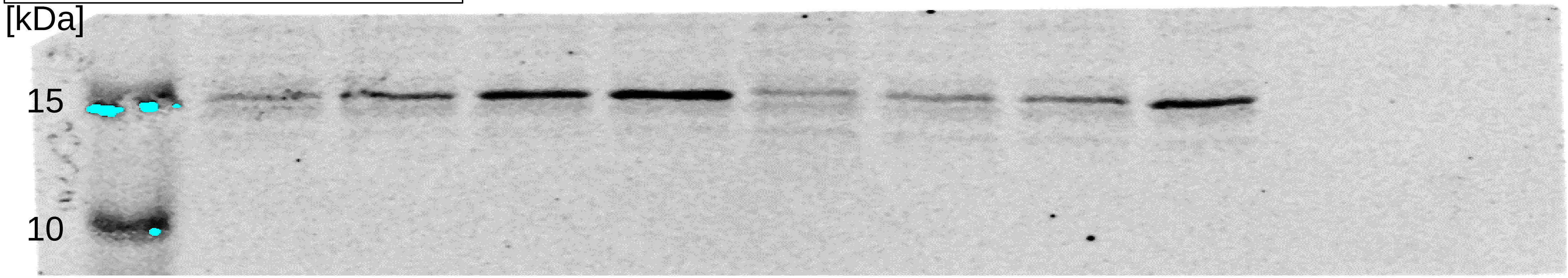

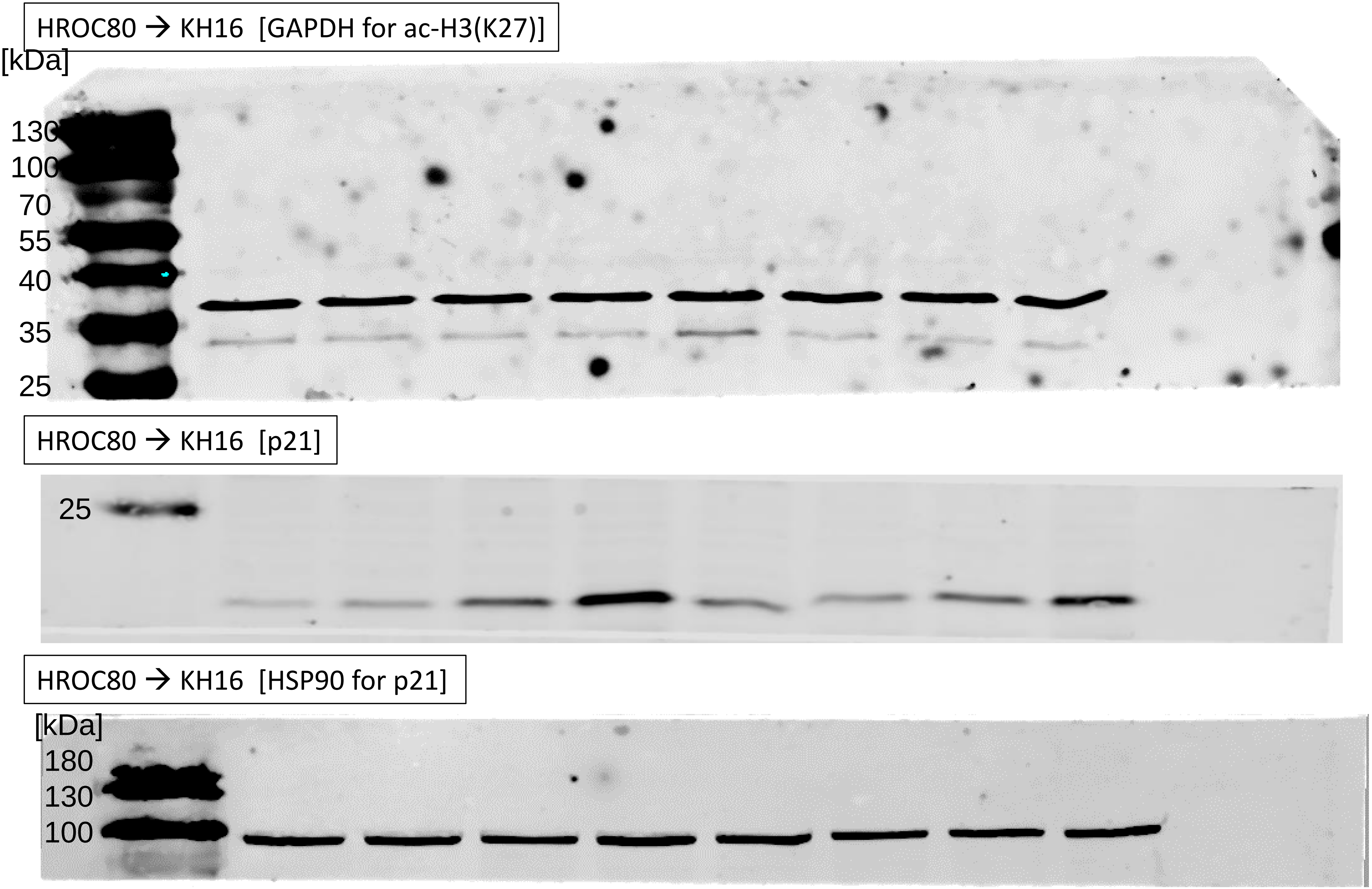
HROC80 → KH16 [GAPDH for ac-H3(K27)]
[kDa]
130
100
70
55
40
35
25
HROC80 → KH16 [p21]
25
HROC80 → KH16 [HSP90 for p21]
[kDa]
180
130
100

HCT116 → KH16 [ac-H3]

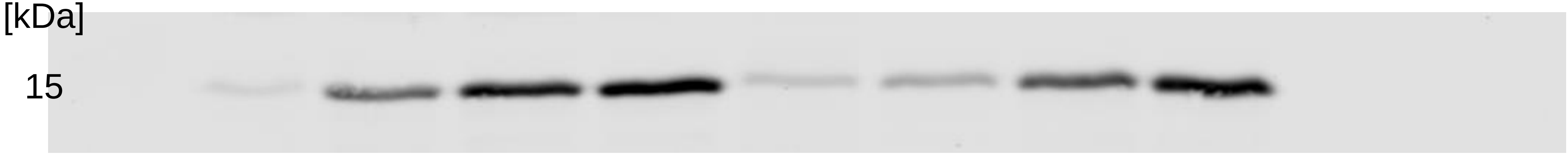

HCT116 → KH16 [HSP90 for ac-H3]

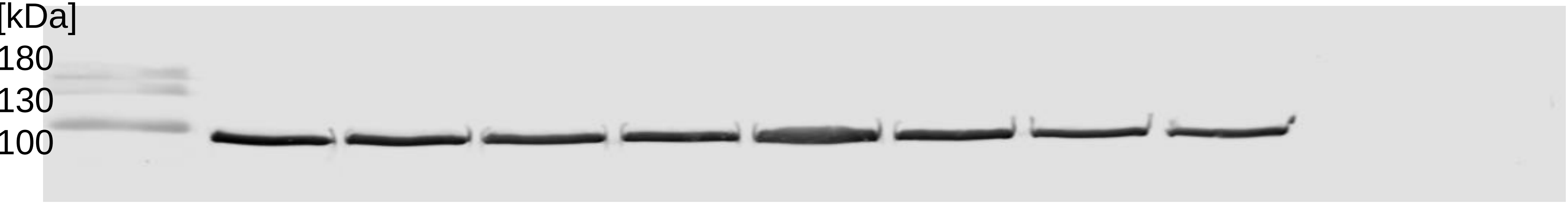

HCT116 → KH16 [ac-tubulin]

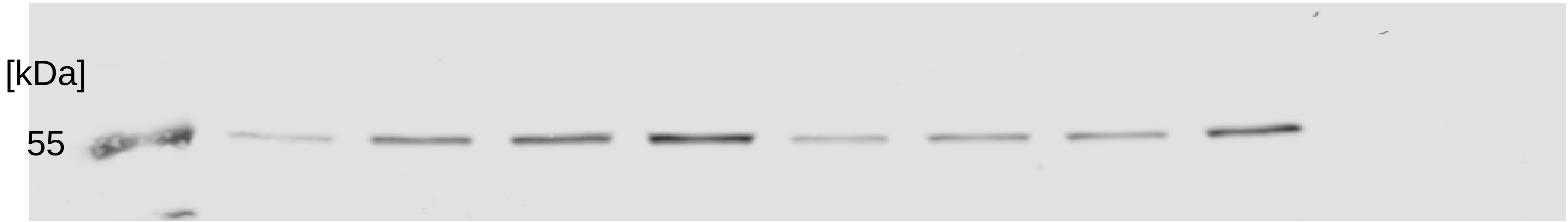

HCT116 → KH16 [GAPDH for ac-tubulin]

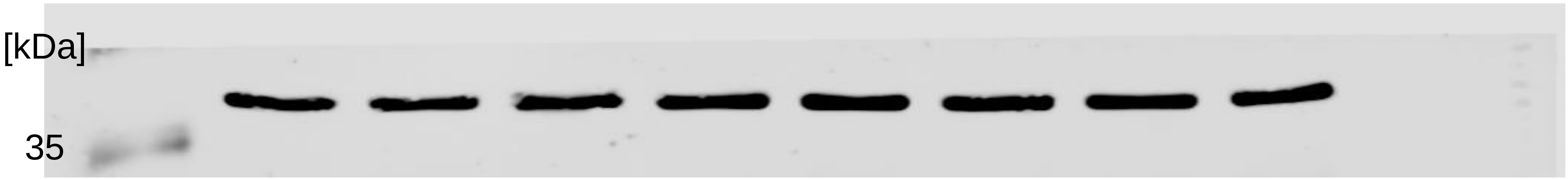

HCT116 → KH16 [ac-H3(K9)]

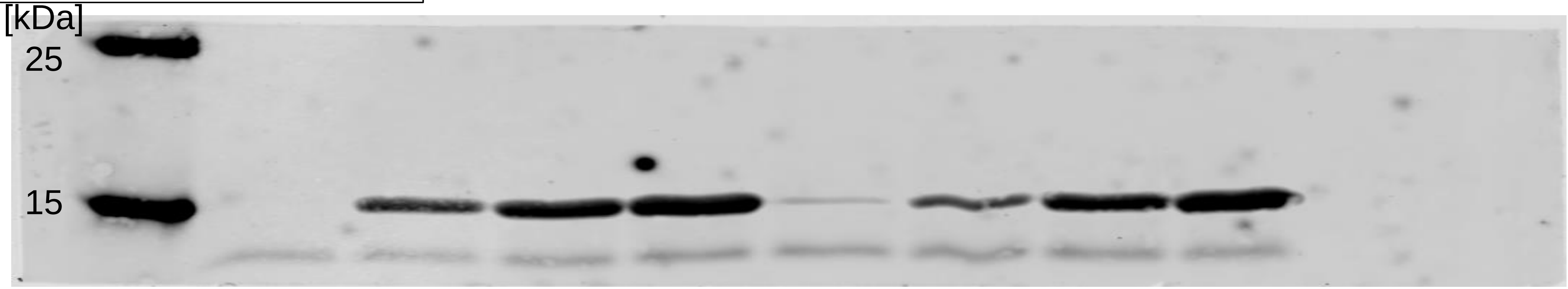

HCT116 → KH16 [H3]

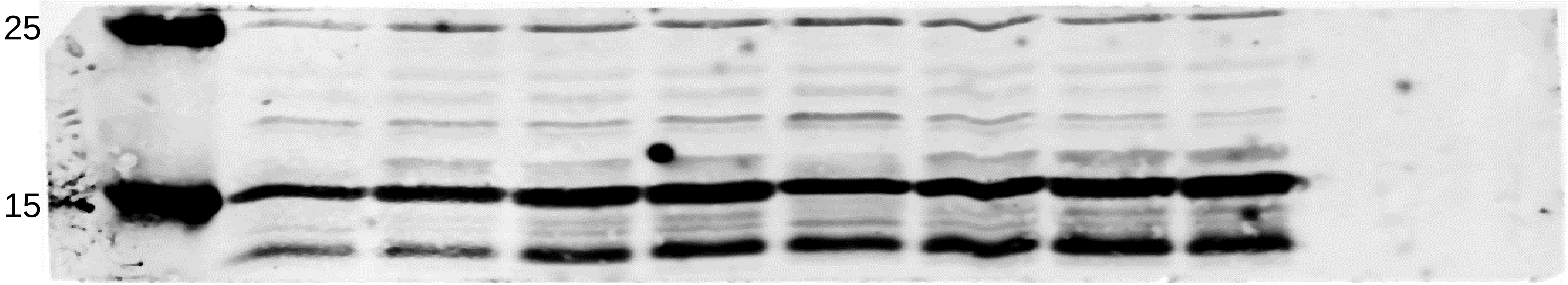

HCT116 → KH16 [HSP90 for ac-H3(K9)]

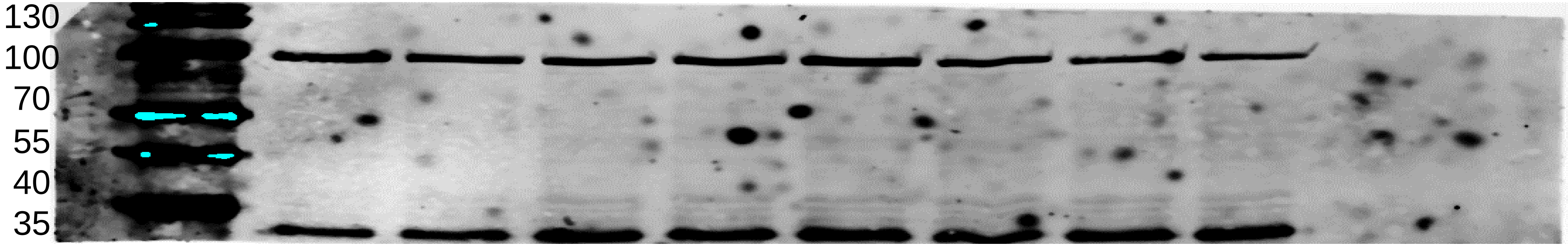

HCT116 → KH16 [ac-H3(K18)]

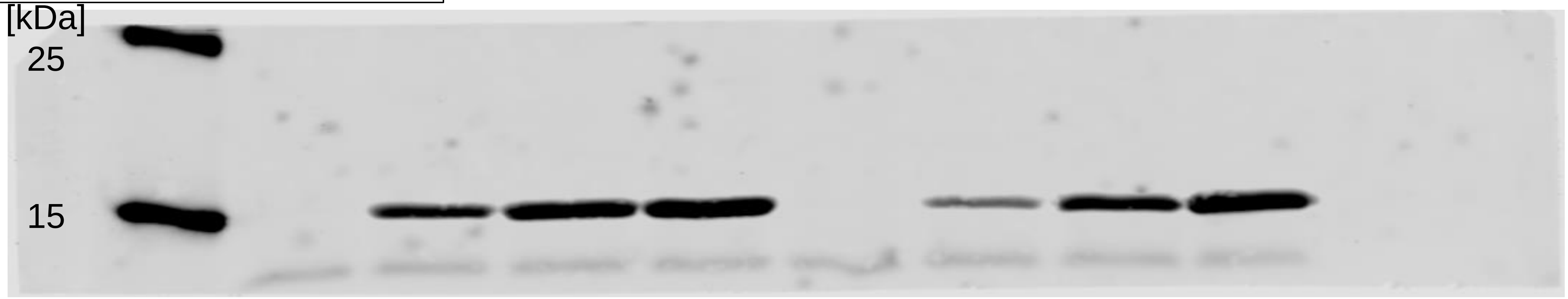

HCT116 → KH16 [HSP90 for ac-H3(K18)]

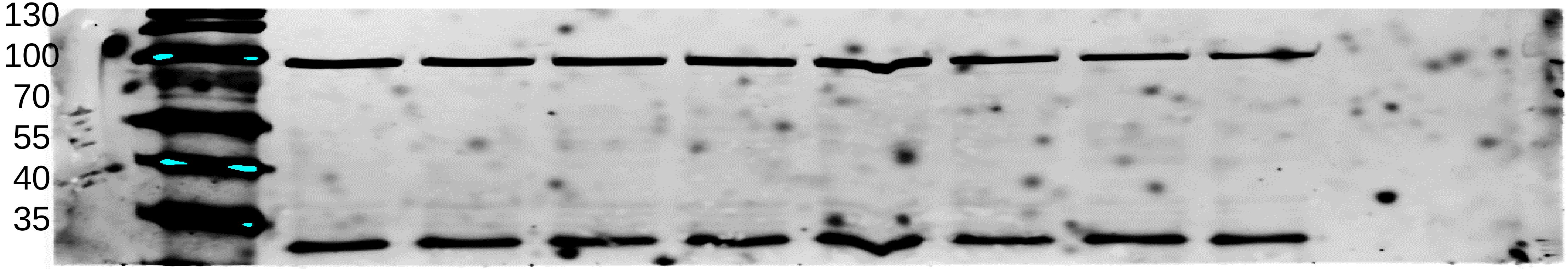

HCT116 → KH16 [ac-H3(K27)]

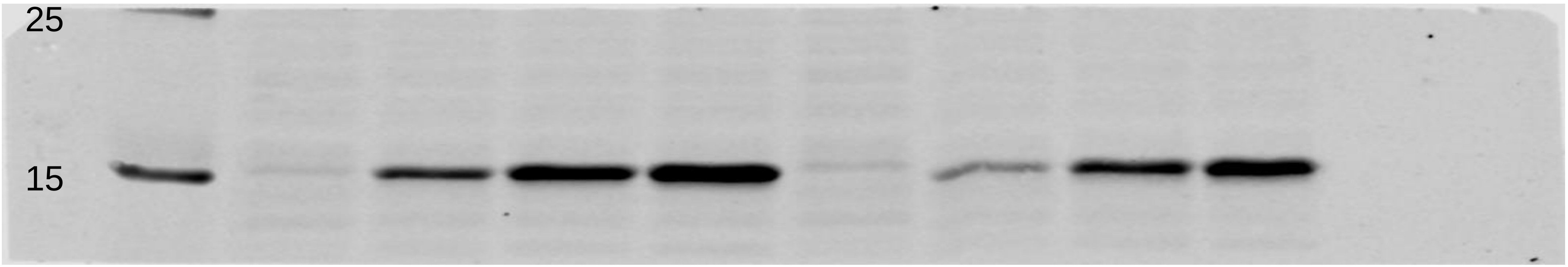

HCT116 → KH16 [GAPDH for ac-H3(K27)]

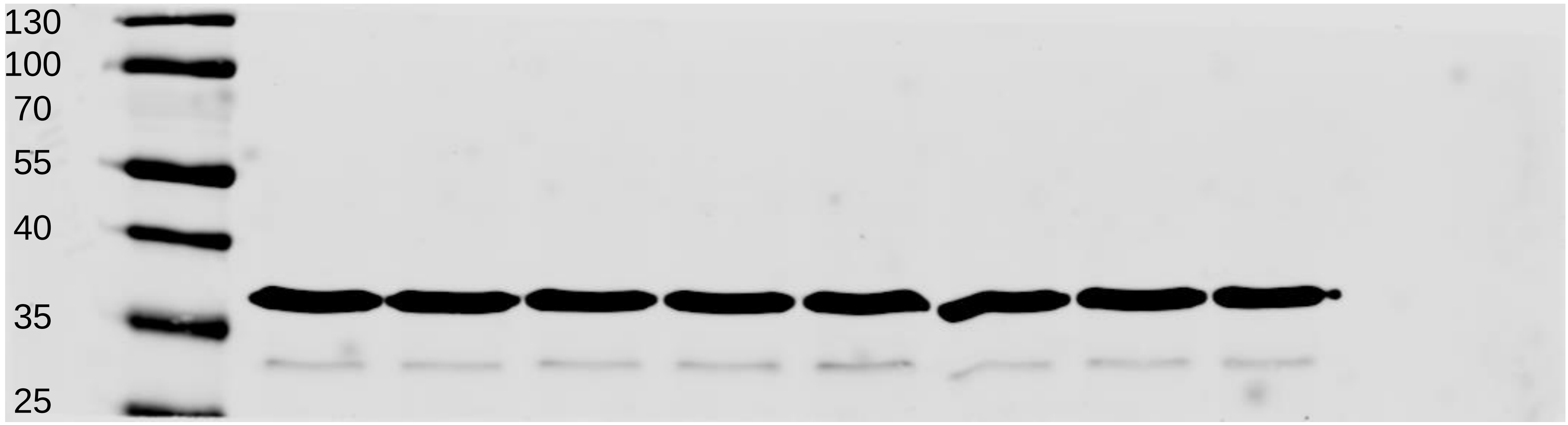

HCT116 → KH16 [p21]

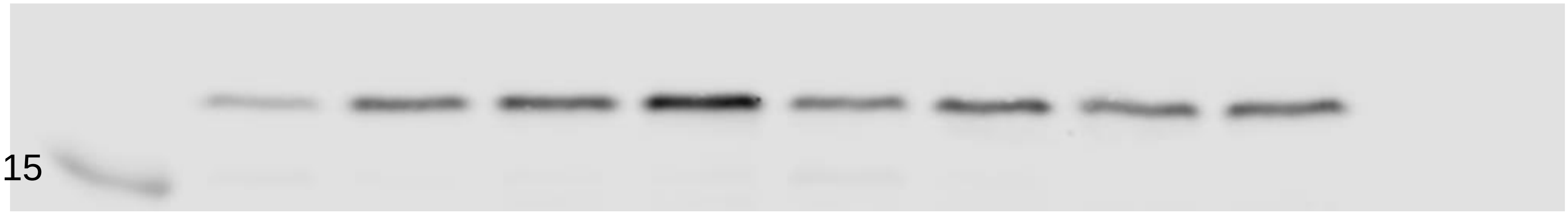

HCT116 → KH16 [HSP90 for p21]

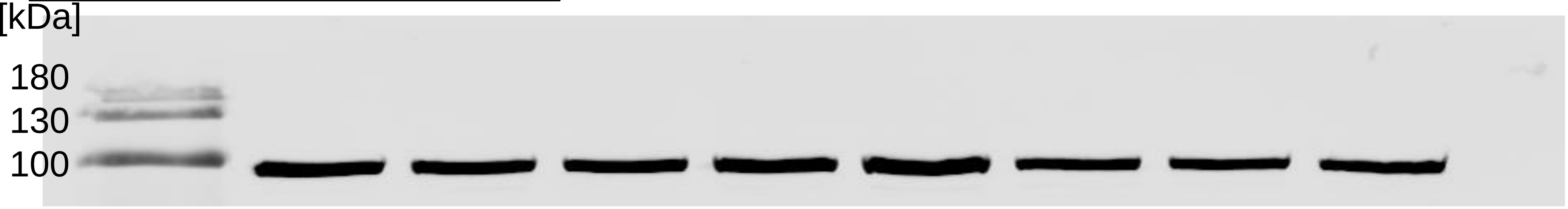

Original western blots of Figure 2

RPE1 → KH16 – 24h [ac-H3]

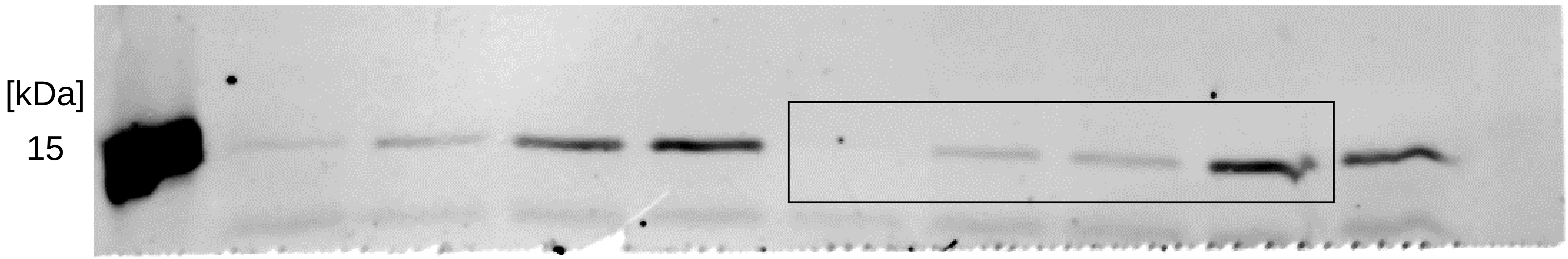

RPE1 → KH16 – 24h & 48h [HSP90 for ac-H3]

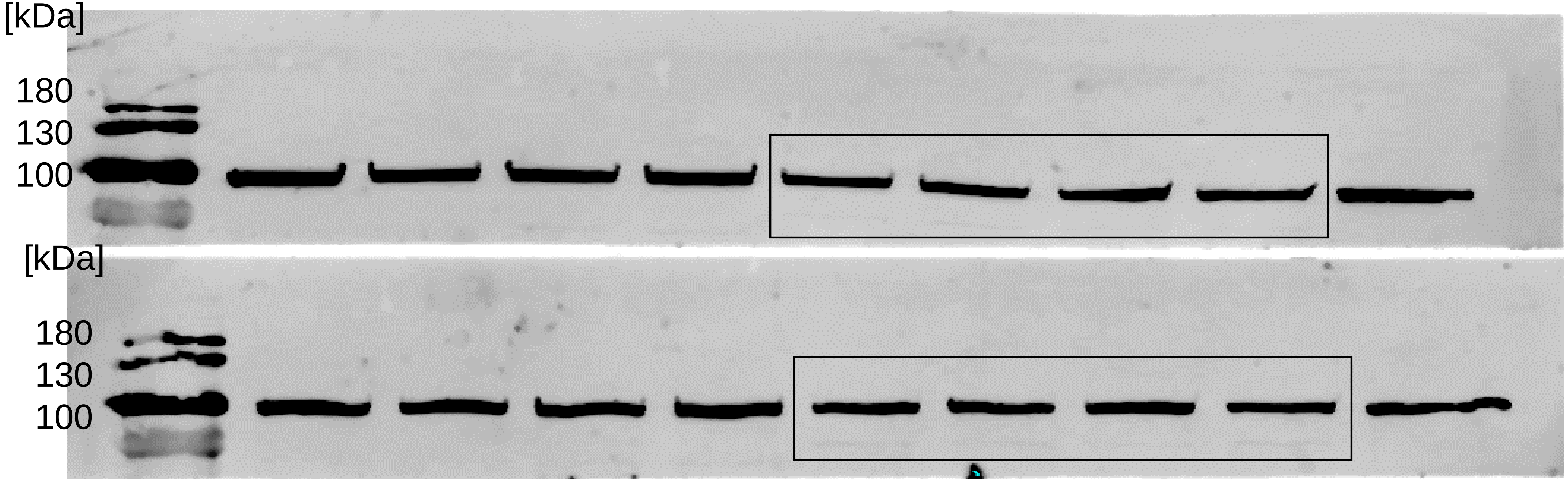

RPE1 → KH16 – 48h [ac-H3]

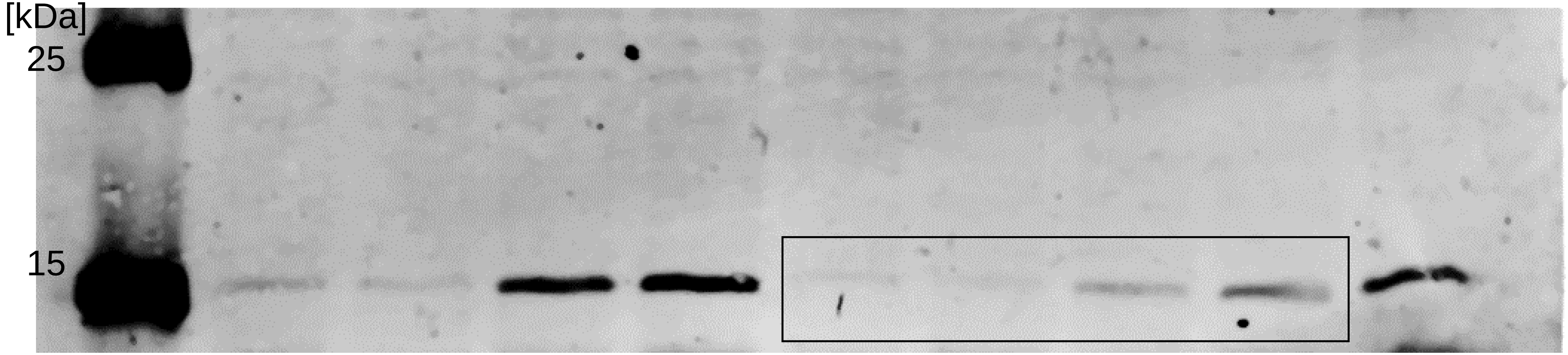

RPE1 → KH16 – 24h & 48h [HSP90 for ac-tubulin]

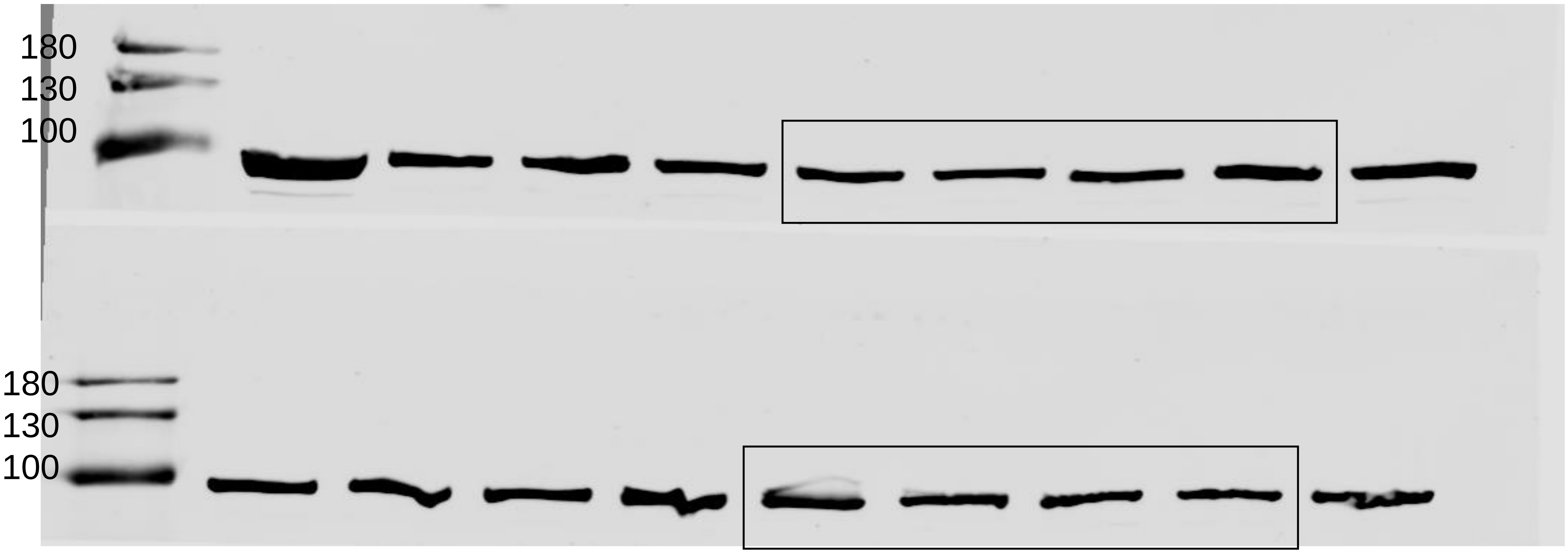

RPE1 → KH16 – 24h & 48h [ac-tubulin]

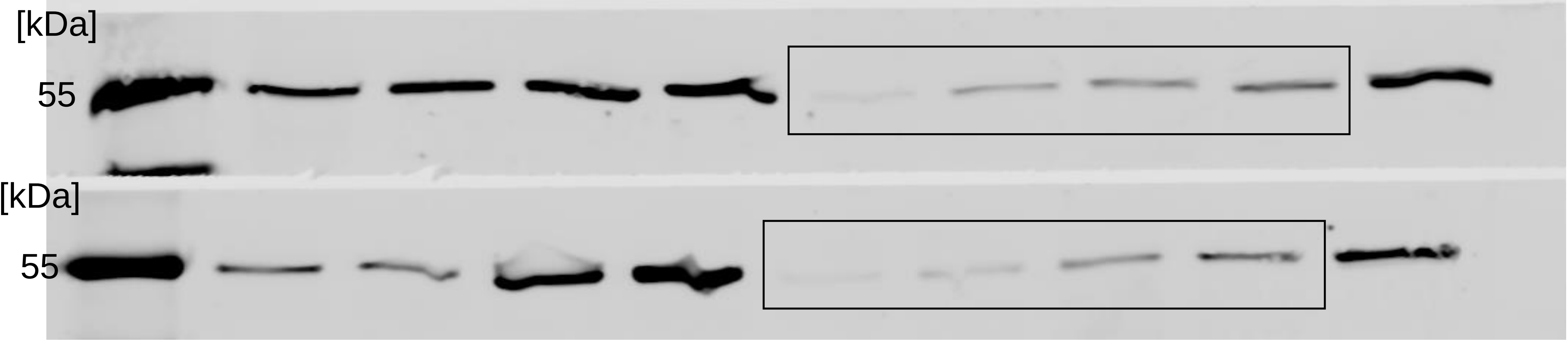

RPE1 → KH16 – 24h & 48h [H3]

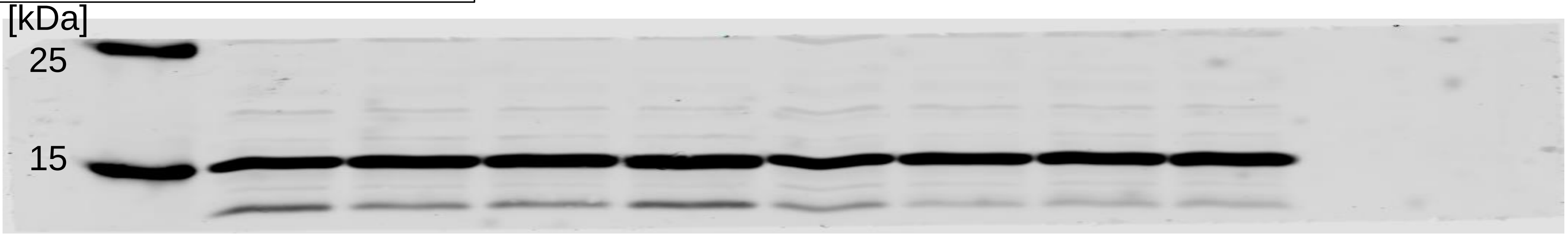

MIA PaCa-2 → KH16 [cl.PARP1]

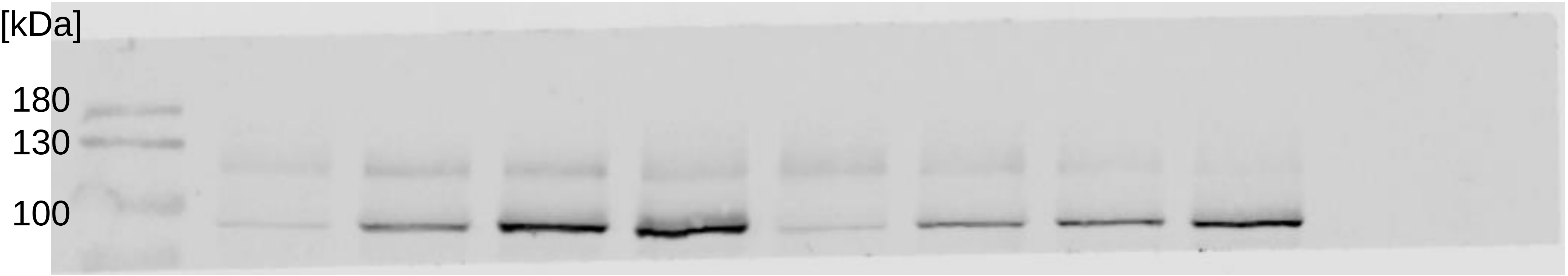

MIA PaCa-2 → KH16 [GAPDH for cl.PARP1]

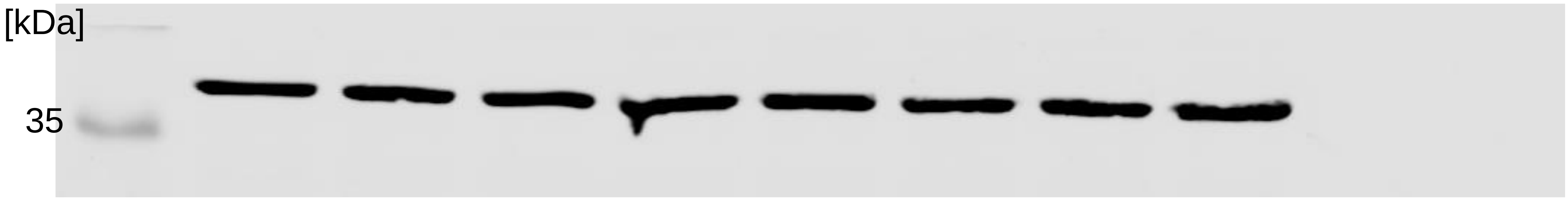

MIA PaCa-2 → KH16 [cl.caspase-3]

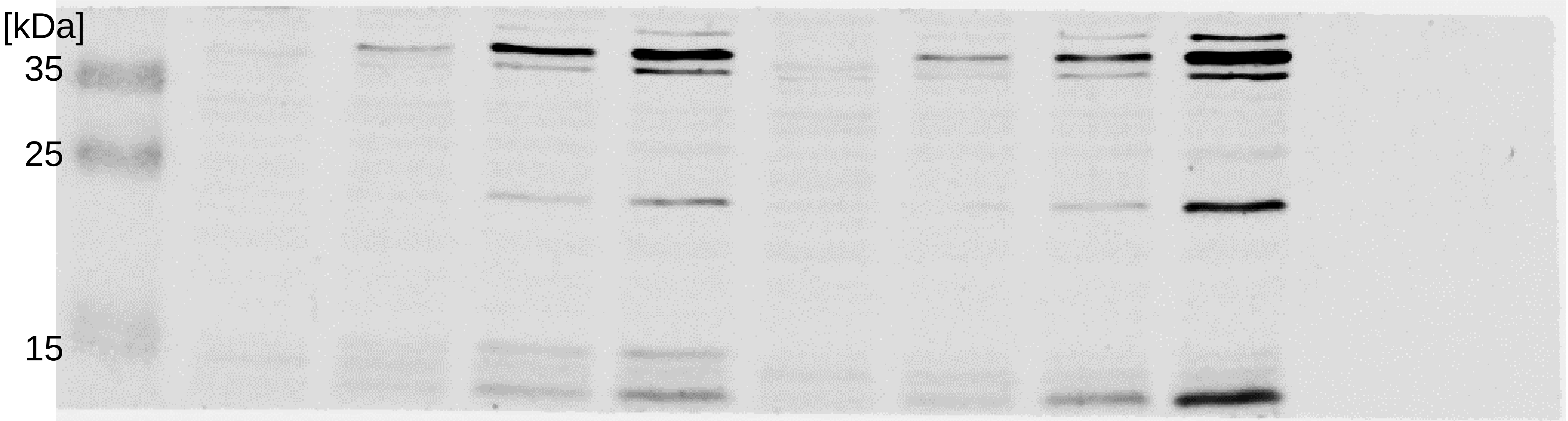

MIA PaCa-2 → KH16 [HSP90 for cl.caspase-3]

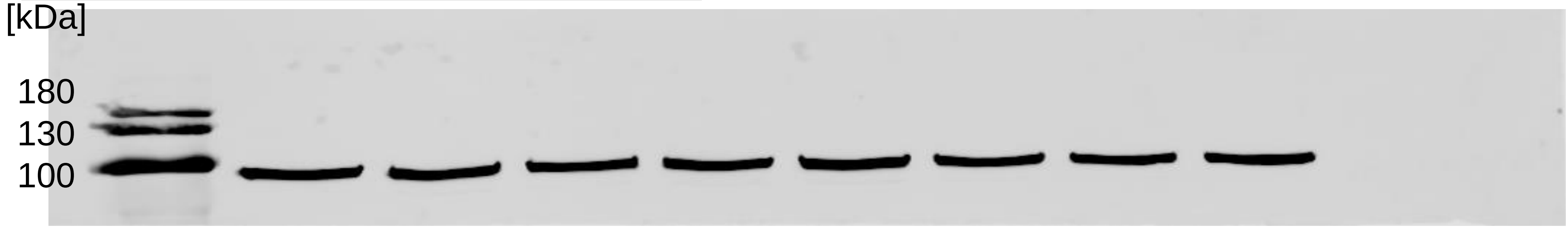

HROC80 → KH16 [cl.caspase-3]

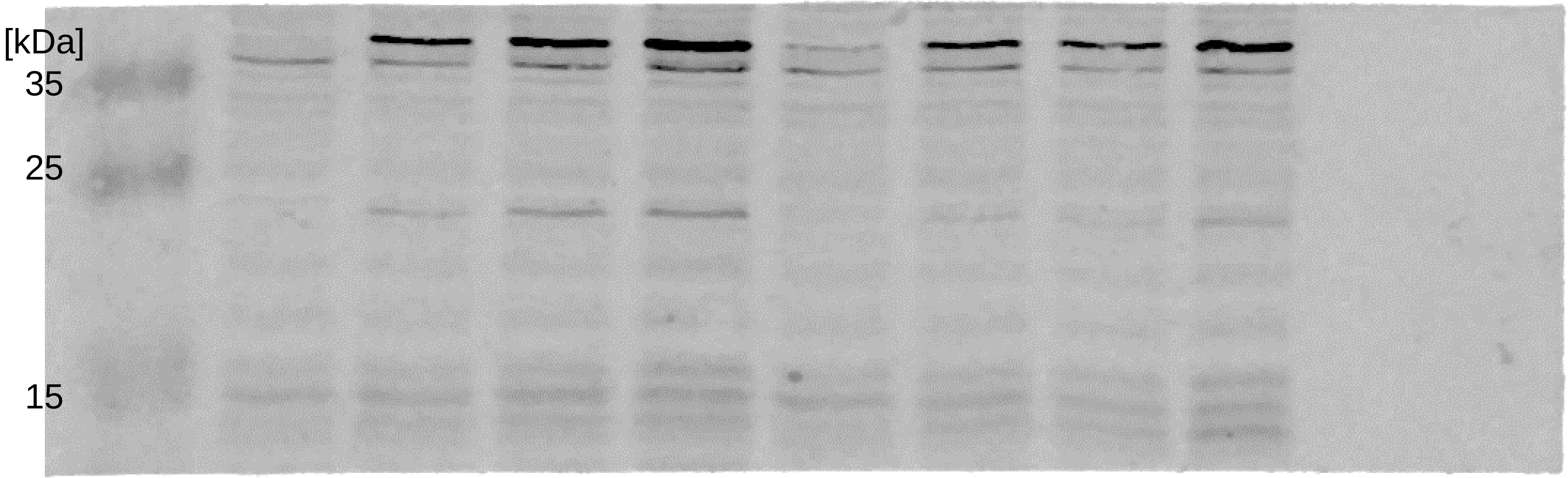

HROC80 → KH16 [HSP90 for cl.caspase-3]

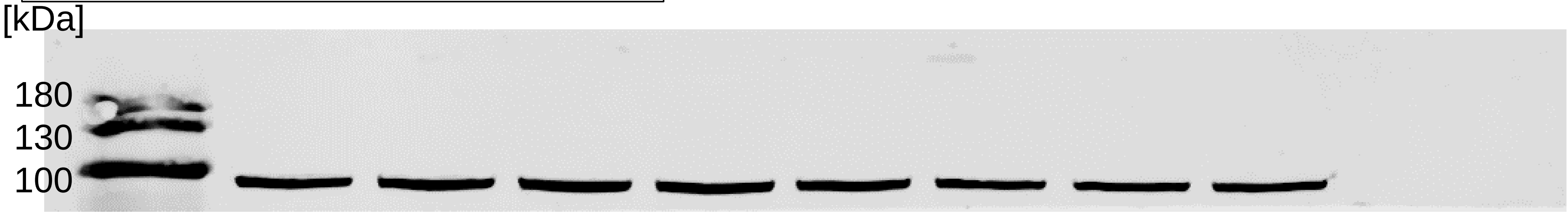

HCT116 → KH16 [cl.caspase-3]

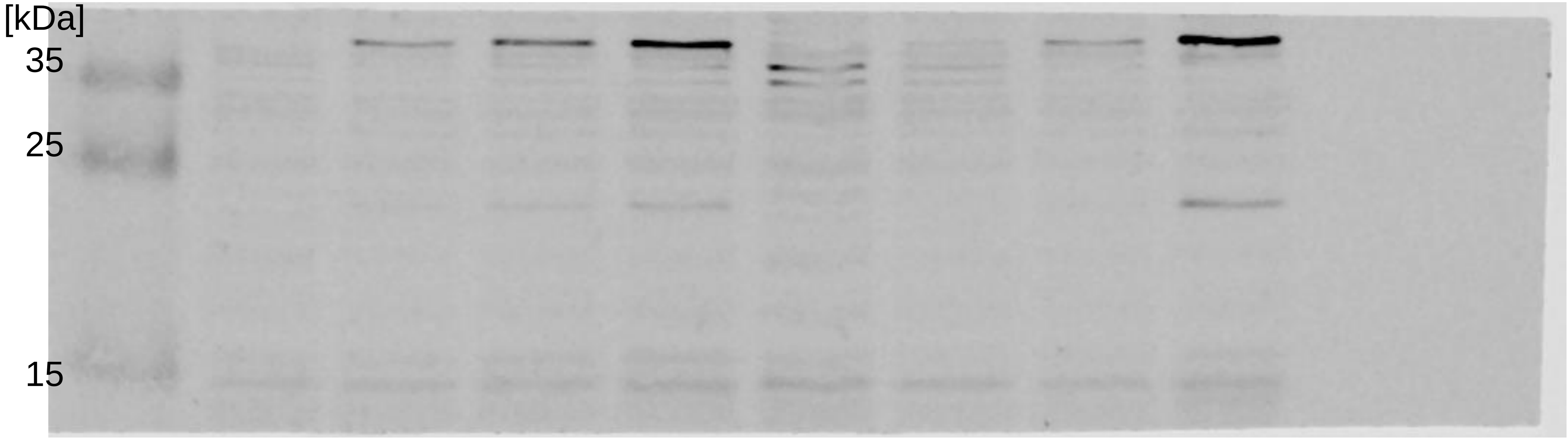

HCT116 → KH16 [HSP90 for cl.caspase-3]

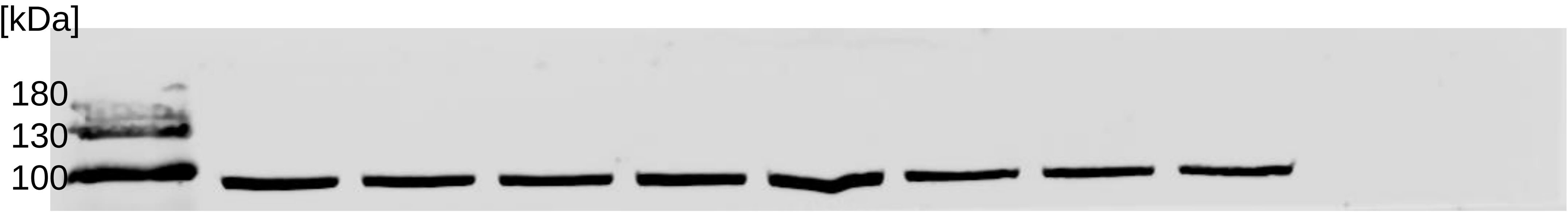

RPE1 vs MIA PaCa-2 → KH16 – 24h [cl.PARP1]

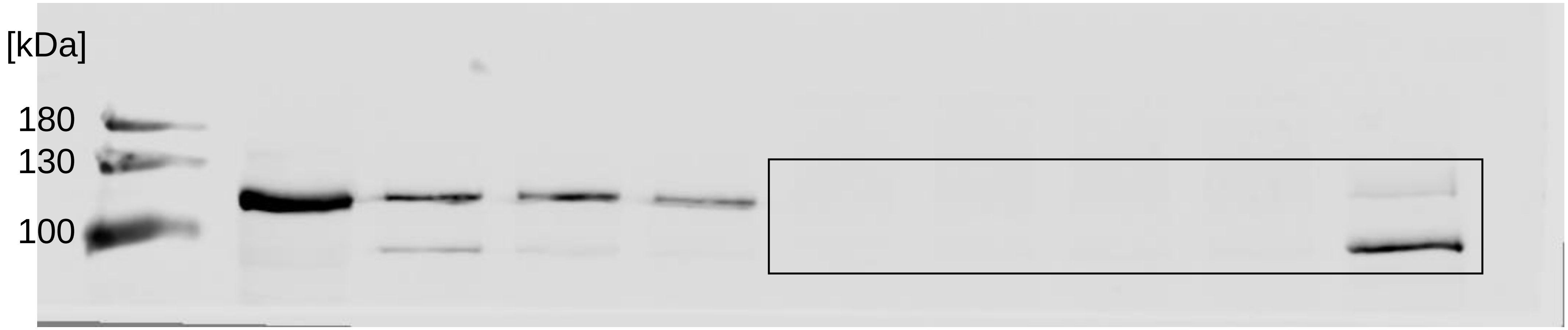

RPE1 vs MIA PaCa-2 → KH16 – 48h [cl.PARP1]

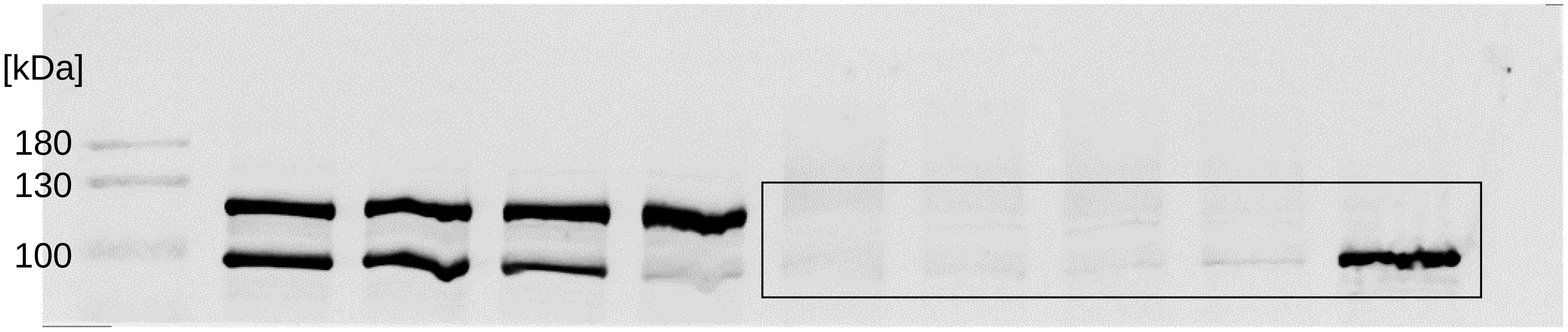

RPE1 vs MIA PaCa-2 → KH16 – 24h & 48h [HSP90 for cl.PARP1]

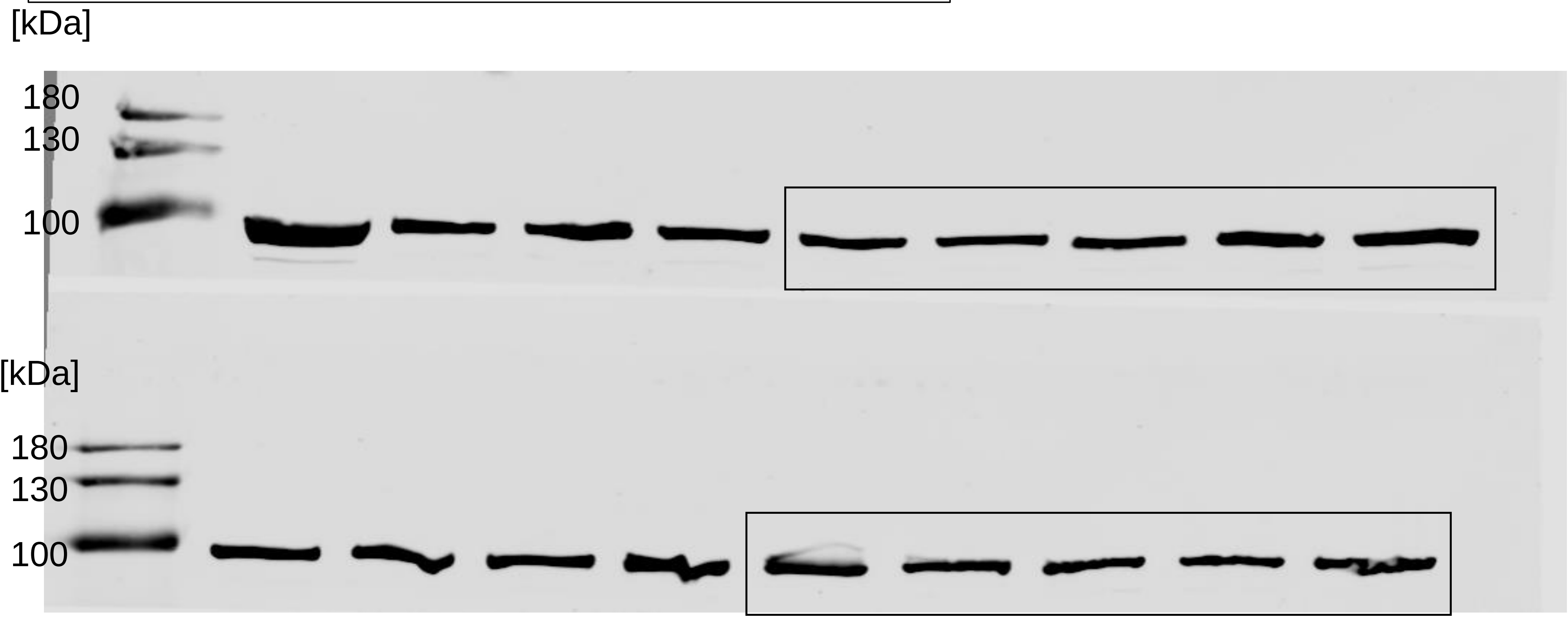

RPE1 vs MIA PaCa-2 → KH16 – 24h [cl.caspase-3]

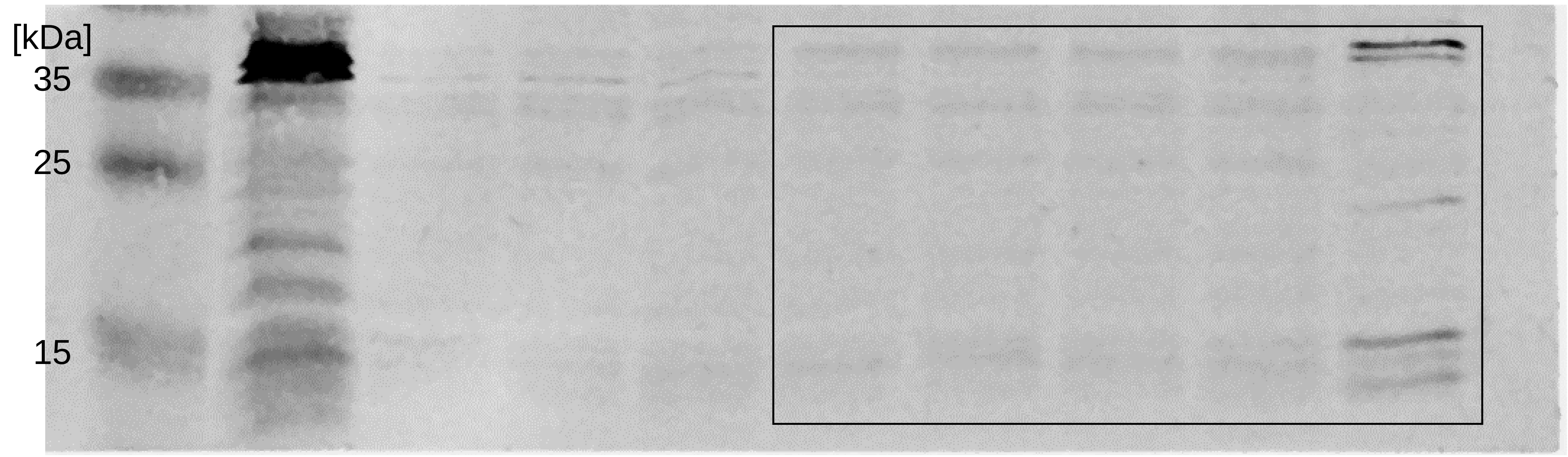

RPE1 vs MIA PaCa-2 → KH16 – 24h [HSP90 for cl.caspase-3]

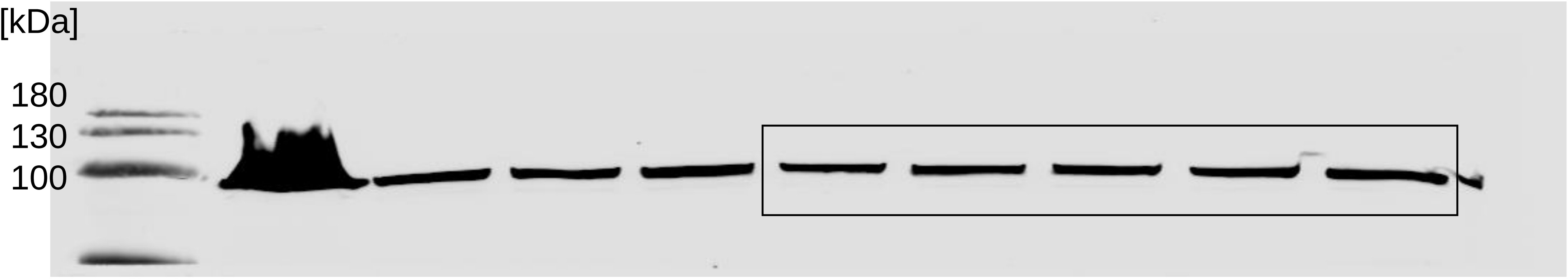

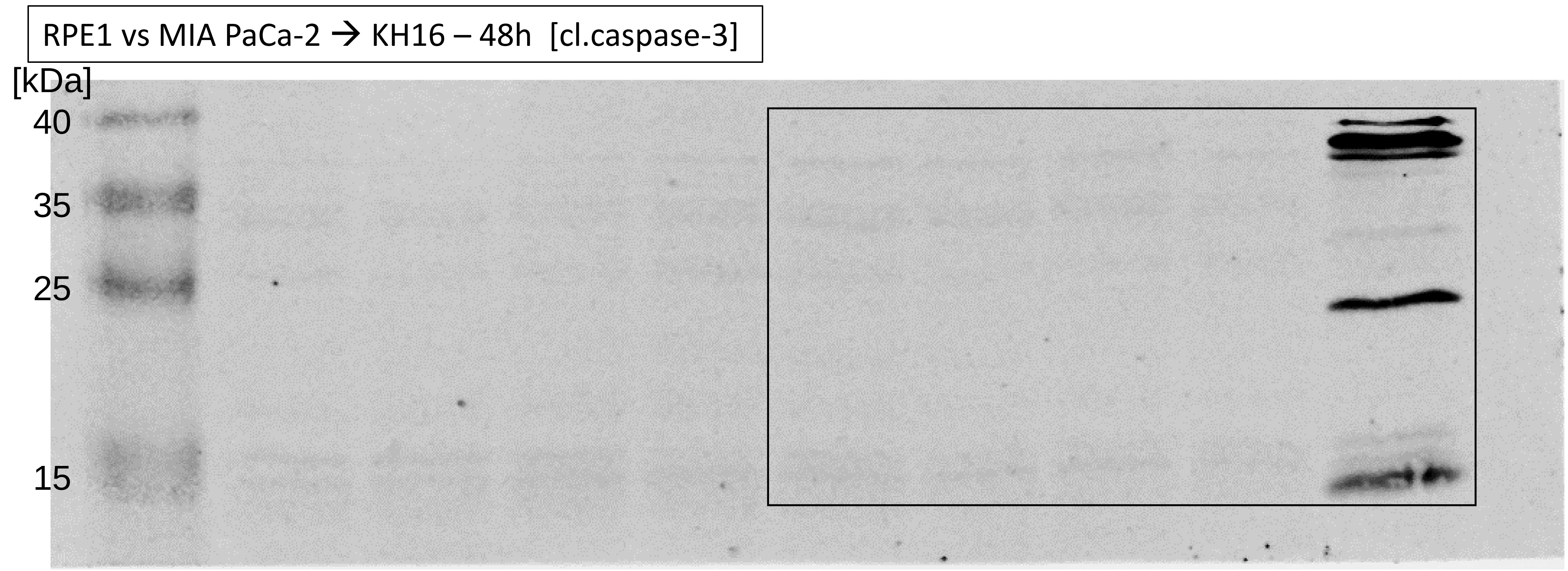
RPE1 vs MIA PaCa-2 → KH16 – 48h [cl.caspase-3]
[kDa]
40
35
25
15

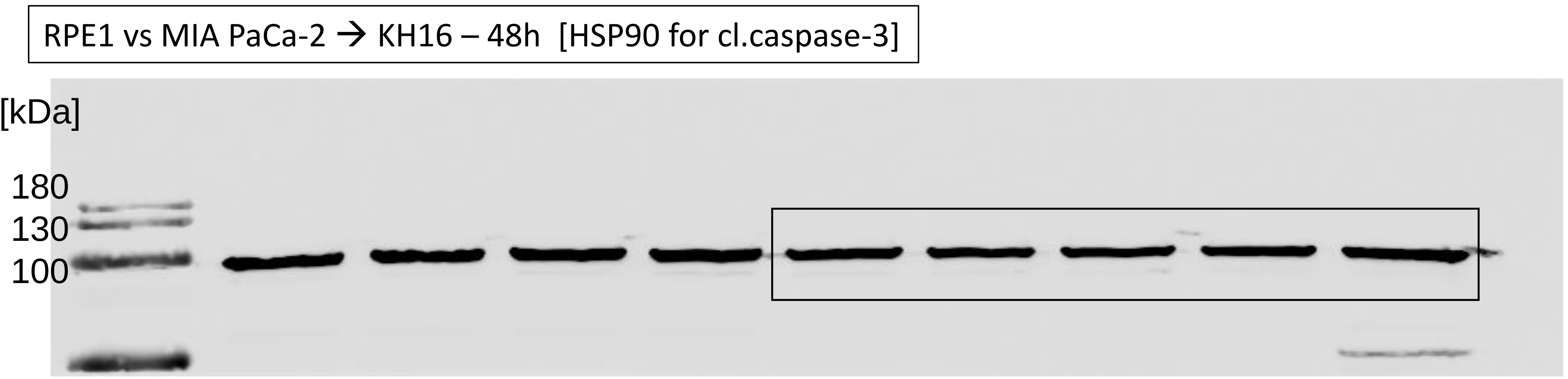
RPE1 vs MIA PaCa-2 → KH16 – 48h [HSP90 for cl.caspase-3]
[kDa]
180
130
100

Original western blots of Figure 3

MIA PaCa-2 → KH16 & Z-VAD-FMK [cl.caspase-3]

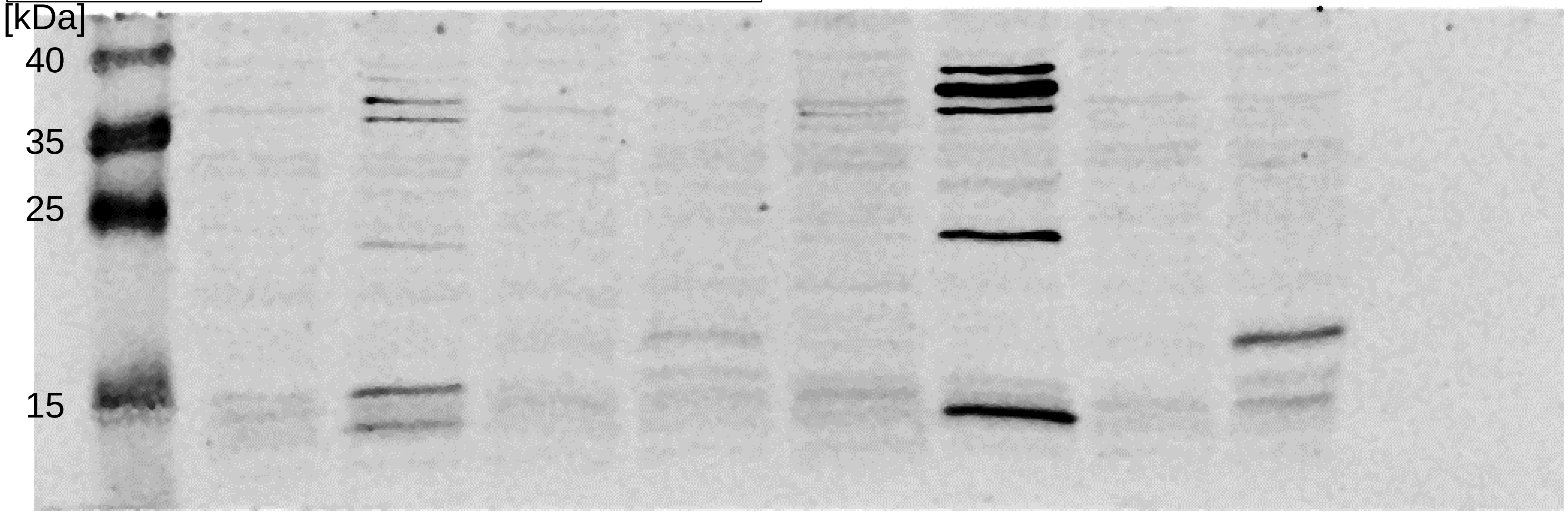

MIA PaCa-2 → KH16 & Z-VAD-FMK [HSP90 for cl.caspase-3]

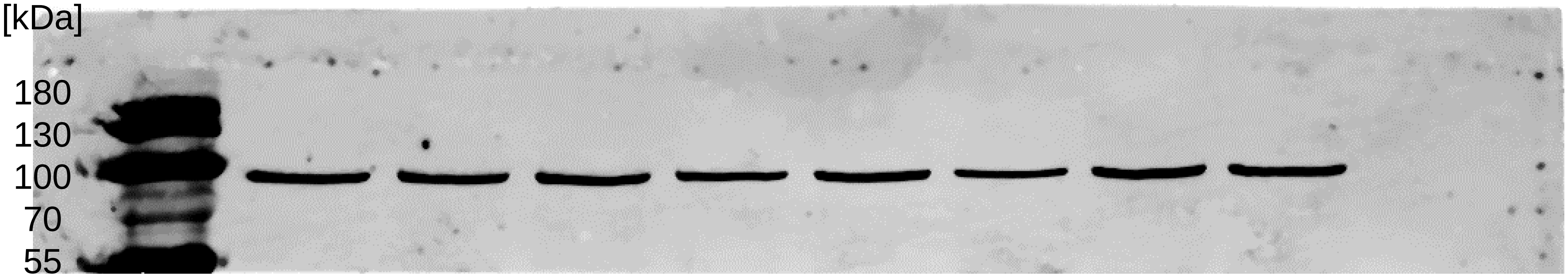

MIA PaCa-2 → KH16 & Z-VAD-FMK [cl.PARP1]

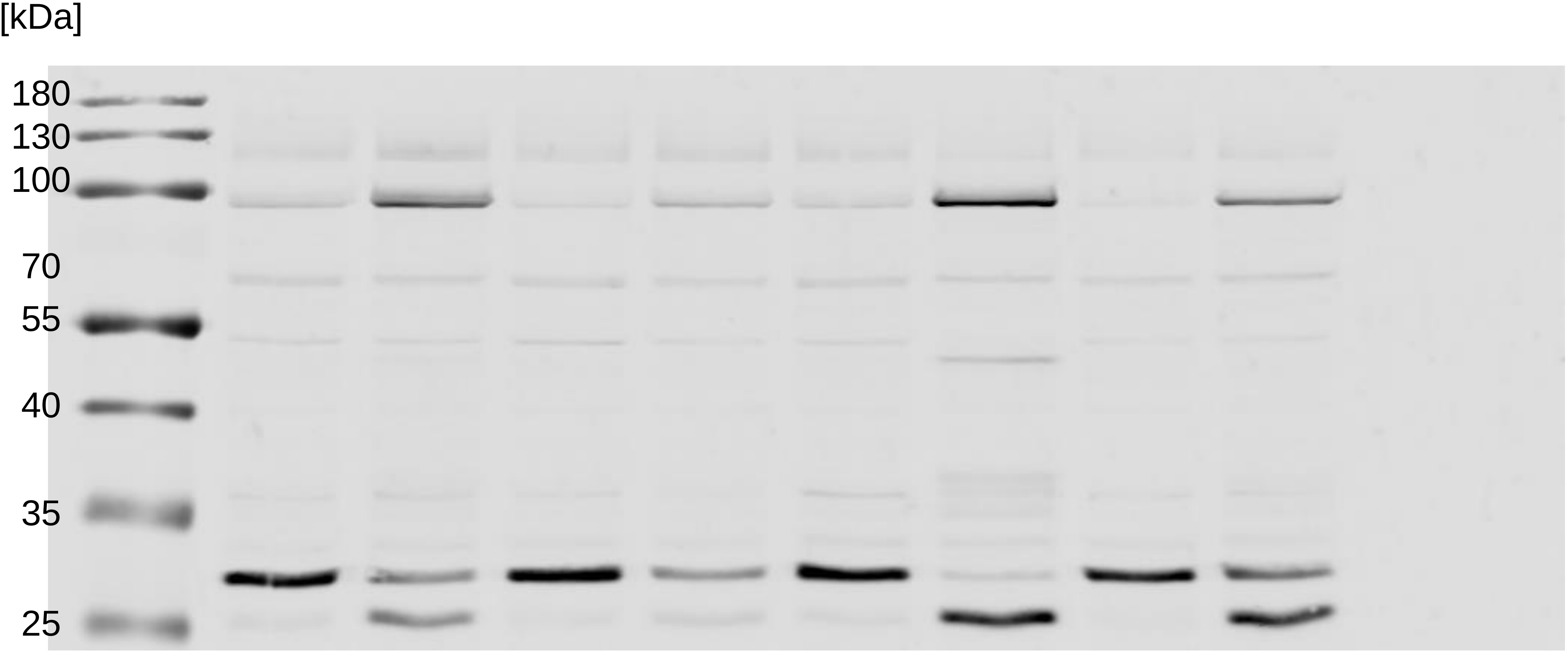

MIA PaCa-2 → KH16 & Z-VAD-FMK [HSP90 for cl.PARP1]

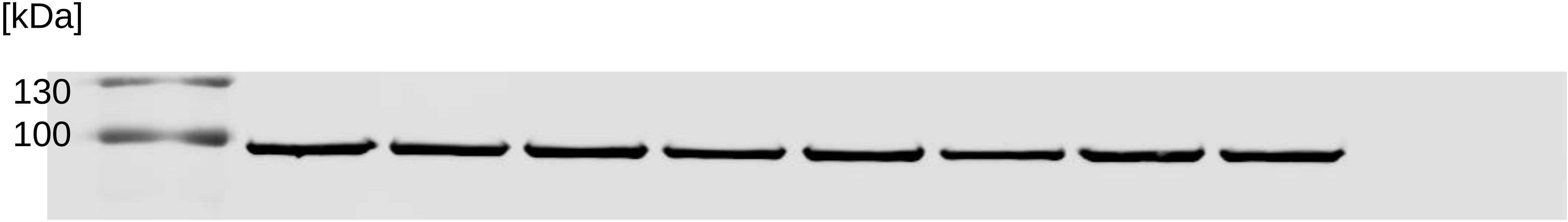

MIA PaCa-2 → KH16 [BAX]

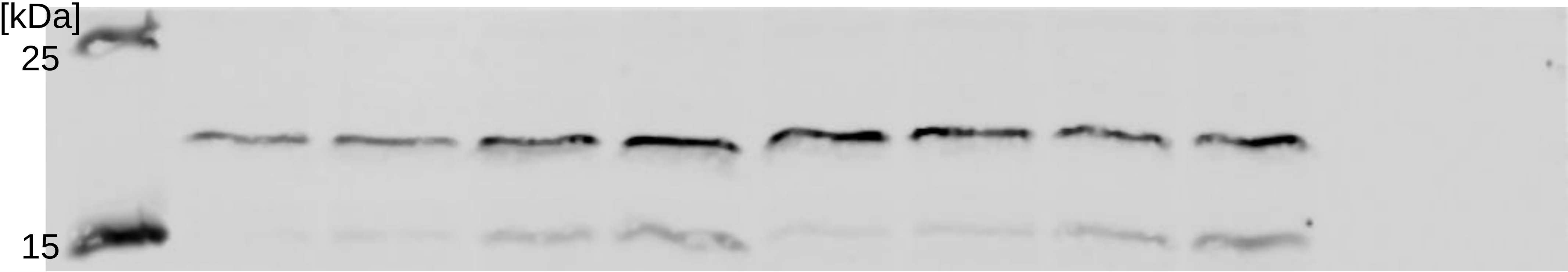

MIA PaCa-2 → KH16 [NOXA]

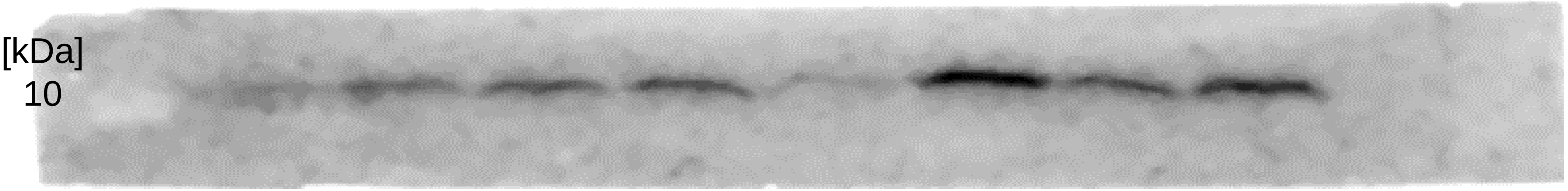

MIA PaCa-2 → KH16 [GAPDH for BAX & NOXA]

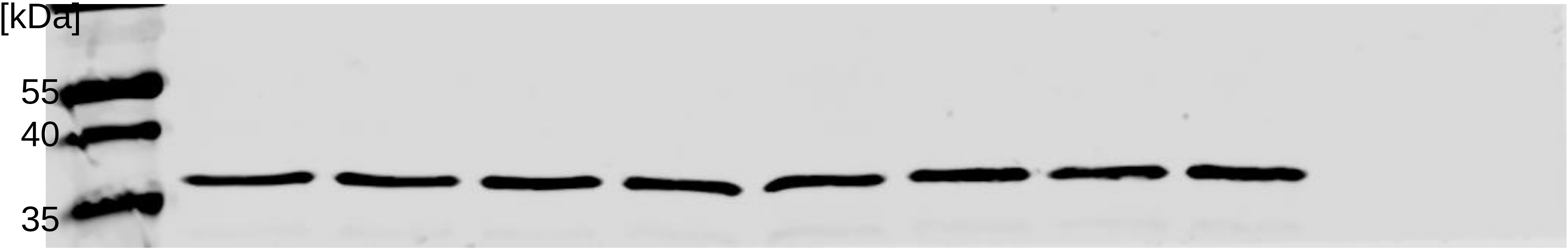

MIA PaCa-2 → KH16 [BAK]

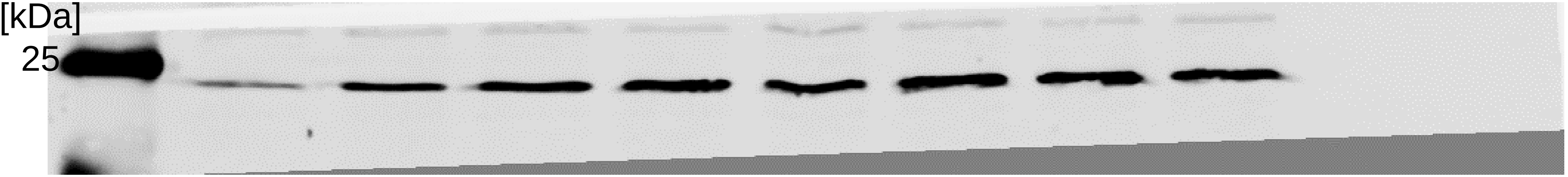

MIA PaCa-2 → KH16 [GAPDH for BAK]

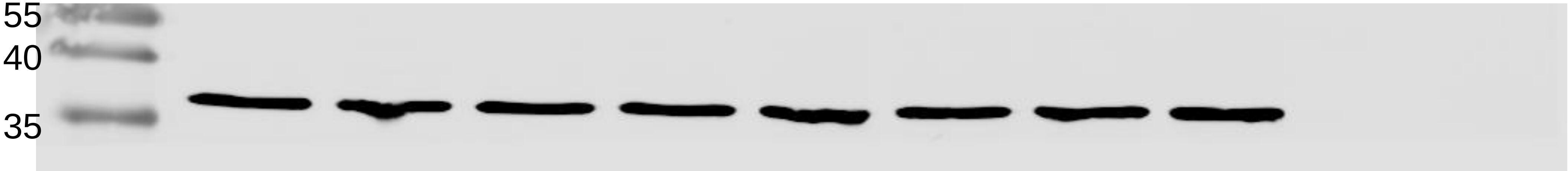

MIA PaCa-2 → KH16 [BIM]

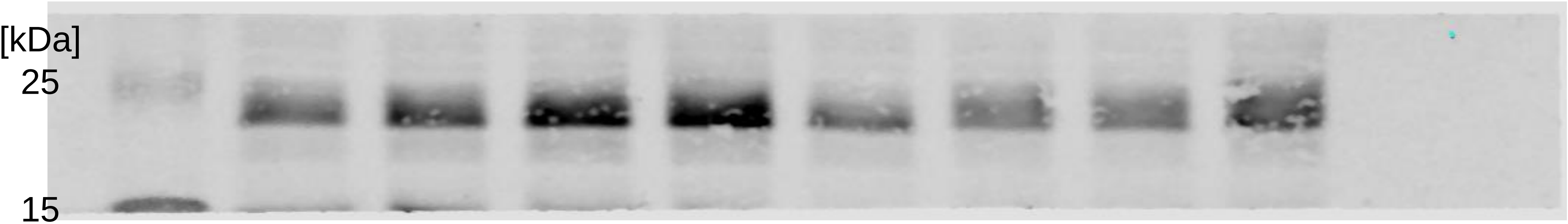

MIA PaCa-2 → KH16 [vinculin for BIM]

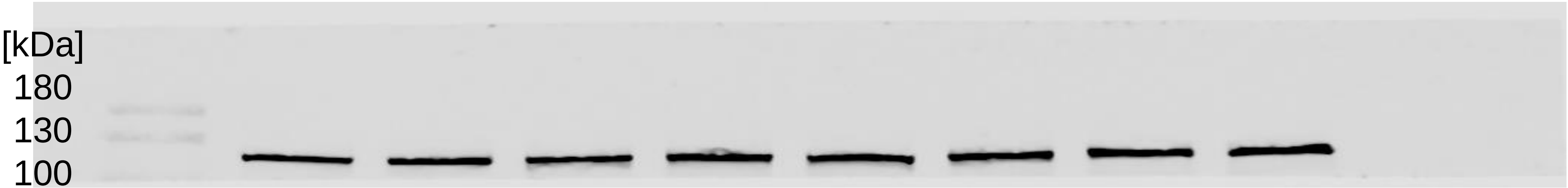

MIA PaCa-2 → KH16 [BID]

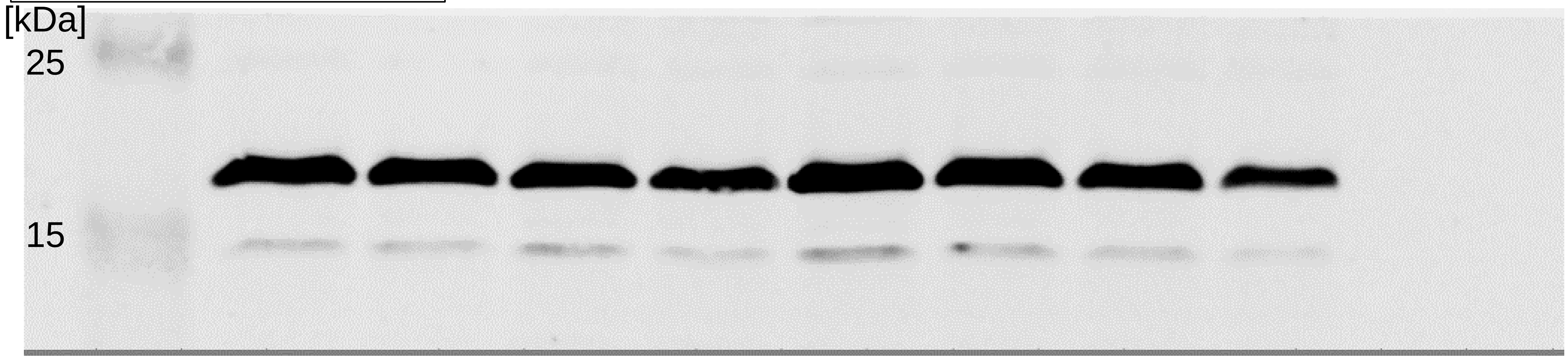

MIA PaCa-2 → KH16 [MCL-1]

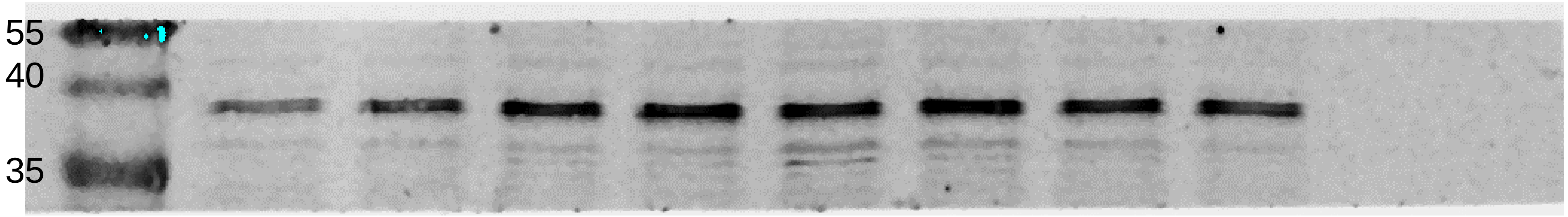

MIA PaCa-2 → KH16 [HSP90 for BID & MCL-1]

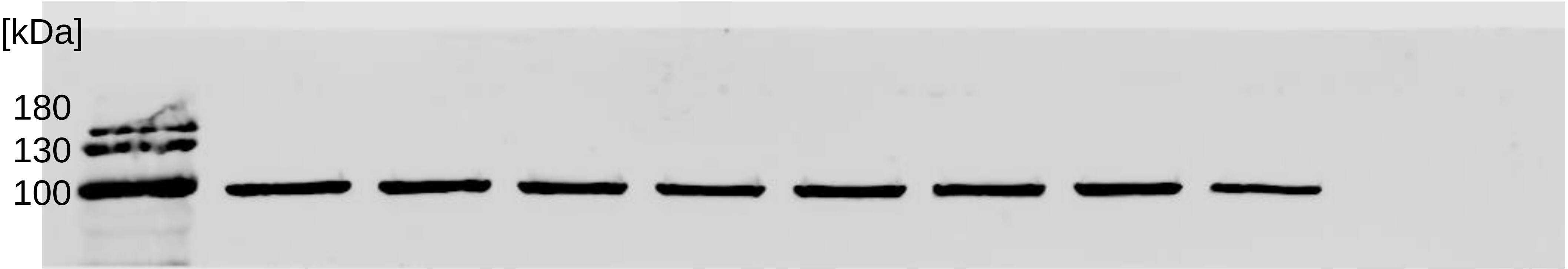

MIA PaCa-2 → KH16 [BCL-XL]

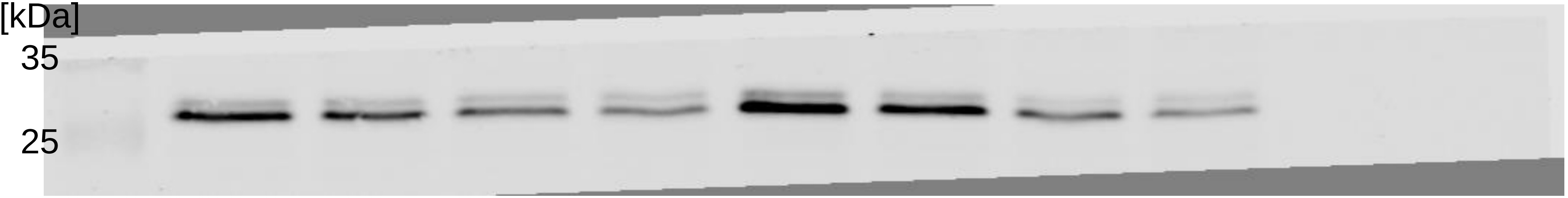

MIA PaCa-2 → KH16 [HSP90 for BCL-XL]

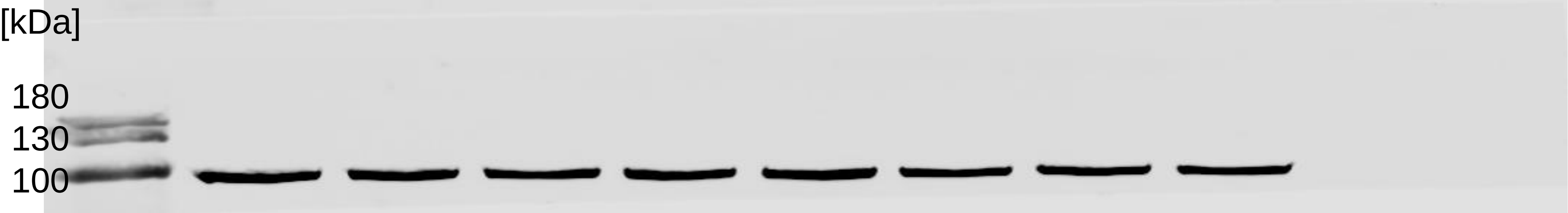

MIA PaCa-2 → KH16 [survivin]

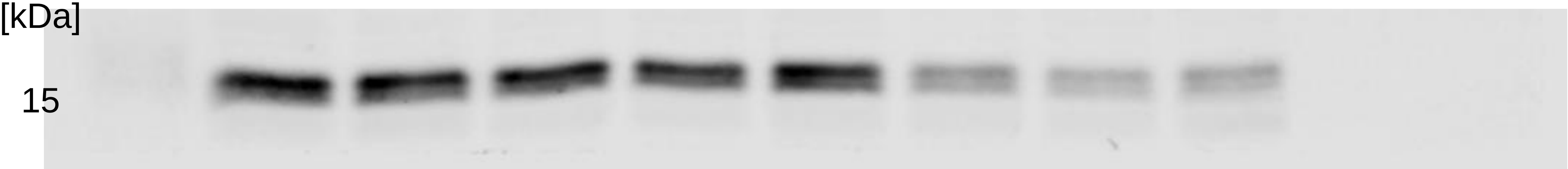

MIA PaCa-2 → KH16 [HSP90 for survivin]

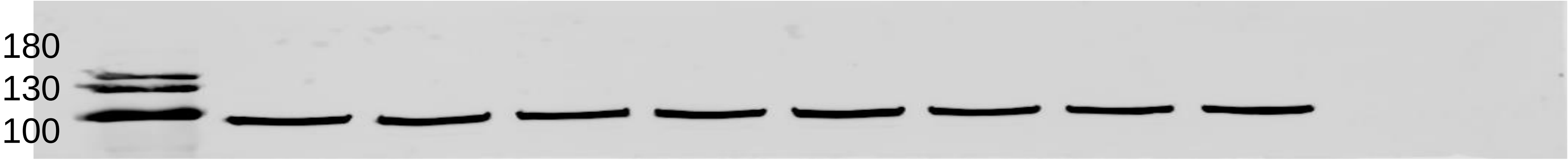

Original western blots of Figure 4

MIA PaCa-2 vs MIA PaCa-2$^{\Delta NOXA}$ → KH16 – 24h & 48h [ac-H3]

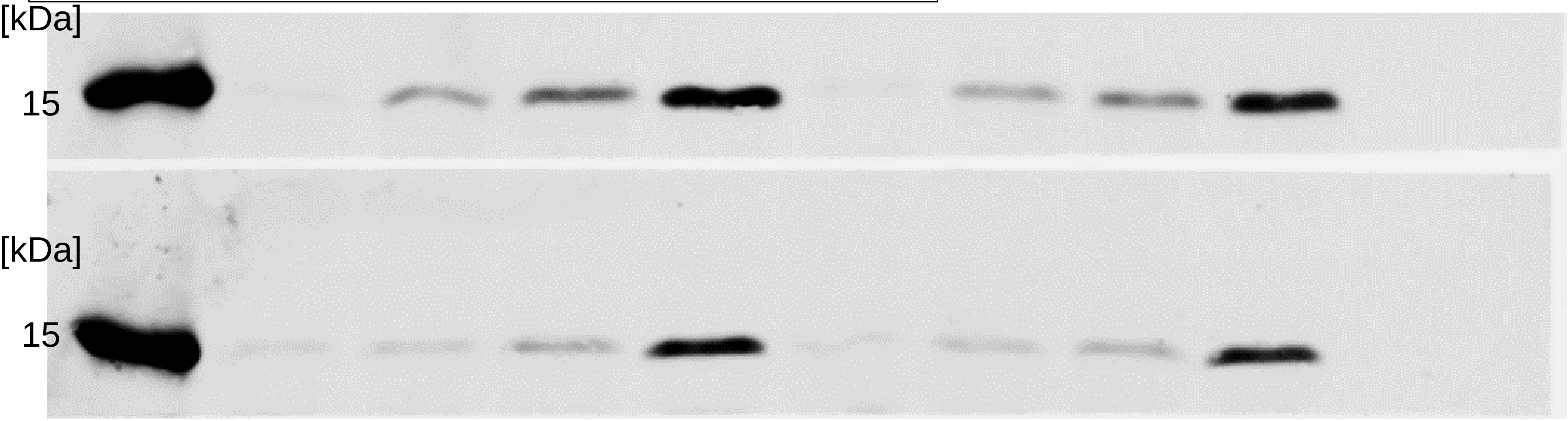

MIA PaCa-2 vs MIA PaCa-2$^{\Delta NOXA}$ → KH16 – 24h & 48h [HSP90 for ac-H3]

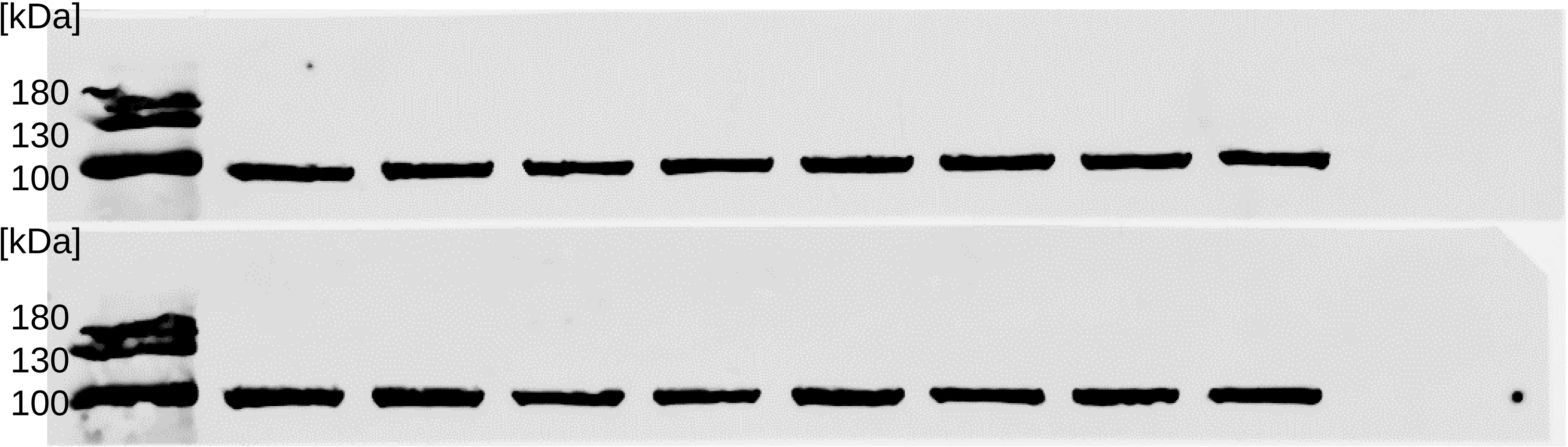

MIA PaCa-2 vs MIA PaCa-2$^{\Delta NOXA}$ → KH16 – 24h & 48h [p21]

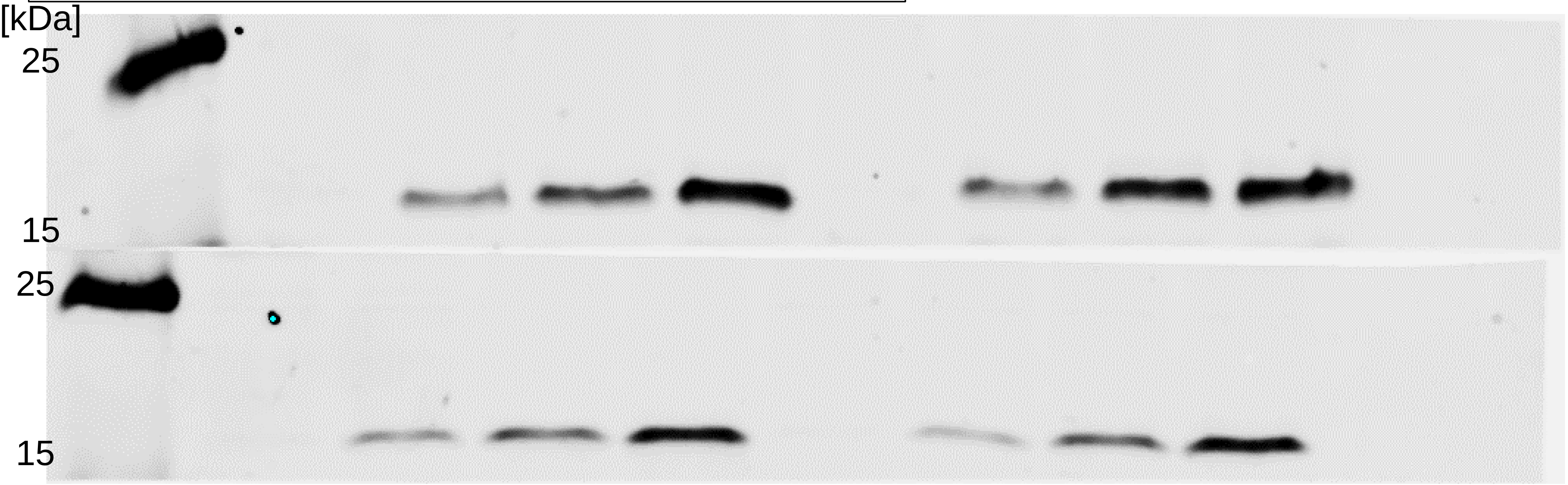

MIA PaCa-2 vs MIA PaCa-2$^{\Delta NOXA}$ → KH16 – 24h & 48h [HSP90 for p21]

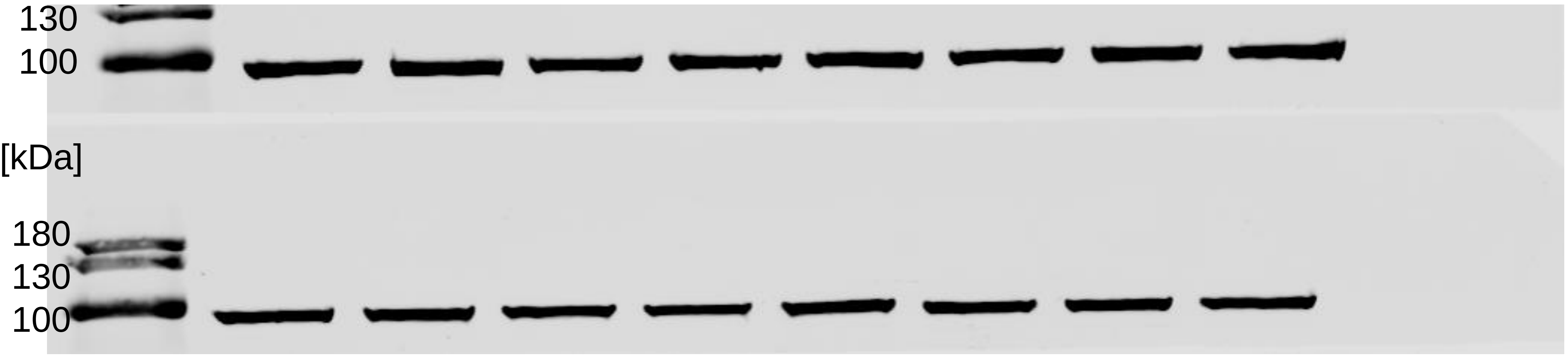

MIA PaCa-2 vs MIA PaCa-2$^{\Delta NOXA}$ → KH16 – 24h & 48h [cl.PARP1]

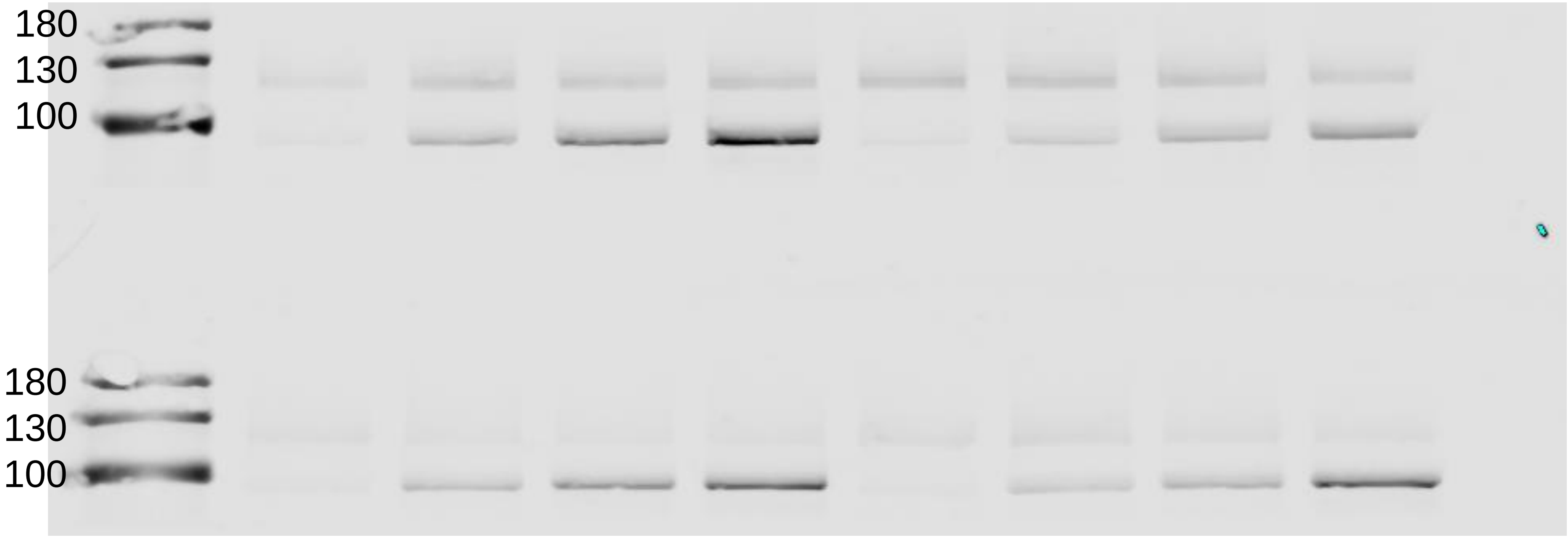

MIA PaCa-2 vs MIA PaCa-2$^{\Delta NOXA}$ → KH16 – 24h & 48h [GAPDH for cl.PARP1]

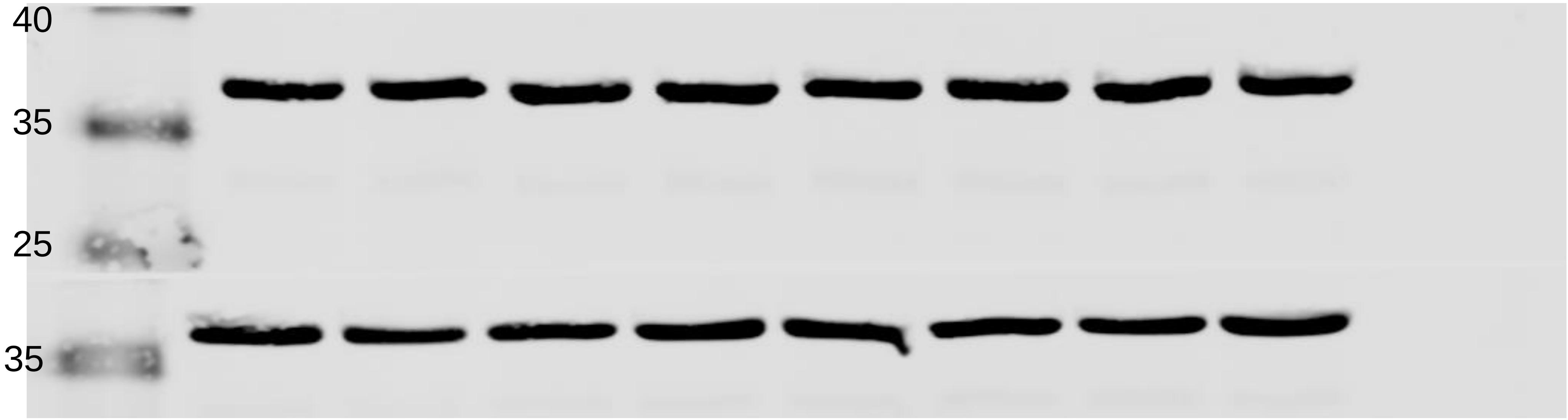

MIA PaCa-2 vs MIA PaCa-2$^{\Delta NOXA}$ → KH16 – 24h [cl.caspase-3]

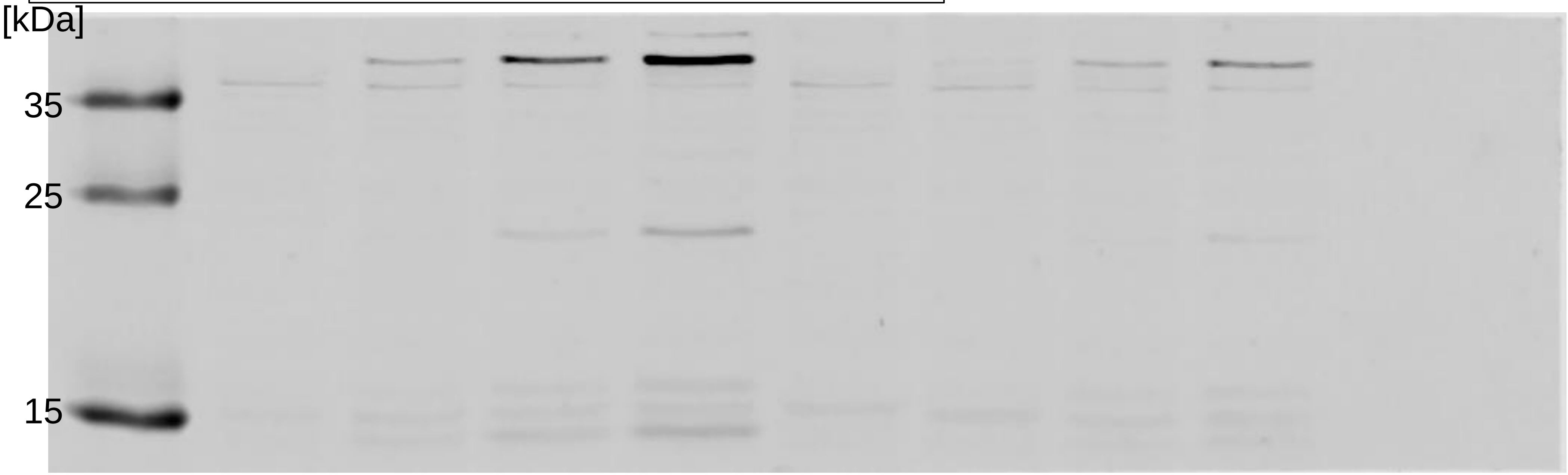

MIA PaCa-2 vs MIA PaCa-2$^{\Delta NOXA}$ → KH16 – 48h [cl.caspase-3]

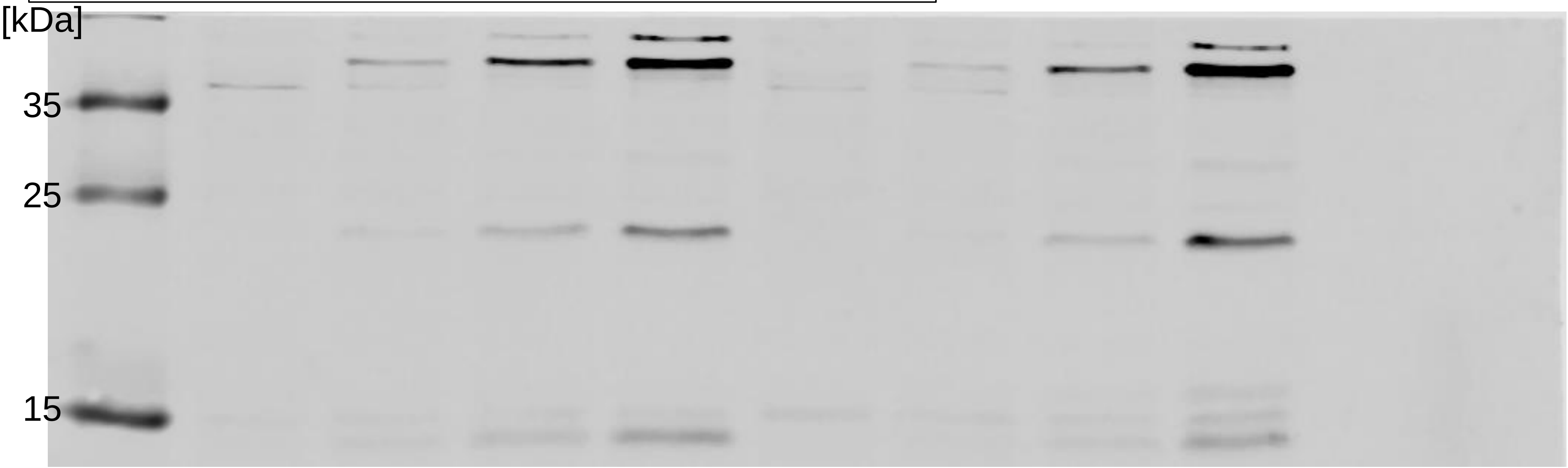

MIA PaCa-2 vs MIA PaCa-2$^{\Delta NOXA}$ → KH16 – 24h & 48h [HSP90 for cl.caspase-3]

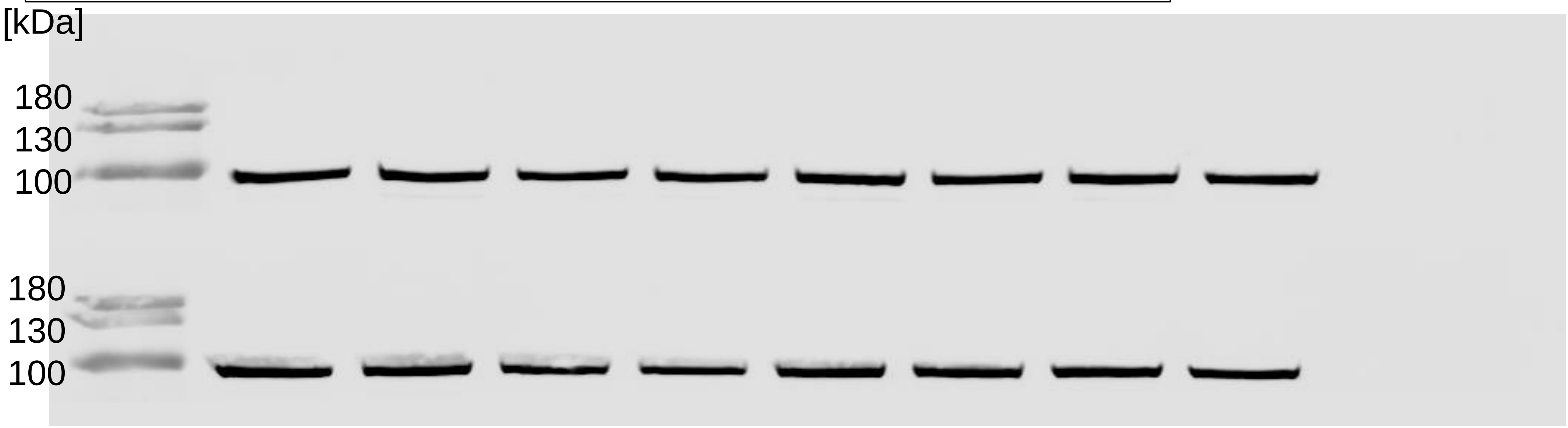

MIA PaCa-2 vs MIA PaCa-2$^{\Delta NOXA}$ → KH16 – 24h [BAX]

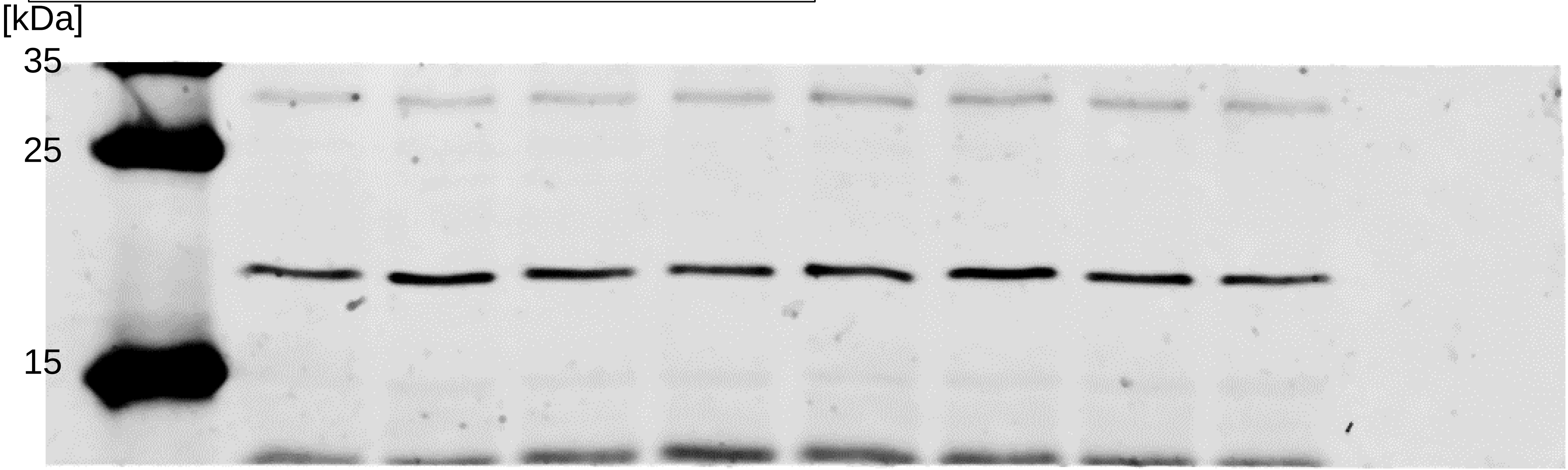

MIA PaCa-2 vs MIA PaCa-2$^{\Delta NOXA}$ → KH16 – 48h [BAX]

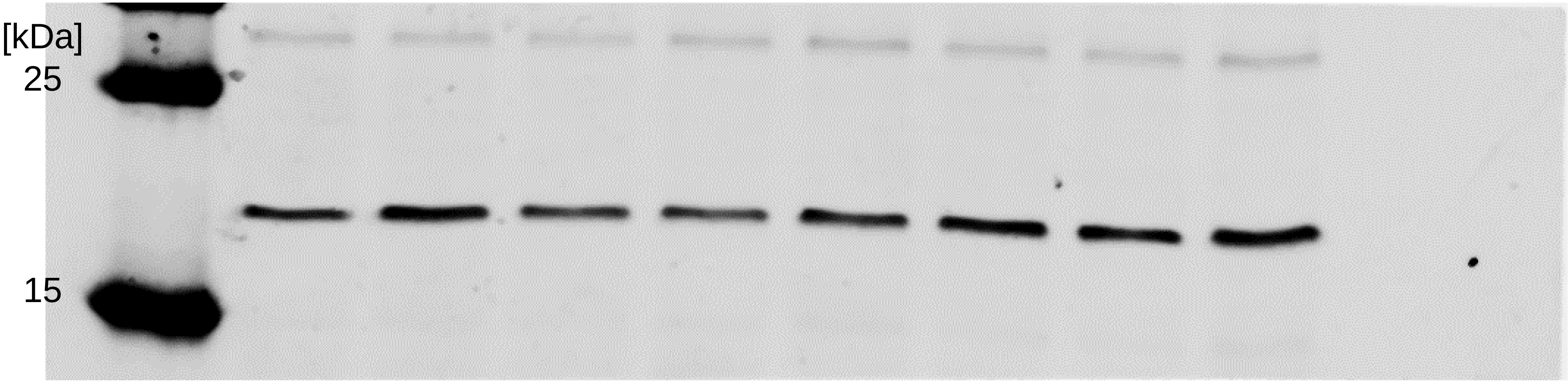

MIA PaCa-2 vs MIA PaCa-2$^{\Delta NOXA}$ → KH16 – 24h & 48h [HSP90 for BAX]

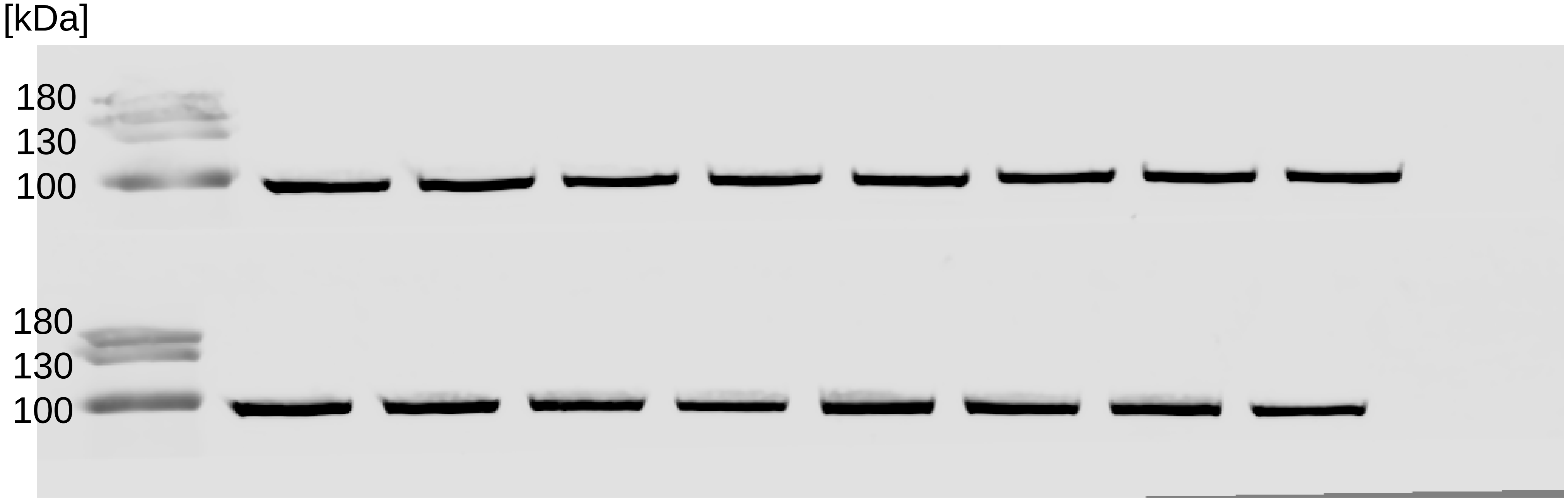

MIA PaCa-2 vs MIA PaCa-2$^{\Delta NOXA}$ → KH16 – 24h [NOXA]

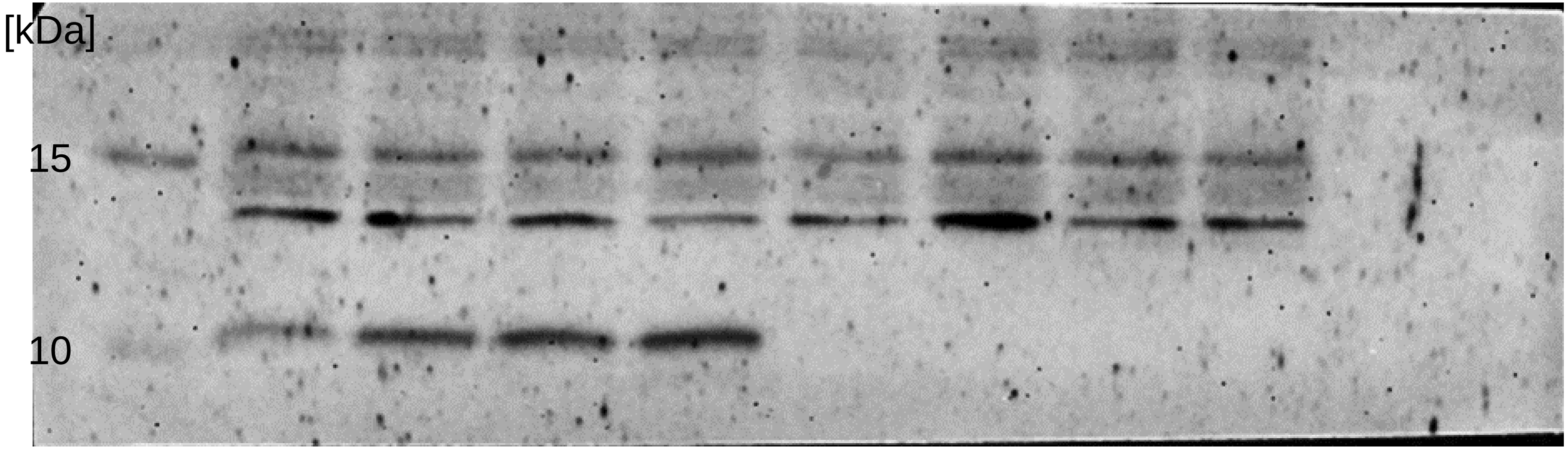

MIA PaCa-2 vs MIA PaCa-2$^{\Delta NOXA}$ → KH16 – 48h [NOXA]

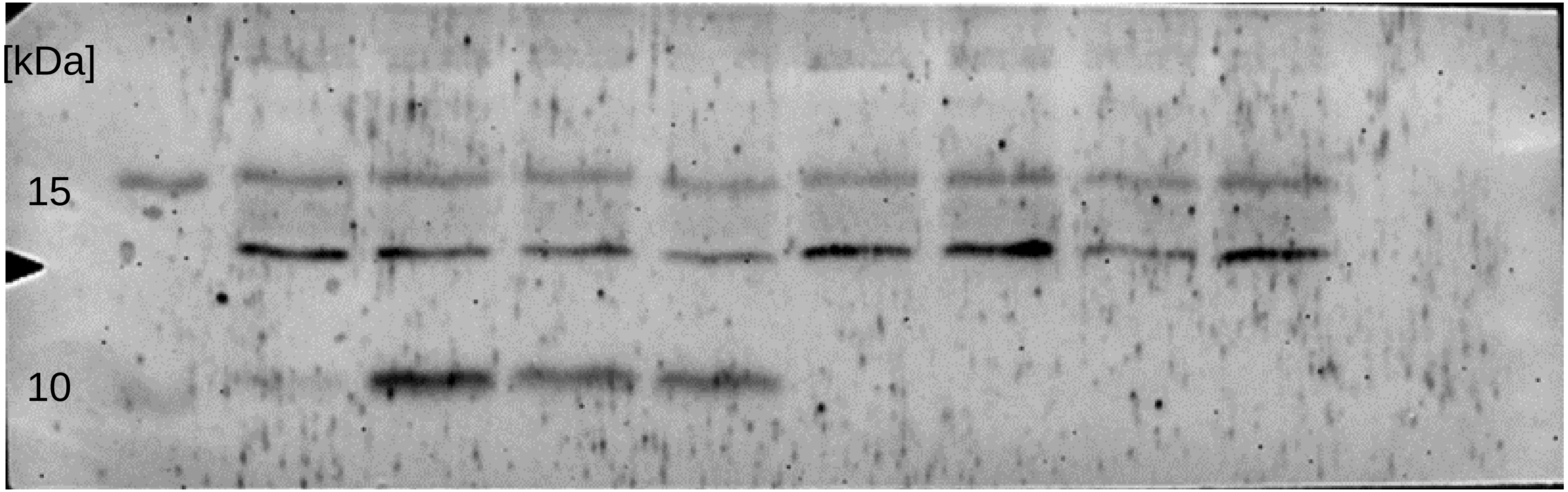

MIA PaCa-2 vs MIA PaCa-2$^{\Delta NOXA}$ → KH16 – 24h [GAPDH for NOXA]

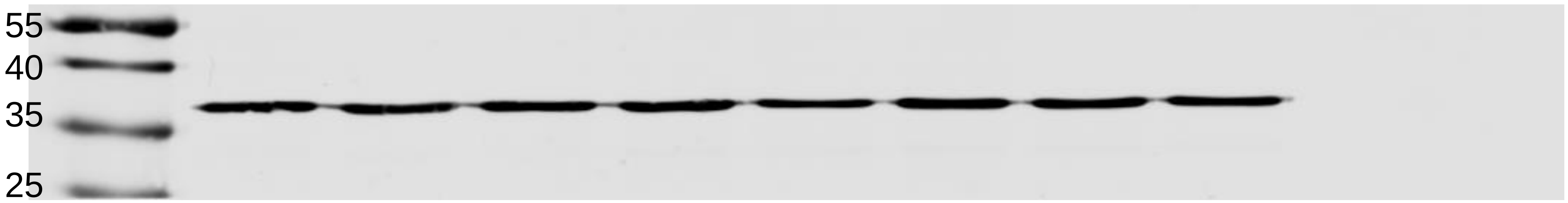

MIA PaCa-2 vs MIA PaCa-2$^{\Delta NOXA}$ → KH16 – 48h [GAPDH for NOXA]

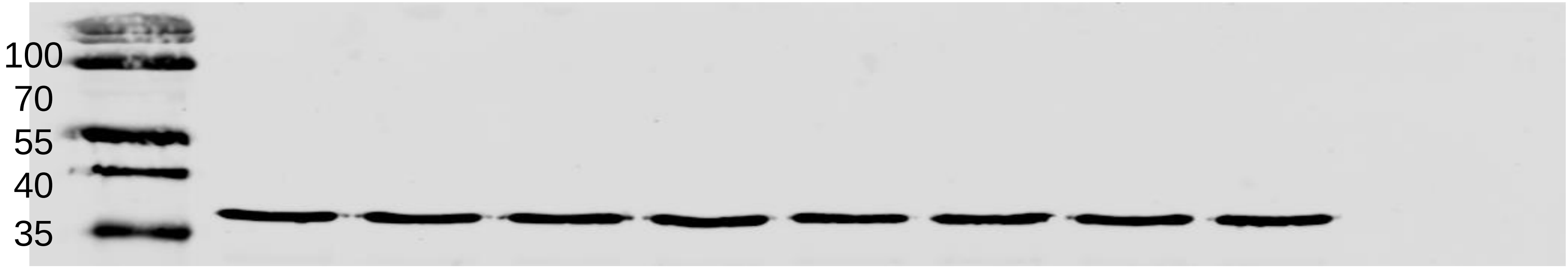

MIA PaCa-2 vs MIA PaCa-2$^{\Delta NOXA}$ → KH16 – 24h & 48h [BAK]

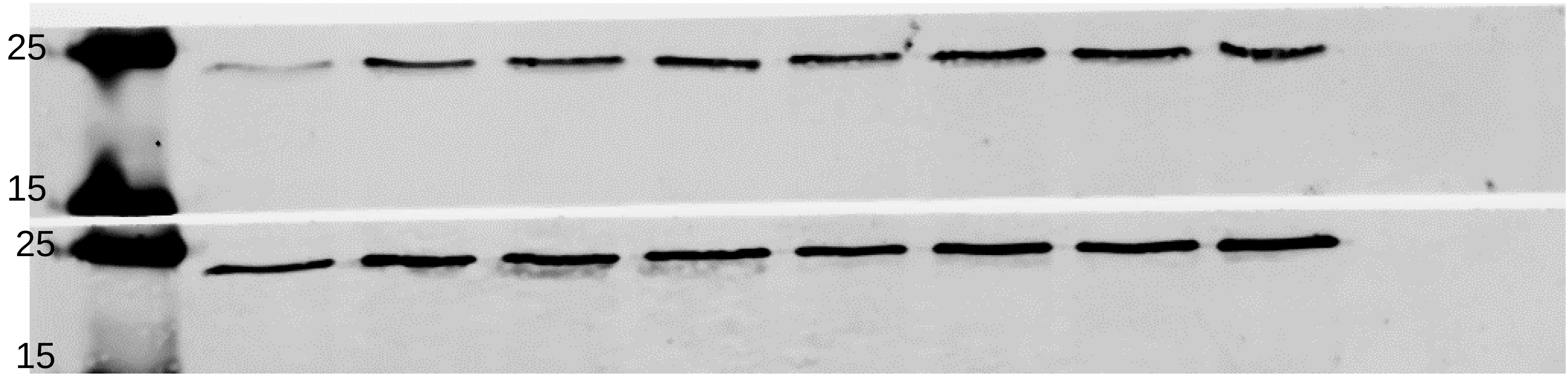

MIA PaCa-2 vs MIA PaCa-2$^{\Delta NOXA}$ → KH16 – 24h & 48h [GAPDH for BAK]

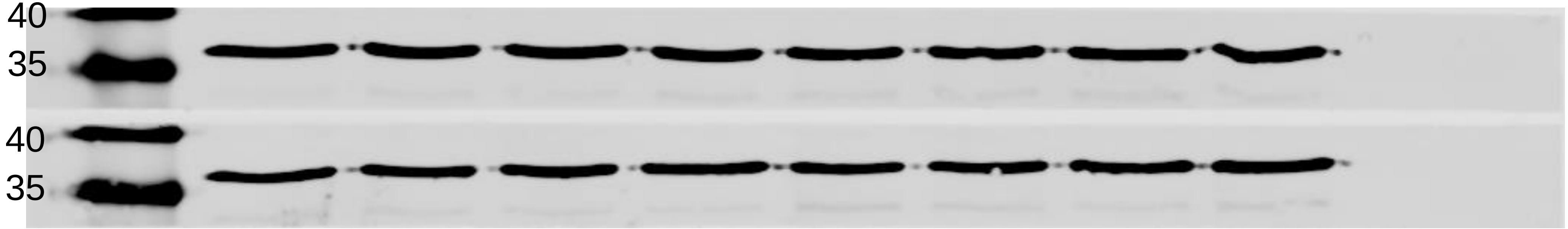

MIA PaCa-2 vs MIA PaCa-2$^{\Delta NOXA}$ → KH16 – 24h & 48h [BIM]

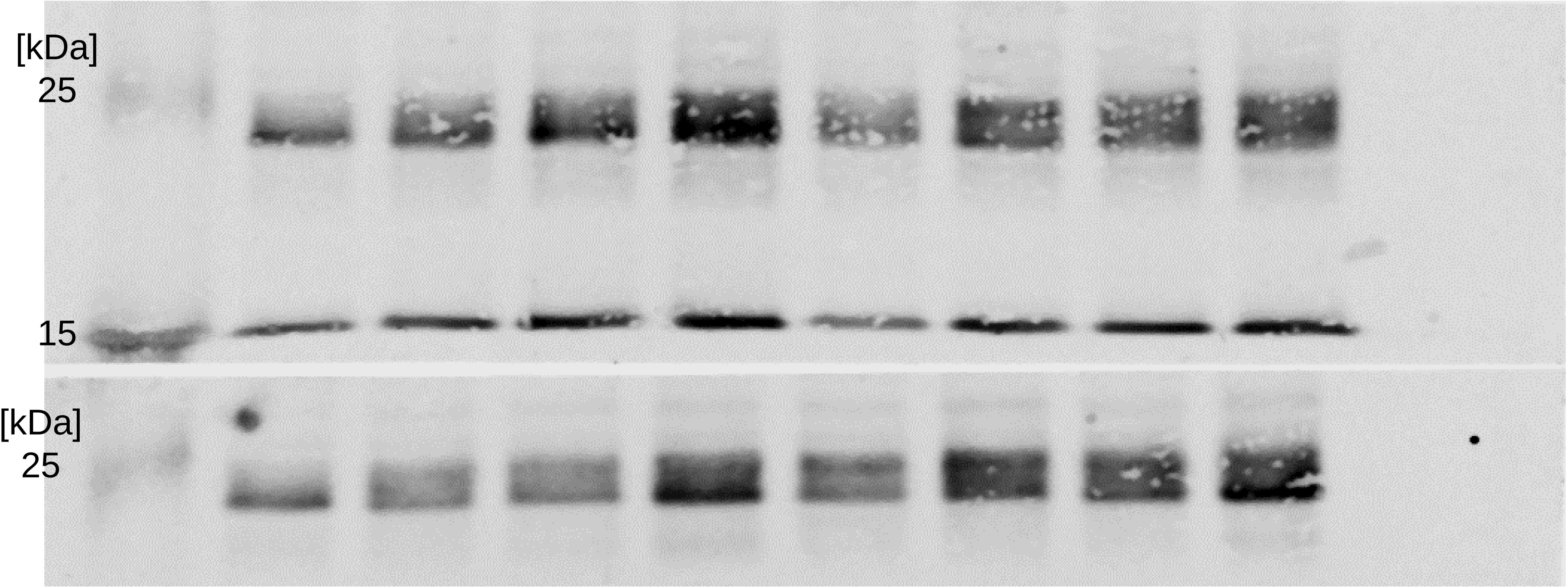

MIA PaCa-2 vs MIA PaCa-2$^{\Delta NOXA}$ → KH16 – 24h & 48h [vinculin for BIM]

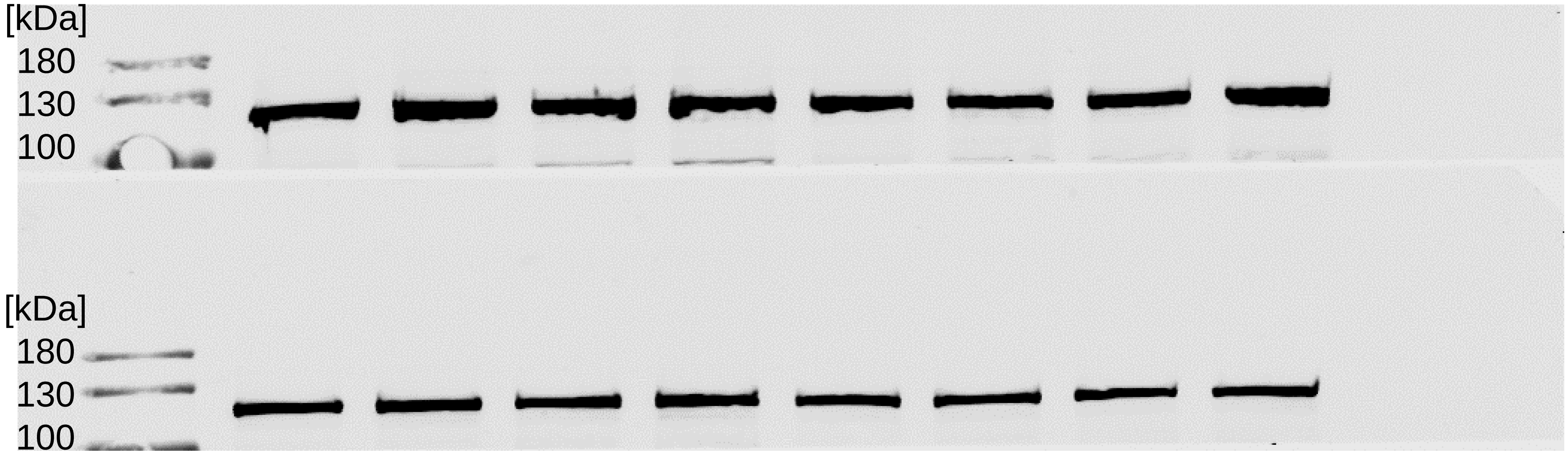

MIA PaCa-2 vs MIA PaCa-2$^{\Delta NOXA}$ → KH16 – 24h & 48h [BID]

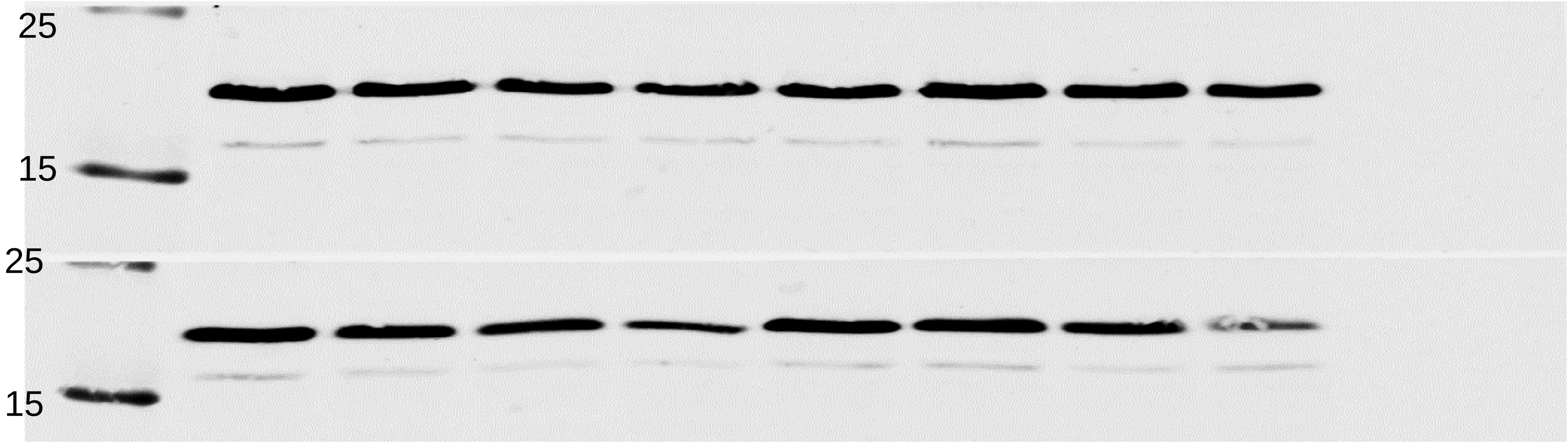

MIA PaCa-2 vs MIA PaCa-2$^{\Delta NOXA}$ → KH16 – 24h & 48h [MCL-1]

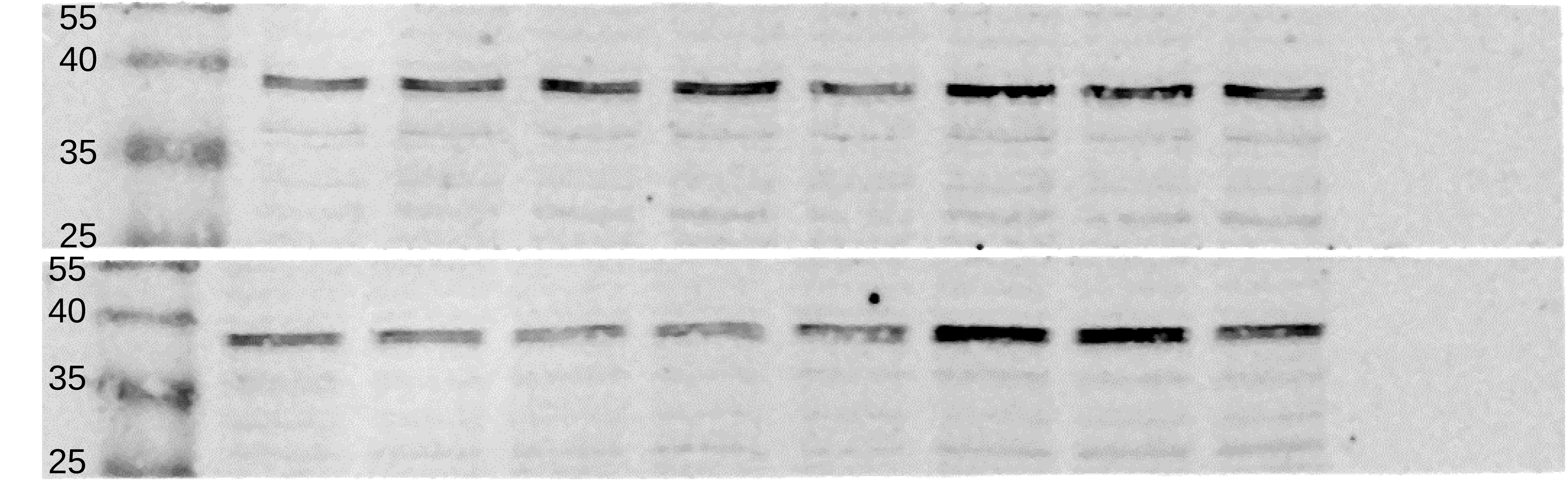

MIA PaCa-2 vs MIA PaCa-2$^{\Delta NOXA}$ → KH16 – 24h & 48h [HSP90 for BID & MCL-1]

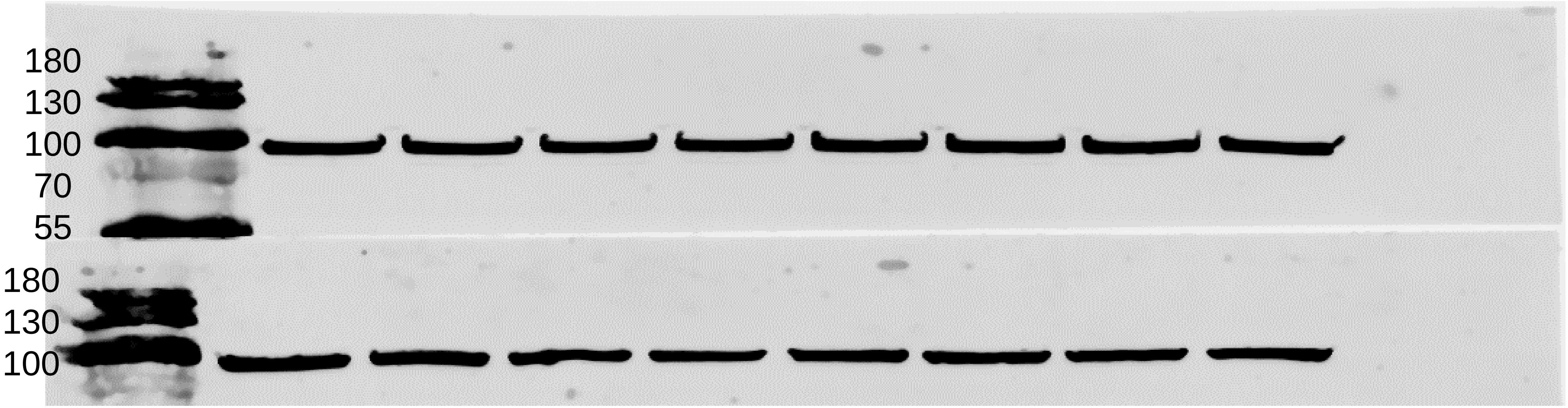

MIA PaCa-2 vs MIA PaCa-2$^{\Delta NOXA}$ → KH16 – 24h & 48h [BCL-XL]

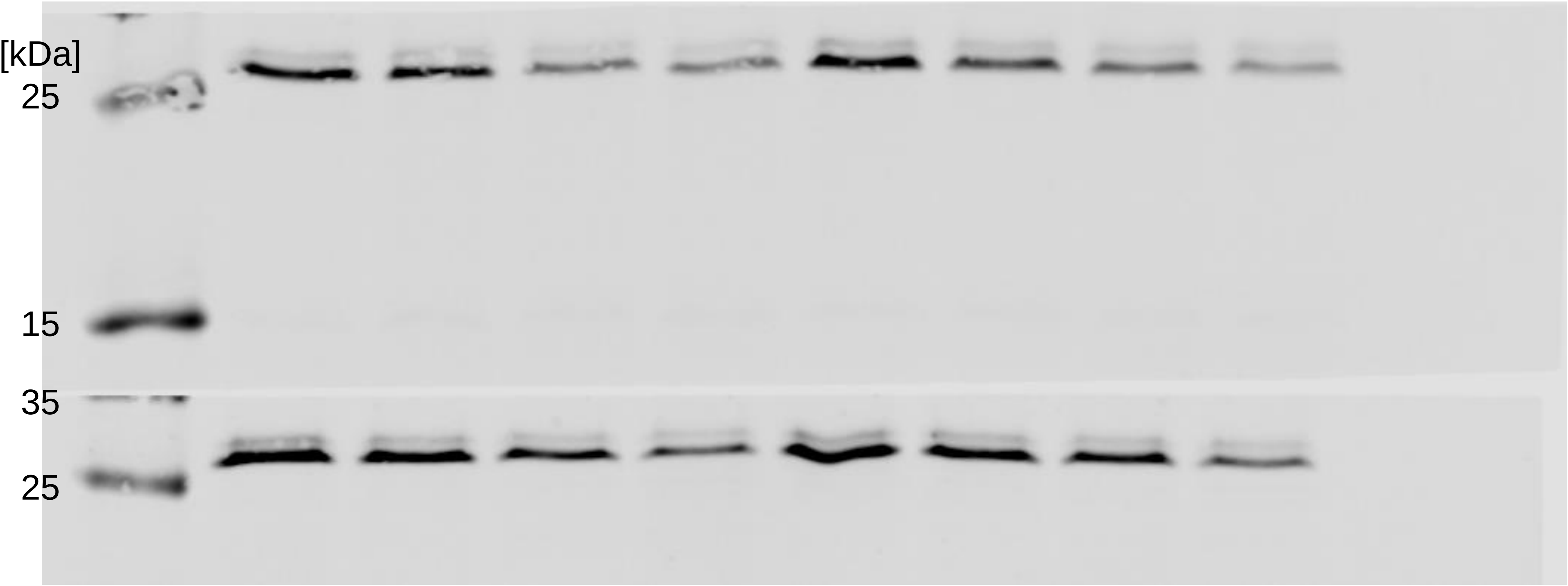

MIA PaCa-2 vs MIA PaCa-2$^{\Delta NOXA}$ → KH16 – 24h & 48h [HSP90 for BCL-XL]

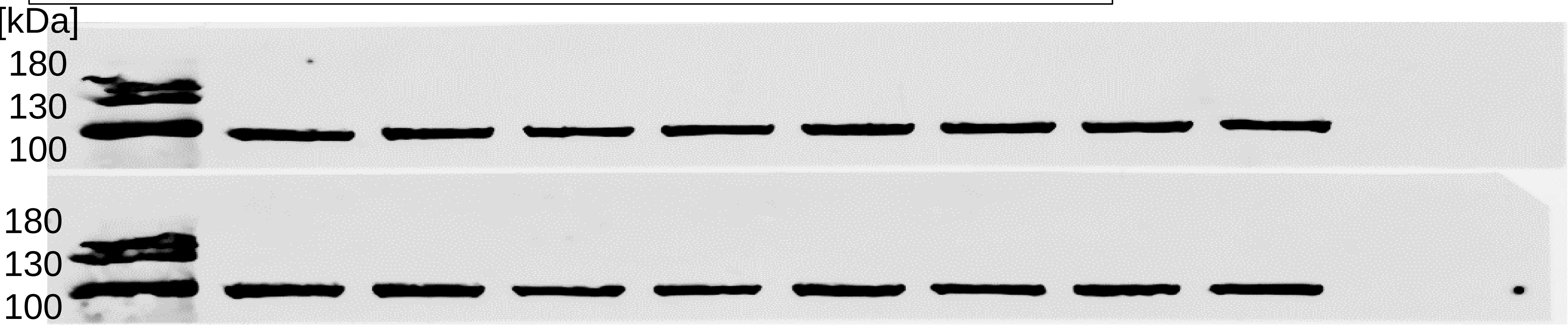

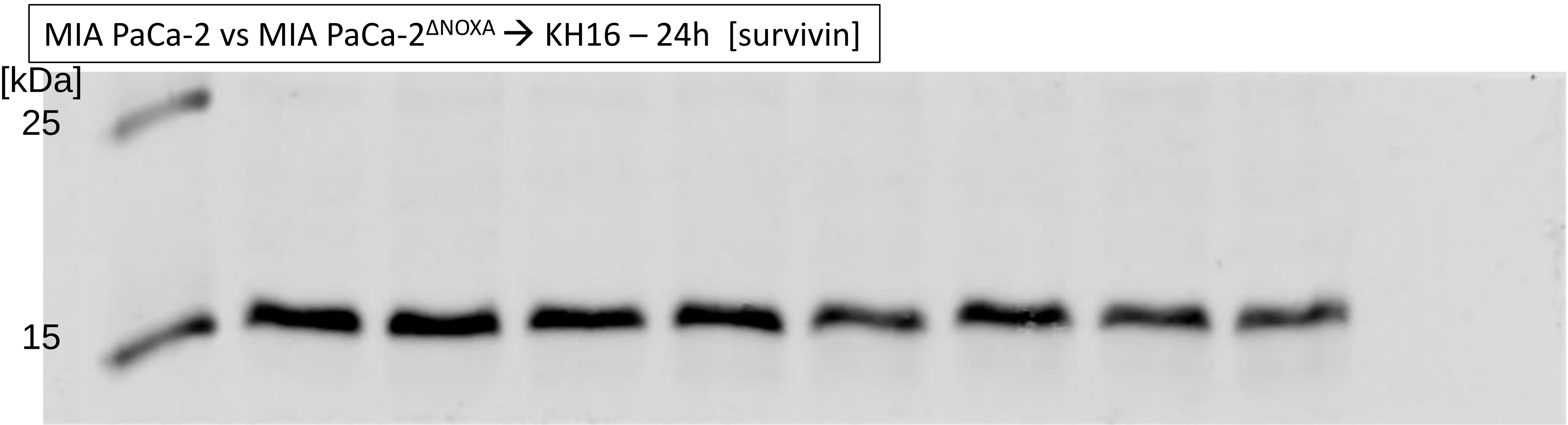
MIA PaCa-2 vs MIA PaCa-2$^{\Delta NOXA}$ → KH16 – 24h [survivin]
[kDa]
25
15

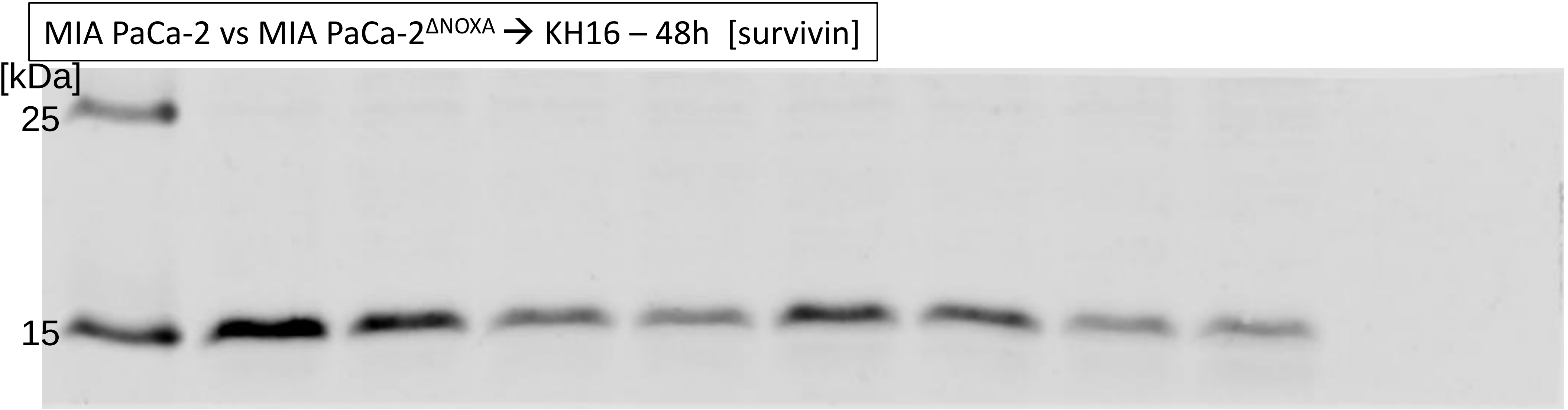
MIA PaCa-2 vs MIA PaCa-2$^{\Delta NOXA}$ → KH16 – 48h [survivin]
[kDa]
25
15

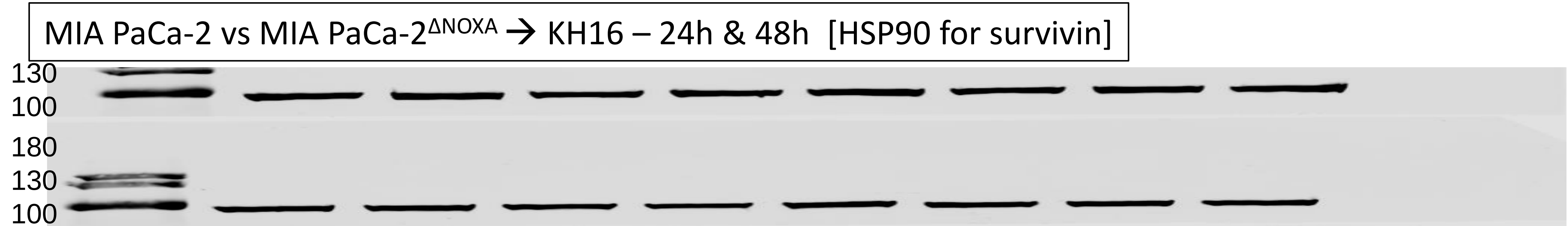
MIA PaCa-2 vs MIA PaCa-2$^{\Delta NOXA}$ → KH16 – 24h & 48h [HSP90 for survivin]
130
100
180
130
100

Original western blots of Figure S1

MIA PaCa-2 → KH16 [HDAC1]

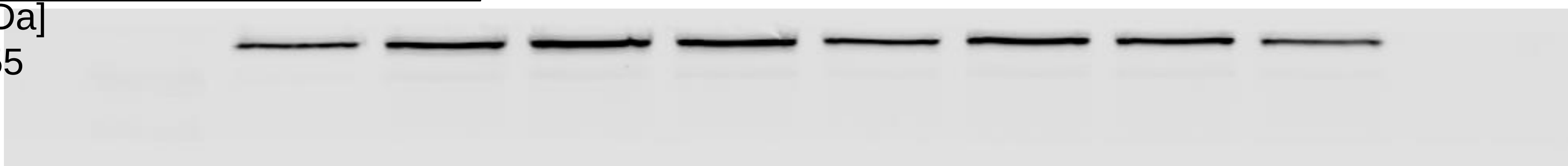

MIA PaCa-2 → KH16 [HSP90 for HDAC1 & HDAC2]

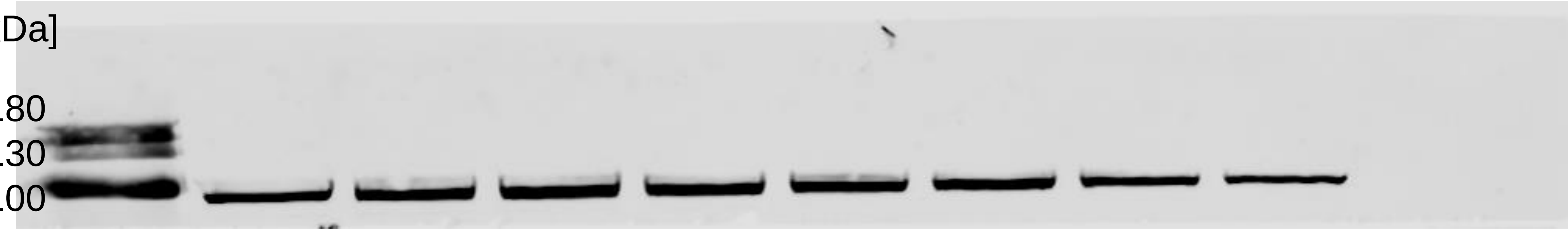

MIA PaCa-2 → KH16 [HDAC2]

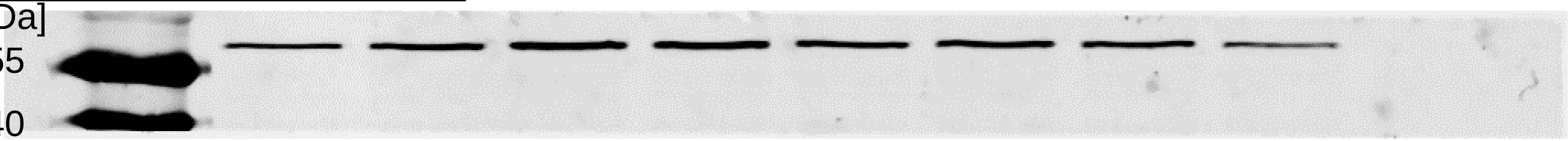

MIA PaCa-2 → KH16 [HDAC3]

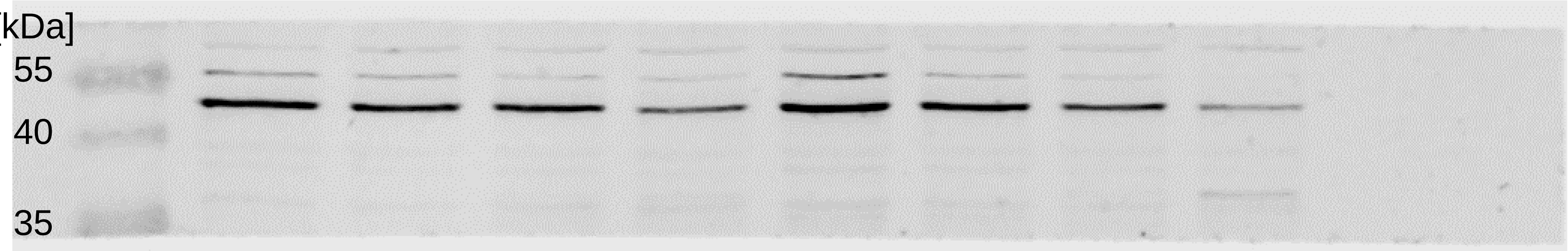

MIA PaCa-2 → KH16 [HSP90 for HDAC3 & HDAC8]

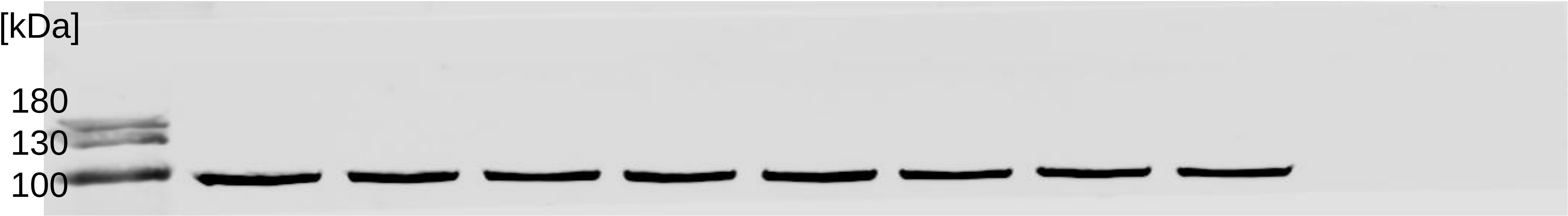

MIA PaCa-2 → KH16 [HDAC8]

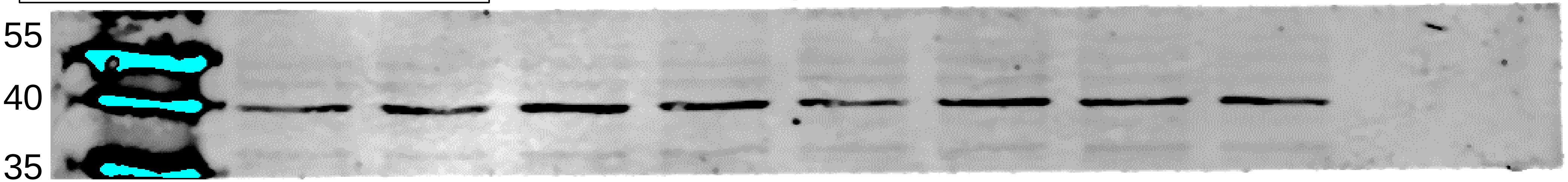

HROC80 → KH16 [HDAC1]

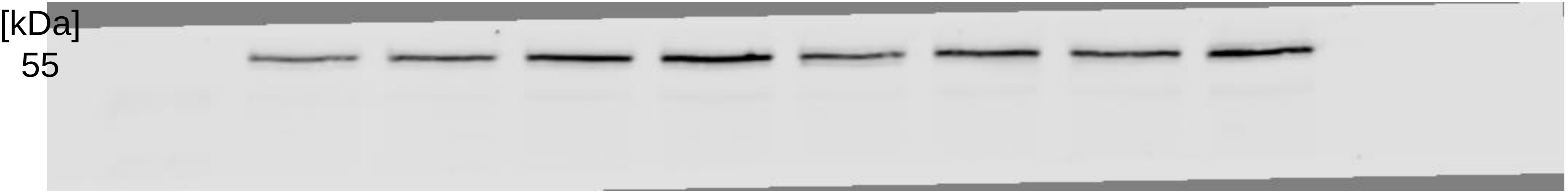

HROC80 → KH16 [HSP90 for HDAC1 & HDAC2]

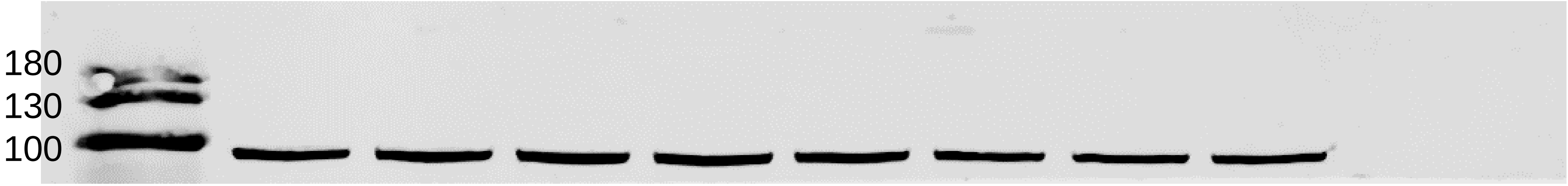

HROC80 → KH16 [HDAC2]

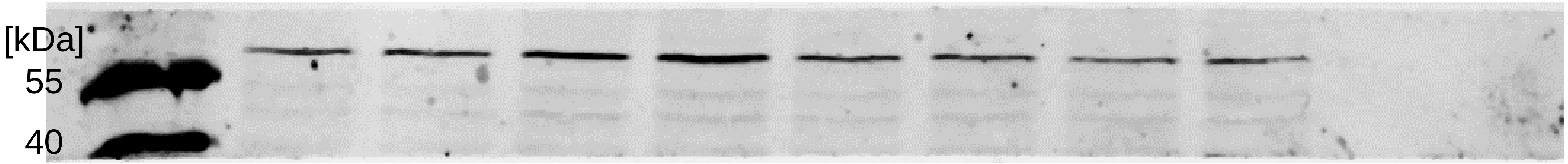

HROC80 → KH16 [HDAC3]

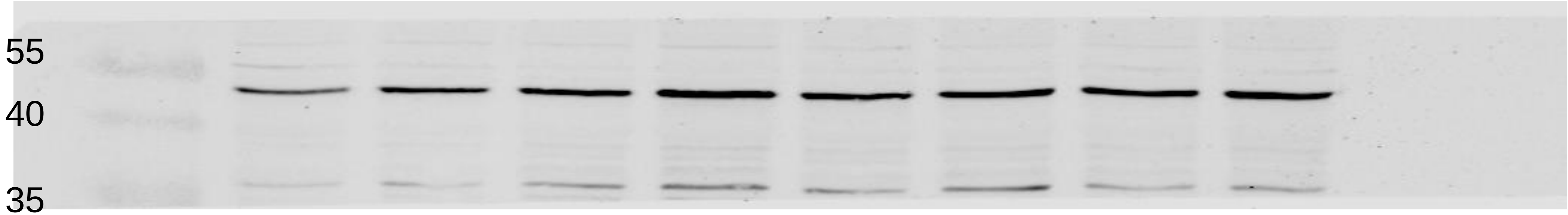

HROC80 → KH16 [HSP90 for HDAC3 & HDAC8]

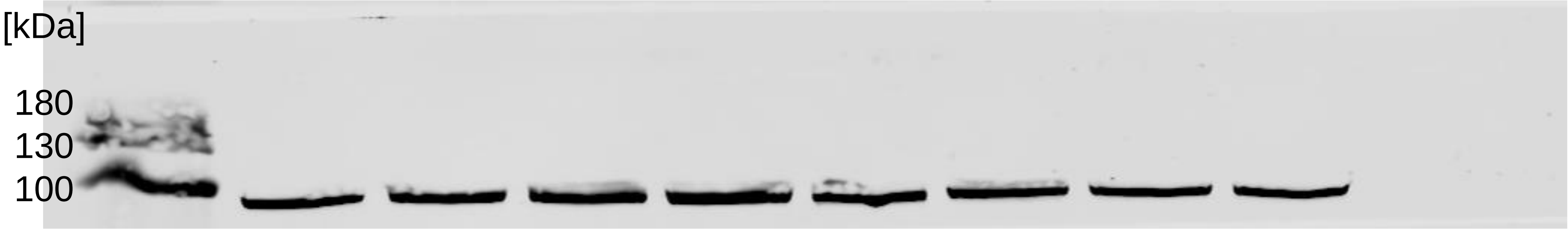

HROC80 → KH16 [HDAC8]

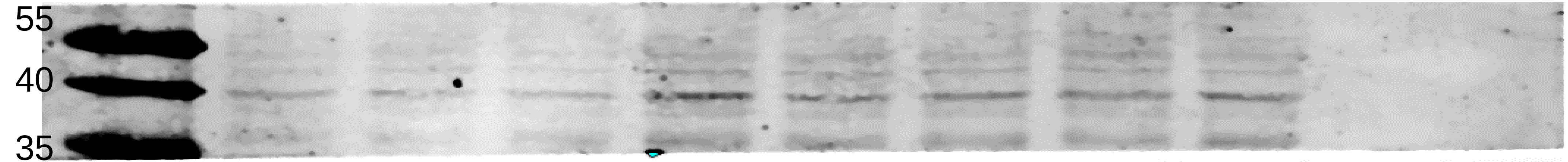

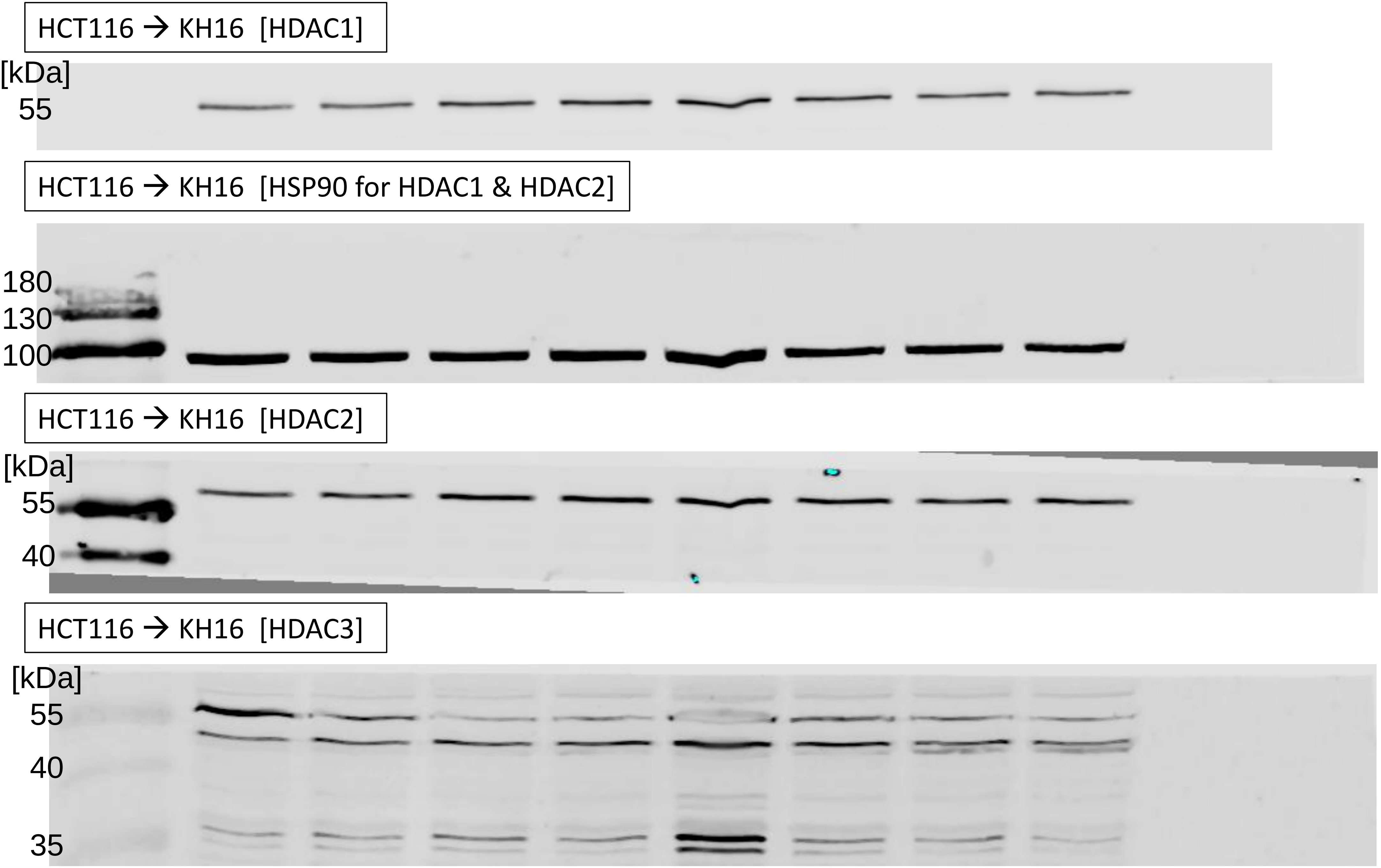
HCT116 → KH16 [HDAC1]
[kDa]
55
HCT116 → KH16 [HSP90 for HDAC1 & HDAC2]
180
130
100
HCT116 → KH16 [HDAC2]
[kDa]
55
40
HCT116 → KH16 [HDAC3]
[kDa]
55
40
35

HCT116 → KH16 [HDAC8]

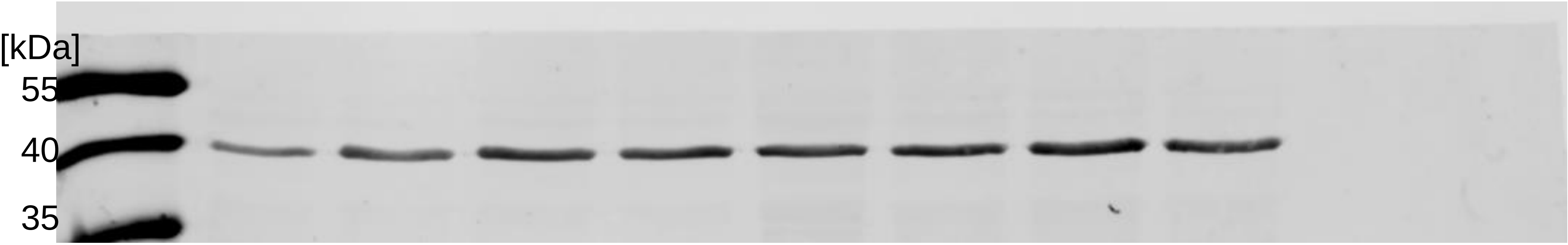

HCT116 → KH16 [HSP90 for HDAC3 & HDAC8]

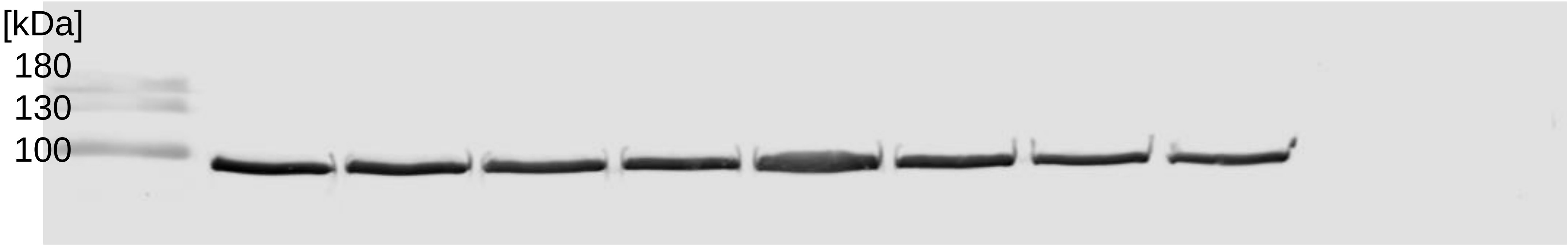

Original western blots of Figure S2

MIA PaCa-2 → siNOXA ± KH16 [NOXA]

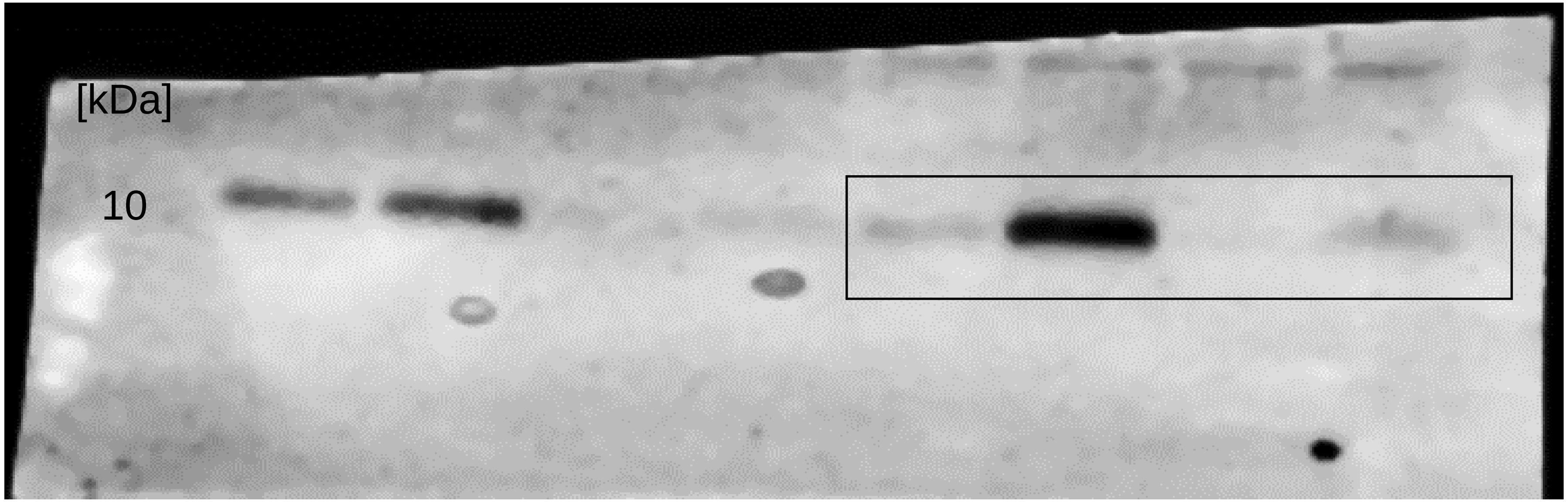

MIA PaCa-2 → siNOXA ± KH16 [HSP90 for NOXA]

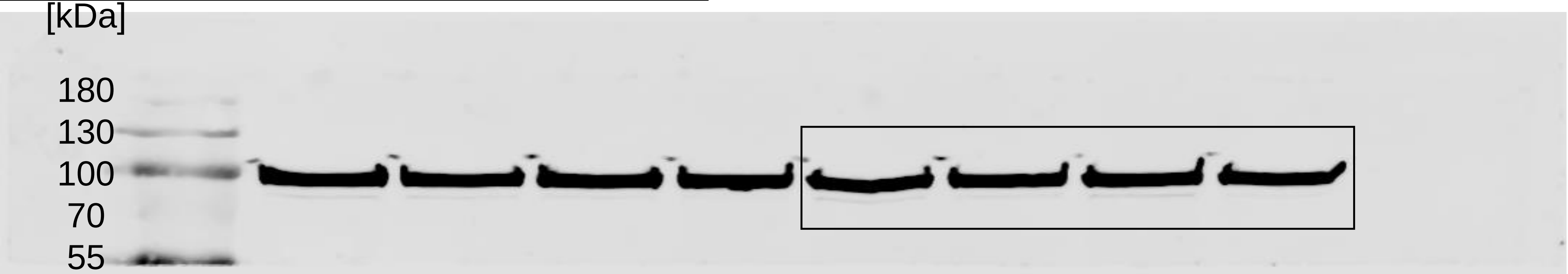

Supplement: Supplementary file 1 [file cancers-15-03650-s001.zip › cancers-2344362-supplementary File S1. Original western blots.pdf]
